# Estrogen receptor beta repurposes EZH2 to suppress oncogenic NFκB/p65 signaling in triple negative breast cancer

**DOI:** 10.1038/s41523-022-00387-0

**Published:** 2022-02-17

**Authors:** Kirsten G. M. Aspros, Jodi M. Carter, Tanya L. Hoskin, Vera J. Suman, Malayannan Subramaniam, Michael J. Emch, Zhenqing Ye, Zhifu Sun, Jason P. Sinnwell, Kevin J. Thompson, Xiaojia Tang, Esther P. B. Rodman, Xiyin Wang, Adam W. Nelson, Igor Chernukhin, Feda H. Hamdan, Elizabeth S. Bruinsma, Jason S. Carroll, Martin E. Fernandez-Zapico, Steven A. Johnsen, Krishna R. Kalari, Haojie Huang, Roberto A. Leon-Ferre, Fergus J. Couch, James N. Ingle, Matthew P. Goetz, John R. Hawse

**Affiliations:** 1 Department of Biochemistry and Molecular Biology, Mayo Clinic, Rochester, MN 55905 USA; 2 Department of Laboratory Medicine and Pathology, Mayo Clinic, Rochester, MN 55905 USA; 3 Department of Health Sciences Research, Mayo Clinic, Rochester, MN 55905 USA; 4 Cancer Research UK Cambridge Research Institute, University of Cambridge, Cambridge, UK; 5 Gene Regulatory Mechanisms and Molecular Epigenetics Lab, Division of Gastroenterology and Hepatology, Mayo Clinic, Rochester, MN 55905 USA; 6 Shulze Center for Novel Therapeutics, Division of Oncology Research, Mayo Clinic, Rochester, MN 55905 USA; 7 Department of Urology, Mayo Clinic, Rochester, MN 55905 USA; 8 Department of Oncology, Mayo Clinic, Rochester, MN 55905 USA

**Keywords:** Breast cancer, Targeted therapies

## Abstract

Triple Negative Breast Cancer (TNBC) accounts for 15–20% of all breast cancer cases, yet is responsible for a disproportionately high percentage of breast cancer mortalities. Thus, there is an urgent need to identify novel biomarkers and therapeutic targets based on the molecular events driving TNBC pathobiology. Estrogen receptor beta (ERβ) is known to elicit anti-cancer effects in TNBC, however its mechanisms of action remain elusive. Here, we report the expression profiles of ERβ and its association with clinicopathological features and patient outcomes in the largest cohort of TNBC to date. In this cohort, ERβ was expressed in approximately 18% of TNBCs, and expression of ERβ was associated with favorable clinicopathological features, but correlated with different overall survival outcomes according to menopausal status. Mechanistically, ERβ formed a co-repressor complex involving enhancer of zeste homologue 2/polycomb repressive complex 2 (EZH2/PRC2) that functioned to suppress oncogenic NFκB/RELA (p65) activity. Importantly, p65 was shown to be required for formation of this complex and for ERβ-mediated suppression of TNBC. Our findings indicate that ERβ+ tumors exhibit different characteristics compared to ERβ− tumors and demonstrate that ERβ functions as a molecular switch for EZH2, repurposing it for tumor suppressive activities and repression of oncogenic p65 signaling.

## Introduction

Breast cancer is the most prevalent cancer among women worldwide^1^. Triple negative breast cancer (TNBC) accounts for 15-20% of breast cancer cases and is defined by the lack of estrogen receptor alpha (ERα) and progesterone receptor (PR) expression and the absence of Her2 overexpression/amplification. TNBC is a heterogeneous disease and represents the most aggressive subtype of breast cancer, accounting for a disproportionately high fraction of breast cancer mortalities^2^. Current standard of care for newly diagnosed TNBC patients involves a combination of chemotherapy, radiation, and surgery^3^. Unfortunately, approximately 46% of TNBC patients will develop distant metastasis, and survival time after diagnosis of metastatic disease is grim, with a median of only 13.3 months^3^. Further complicating the care of TNBC patients is the lack of treatment strategies that can be used in the adjuvant setting to prevent disease recurrence^3^. For these reasons, it is imperative to better understand the biology of TNBC and to discover and develop novel therapeutic strategies to combat this deadly disease.

Endocrine-based therapies such as tamoxifen and aromatase inhibitors remain the most utilized and effective class of drugs for the treatment of ERα-positive breast cancer but are not used to treat TNBC due to lack of ERα expression. Approaches to target other hormone receptors in TNBC such as the glucocorticoid receptor (GR) and androgen receptor (AR) are being explored with some early signs of success in a proportion of patients^4,5^. A second form of the estrogen receptor, ERβ, is reported to be expressed in approximately 20-30% of TN breast tumors^6,7^. ERβ is largely thought to function as a tumor suppressor in the breast given its high expression in normal mammary epithelium and loss of expression in most tumors^8,9^. Ligand-mediated activation of ERβ is known to elicit anti-cancer activity in TNBC cell lines in vitro and in vivo, effects that are reversed by anti-estrogens^6,10–12^. Intriguingly, estrogen remains the only known drug to not only prevent breast cancer development, but to also reduce breast cancer mortality^13,14^. It is conceivable that ERβ plays a central role in mediating these clinical benefits of estrogen, a possibility that has not yet been explored. Therefore, a better understanding of ERβ in breast cancer is required. Further, elucidation of ERβ’s expression profiles, role in pathophysiology, and molecular mechanisms of action are likely to uncover novel biological processes that could be exploited for therapeutic purposes.

Here we report the incidence of ERβ protein expression in the largest cohort of centrally reviewed TN breast tumors to date^15^, as well as its association with clinicopathological features, TNBC subtypes and patient outcomes. Using multiple model systems and unbiased global screens, we have discovered a molecular mechanism by which ERβ suppresses TNBC proliferation and migration. Specifically, ERβ is shown to potently inhibit the oncogenic transcriptional activities of nuclear factor kappa b (NFκB/RELA/p65) by repurposing enhancer of zeste homologue 2 (EZH2) from a co-activator to a co-repressor of p65. The functions of this co-repressor complex rely on expression of p65 and the methyltransferase activity of EZH2 for ERβ to elicit anti-cancer effects. These results describe new biology of ERβ in TNBC and further our understanding of its mechanisms of action in this sub-type of the disease.

## Results

### ERβ expression in TNBC and its association with clinicopathological features

ERβ protein expression was assessed in 567 triple negative breast tumors from the Mayo Clinic TNBC Cohort^15^ using the well-validated PPG5/10 ERβ monoclonal antibody^16–18^. ERβ positivity (ERβ+) was defined by moderate or strong nuclear staining in at least 25% of tumor cells. Based on this definition, ERβ was expressed in 102 of the 567 tumors (18%) (Table 1). Representative images of ERβ+ and ERβ− tumors have been previously published^16^. None of the patients included in this cohort received neo-adjuvant therapy. Patients with ERβ+ tumors were less likely to present with lymph node involvement than those with ERβ-negative (ERβ−) tumors (26.0% in ERβ+ versus 37.1% in ERβ−; *p* = 0.04). The proportion of patients with 15% or more stromal tumor infiltrating lymphocytes (TILs) was greater in ERβ− tumors (43.1% in ERβ+ versus 55.6% in ERβ−; *p* = 0.03) (Table 1). Regarding treatment, patients with ERβ+ tumors were less likely to receive aggressive regimens of chemotherapy (Anthracycline + Taxane) (8.8% in ERβ+ versus 22.2% in ERβ−; *p* = 0.009) (Table 1). ERβ expression was not found to differ with respect to age (*p* = 0.48), BMI (*p* = 0.84), histologic subtype (*p* = 0.23), grade (*p* = 0.39), tumor size (*p* = 0.31), Ki67 (*p* = 0.80), or AR expression (*p* = 0.87) (Table 1). Regarding overall survival (OS), there were 58 deaths among the 215 pre-menopausal women. After accounting for age, tumor size, nodal positivity and receipt of adjuvant chemotherapy, pre-menopausal women with ERβ+ disease were more likely to have shorter OS than pre-menopausal women with ERβ− disease (HRadj = 2.06; 95% CI: 1.14–3.71; *p* = 0.017). Among 342 post-menopausal women, there were 138 deaths. After accounting for the same variables, OS was not found to differ significantly with respect to ERβ status (HRadj = 0.96; 95% CI: 0.63–1.48; *p* = 0.867).

**Table 1:** Mayo clinic TNBC cohort characteristics stratified by ERβ expression.

Characteristics	Range	ERβ positive (*n* = 102)	ERβ negative (*n* = 465)	*p* value
Age (years)	<50	29 (28.4%)	161 (34.6%)	0.48
	50–69	54 (52.9%)	221 (47.5%)	
	>70	19 (18.6%)	83 (17.8%)	
BMI (kg/m^2^)	<25	40 (39.6%)	171 (37.3%)	0.84
	25–29.9	32 (31.7%)	159 (34.7%)	
	≥30	29 (28.7%)	128 (27.9%)	
	Missing	1	7	
Histology	Apocrine differentiation	6 (5.9%)	29 (6.2%)	0.23
	Medullary features	10 (9.8%)	83 (17.8%)	
	Metaplastic carcinoma	9 (8.8%)	35 (7.5%)	
	Other	77 (75.5%)	318 (68.4%)	
Nottingham grade	1–2	14 (13.7%)	50 (10.8%)	0.39
	3	88 (86.3%)	415 (89.2%)	
Maximum tumor dimension (cm)	at most 2.0	59 (57.8%)	230 (49.6%)	0.31
	2.1–5.0	38 (37.3%)	210 (45.3%)	
	5.1 or larger	5 (4.9%)	24 (5.2%)	
	Not stated	0	1	
Lymph node involvement	positive	26 (26.0%)	170 (37.1%)	0.04
	negative	74 (74.0%)	288 (62.9%)	
	not evaluated	2	7	
Ki67	≤15%	22 (22.0%)	106 (23.5%)	0.80
	>15%	78 (78.0%)	346 (76.5%)	
	not obtained	2	13	
Androgen Receptor	0	55 (67.1%)	273 (67.4%)	0.87
	1–15%	5 (6.1%)	20 (4.9%)	
	≥15%	22 (26.8%)	112 (27.7%)	
	not obtained	20	60	
Stromal TILs	1–15%	58 (56.9%)	204 (44.4%)	0.03
	≥15%	44 (43.1%)	255 (55.6%)	
	not obtained	0	6	
Chemotherapy type	Anthracycline-based	26 (25.5%)	96 (20.7%)	0.009
	Anthracycline/Taxane	9 (8.8%)	103 (22.2%)	
	Taxane-based	4 (3.9%)	6 (1.3%)	
	Non-Anthracycline/non-Taxane	14 (13.7%)	58 (12.5%)	
	None/unknown	49 (48.0%)	202 (43.4%)	

*BMI* Body Mass Index, *TILs* Tumor Infiltrating Lymphocytes.

### ERβ expression among distinct subtypes of TNBC

TNBC is a highly heterogeneous disease that has been subtyped by various groups^19–21^. We therefore assessed the distribution of ERβ+ and ERβ− tumors across these different subtypes. In the four subtypes described using the non-negative matrix factorization (NMF) method^19^, the majority of ERβ+ tumors were classified as basal-like immune activated (BLIA) (34.4% of all ERβ+ tumors) or basal-like immunosuppressed (BLIS) (43.8% of all ERβ+ tumors), and no differences were observed between the distribution of ERβ+ versus ERβ− tumors across these four subtypes (*p* = 0.891) (Table 2). In the subtypes described by Jezequel et al. (Fuzzy)^21^, most ERβ+ tumors were classified as basal TNBC (bTNBC) (53.1%), but again no differences in the distribution of ERβ+ versus ERβ− tumors across subtypes were detected (*p* = 0.596) (Table 2). Last, using a classification system being developed by the Kalari group (CALAR), the vast majority of all tumors were classified as basal (bTNBC) (80.9%), and no significant differences were detected for the distribution of ERβ+ versus ERβ− cancers (*p* = 0.596) (Table 2). These data demonstrate that ERβ positivity is not significantly enriched within a particular subtype of TNBC.

**Table 2:** Distribution of ERβ+ and ERβ− TN tumors across TNBC subtypes.

Panel	Subtype	ERβ-positive (*n* = 32)	ERβ-negative (*n* = 225)	Total (*n* = 257)	*p* value
NMF					0.891
	BLIA	11 (34.4%)	83 (36.9%)	94 (36.6%)	
	BLIS	14 (43.8%)	87 (38.7%)	101 (39.3%)	
	LAR	5 (15.6%)	33 (14.7%)	38 (14.8%)	
	MES	2 (6.2%)	22 (9.8%)	24 (9.3%)	
Fuzzy					0.596
	IMM	10 (31.2%)	91 (40.4%)	101 (39.3%)	
	LAR	5 (15.6%)	33 (14.7%)	38 (14.8%)	
	bTNBC	17 (53.1%)	101 (44.9%)	118 (45.9%)	
CALAR					0.596
	LAR	5 (15.6%)	44 (19.6%)	49 (19.1%)	
	bTNBC	27 (84.4%)	181 (80.4%)	208 (80.9%)	

*NMF* Non-negative matrix factorization subtypes, *CALAR* TNBC subtypes described by Kalari group, *Fuzzy* TNBC subtypes described by Jezequel et al., *BLIA* Basal-like immune activated, *BLIS* Basal-like immunosuppressed, *bTNBC* Basal TNBC.

### Molecular profiles of ERβ+ TNBC

**Fig. 1:**
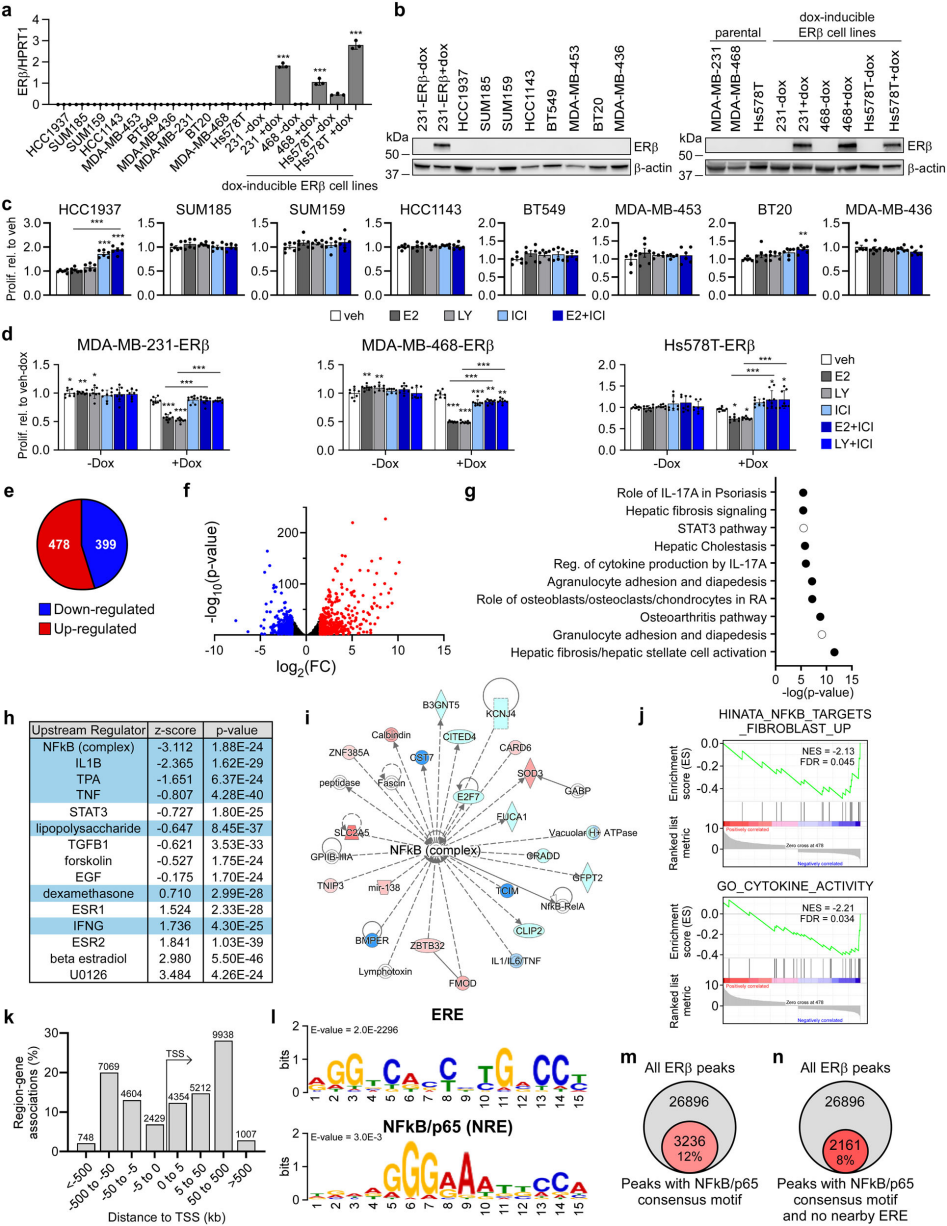
Molecular features of ERβ + TNBC cells. **a** RT-qPCR and (**b**) western blot analysis of ERβ mRNA and protein expression in a panel of TNBC cell lines. Effects of E2, LY, and ICI on proliferation of ERβ− (**c**) and ERβ+ (**d**) TNBC cell lines. **e** Pie graph and (**f**) volcano plot depicting the number and magnitude of gene expression changes detected by RNA-seq of MDA-MB-231-ERβ cells following E2 treatment relative to vehicle (ethanol) control in the presence of dox. IPA analysis of the ERβ transcriptome depicting the (**g**) top 15 canonical pathways regulated by ERβ (NFκB/p65-related pathways shaded black) and (**h**) top 15 upstream regulators (NFκB/p65-associated regulators highlighted in blue). **i** Network analysis of RNA-seq data revealed the NFκB/p65 signaling pathway as centrally down-regulated by ERβ. **j** GSEA of differentially expressed genes identified negative correlations between the ERβ transcriptome and NFκB/inflammatory responses. **k** GREAT and (**l**) motif analysis of ERβ ChIP-seq peaks identified following 3 h of dox + E2 treatment of MDA-MB-231-ERβ cells relative to veh + dox treatment. ERE estrogen response element, NRE NFκB/p65 response element. Venn diagrams of all ERβ binding sites and ERβ binding sites that encode (**m**) an NFκB/p65 consensus motif or (**n**) an NFκB/p65 consensus motif with no ERE within 50 kb. All data is presented as mean ± SEM. *P < 0.05, **P < 0.01, ***P < 0.001 relative to vehicle or between indicated treatments (one-way ANOVA). See also Figure S1.

Given the association of ERβ with tumor suppressive phenotypes in TNBC, we sought to elucidate the molecular consequences of ERβ expression in this disease. Towards this goal, we screened a panel of TNBC cell lines to identify ERβ+ models. Of the 11 cell lines in our panel, none expressed appreciable levels of ERβ mRNA or protein (Fig. 1a, b) and none exhibited alterations in cell proliferation rates when treated with estradiol (E2), the ERβ-specific agonist LY500307 (LY), or the ER antagonist fulvestrant (ICI), with the exception of HCC1937 and BT20 cells, which exhibited increased proliferation in response to ICI (Fig. 1c). Given the lack of TNBC models with endogenous ERβ expression, we utilized cell lines that stably express full length ERβ in a doxycycline (dox)-inducible manner (Fig. 1a, b)^6,12,22^. Expression of ERβ was shown to suppress TNBC cell proliferation following E2 and LY treatment, effects that were completely blocked by ICI in all three models (Fig. 1d).

To elucidate the gene expression changes elicited by ERβ, RNA-sequencing (RNA-seq) was performed on MDA-MB-231-ERβ cells following vehicle and E2 treatment for 5 days (GSE155685). Overall, 877 genes were significantly regulated by ERβ (Fig. 1e, f, Supplemental Table 1). Ingenuity pathway analysis (IPA) identified multiple inflammatory pathways regulated by ERβ, many of which involved NFκB/p65 as a central component (Fig. 1g). Similarly, upstream regulator analysis of the ERβ transcriptome was negatively correlated with multiple ligands known to activate the canonical NFκB signaling pathway (via p65), as well as the NFκB complex itself (Fig. 1h). Network analysis of the ERβ-regulated genes further revealed that the NFκB complex was centrally regulated in this dataset (Fig. 1i). Gene Set Enrichment Analysis (GSEA) showed inverse correlations with several NFκB/inflammatory cytokine pathways (Fig. 1j). Confirmation of ERβ-mediated down-regulation of several p65 target genes was performed via RT-qPCR in an independent set of RNA isolated from MDA-MB-231-ERβ and Hs578T-ERβ cells (Supplemental Fig. 1a, b). Further, we demonstrated that ERβ expression could partially suppress the expression of some p65 target genes even in the absence of a ligand (ie dox vs. no dox treatment) (Supplemental Fig. 1a, b).

We next assessed the genomic distribution of ERβ in MDA-MB-231-ERβ cells using chromatin immunoprecipitation followed by sequencing (ChIP-seq; GSE108981). In total, 26,896 ERβ binding sites were identified following E2 treatment^11^. The majority of ERβ binding sites were located 50–500 kb up- or down-stream of transcriptional start sites, indicating that ERβ primarily functions at distal enhancer regions (Fig. 1k). Motif analysis^23^ identified estrogen response elements (EREs) as the most common ERβ-associated motif, but ERβ was also frequently associated with NFκB/p65 response elements (NREs) throughout the genome (Fig. 1l). In fact, over 12% of all ERβ binding sites contained an NRE (Fig. 1m), most of which did not contain an ERE within 50 kb (Fig. 1n). Example ChIP-seq tracks for ERβ occupancy at p65 target genes found to be repressed by ERβ are shown in Figure S1c following vehicle and E2 treatment.

### Relevance of ERβ-mediated suppression of NFκB/p65 signaling

**Fig. 2:**
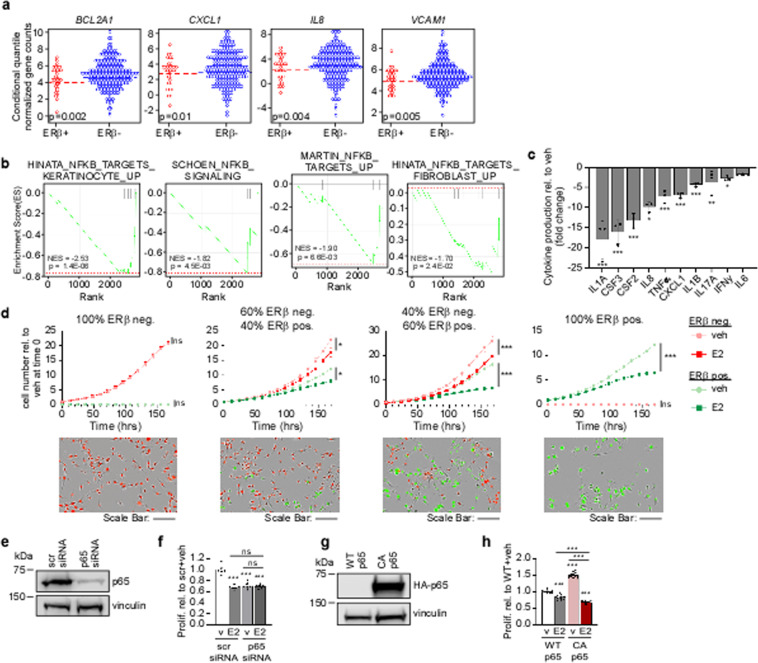
Relevance of NFκB/p65 pathway to ERβ’s tumor suppressive properties. **a** Expression of NFκB/p65 target genes shown to be suppressed by ERβ in TNBC cell lines in ERβ+ (*n* = 32) versus ERβ− (*n* = 225) TN breast tumors from the Mayo Clinic Cohort as assessed by RNA-seq. Dashed line indicates median expression. **b** GSEA of differentially expressed genes in ERβ+ tumors versus ERβ− tumors revealed negative correlations with NFκB/p65 pathways. **c** Protein levels of NFκB/p65 target genes in conditioned medium isolated from MDA-MB-231-ERβ cells treated with E2+ dox for five days relative to vehicle + dox as determined via a cytokine array. **d** Co-culture real-time proliferation assays of parental (red) and ERβ-expressing (green) MDA-MB-231 cells treated with veh + dox or E2+ dox using an IncuCyte S3 system. Representative images of wells are shown in bottom panels. Scale bars are equivalent to 200 µm. **e** Western blot indicating knockdown of p65 protein following transfection of p65-specific siRNAs relative to scramble control (scr). **f** Proliferation rates of dox treated MDA-MB-231-ERβ cells following p65 knockdown and E2 treatment. **g** Western blot depicting expression of the HA-tagged constitutively active p65 expression vector (CA p65) in MDA-MB-231-ERβ cells. **h** Proliferation rates of dox treated parental and CA p65 cells following vehicle and E2 treatment. All data is presented as mean ± SEM. (**c**, **f**, **h**), *P < 0.05, **P < 0.01, ***P < 0.001 relative to vehicle (one-way ANOVA). (**d**) *P < 0.05, **P < 0.01, ***P < 0.001 as determined via two-way ANOVA.

RNA-seq data was available for a sub-set of ERβ+ (*n* = 32) and ERβ− (*n* = 225) patient tumors from the Mayo Clinic TNBC Cohort. Interrogation of these data revealed that the expression of multiple NFκB/p65 target genes were diminished in the ERβ+ tumors relative to ERβ− tumors, including *BCL2A1, CXCL1, IL8 (CXCL8)*, and *VCAM1* (Fig. 2a). GSEA of differentially expressed genes in ERβ+ TN breast tumors revealed significant negative correlations with publically available gene sets related to NFκB signaling (Fig. 2b). To assess whether ERβ-mediated suppression of NFκB/p65 target genes was reflected at the protein level, we performed a Cytokine/Chemokine array (Eve Technologies) using conditioned medium from MDA-MB-231-ERβ cells treated with vehicle or E2. Indeed, the protein levels of multiple NFκB/p65 target genes were significantly decreased in E2 conditioned medium (Fig. 2c). We next performed a co-culture experiment using various ratios of parental MDA-MB-231 cells expressing nuclear red fluorescent protein (RFP) and MDA-MB-231-ERβ cells expressing nuclear green fluorescent protein (GFP) to determine the potential impact of changes in the ERβ regulated secretome on TNBC cell proliferation. As expected, ERβ- parental MDA-MB-231 cells were unaffected by E2 treatment in the absence of ERβ+ cells (Fig. 2d, left). However, the presence of ERβ+ cells resulted in inhibition of ERβ− cell proliferation rates following E2 treatment (Fig. 2d, middle panels), indicating that the alterations in the ERβ regulated secretome are of relevance to the anti-proliferative effects of ERβ-targeted therapies (Fig. 2d).

To further assess the importance of changes in canonical NFκB signaling with regard to the anti-cancer effects of ERβ, we determined the impact of p65 knockdown (Fig. 2e, f) and constitutive activation (Fig. 2g, h) on ERβ+ cell proliferation. siRNA-mediated knockdown of p65 in MDA-MB-231-ERβ cells significantly inhibited proliferation to an extent that was identical to E2 treatment (Fig. 2f). Further, the inhibitory effects of E2 were lost in the setting of p65 knockdown (Fig. 2f). Expression of a constitutively active form of NFκB/p65 (CA p65) in MDA-MB-231-ERβ cells (Fig. 2g) resulted in increased cell proliferation (Fig. 2h). Intriguingly, E2 was a more potent inhibitor of proliferation in cells with constitutively active p65 (Fig. 2h). Taken together, these data suggest that inhibition of p65 signaling is a primary mechanism by which ERβ elicits anti-cancer effects in TNBC cells.

### ERβ blocks ligand-mediated activation of canonical NFκB/p65 signaling

**Fig. 3:**
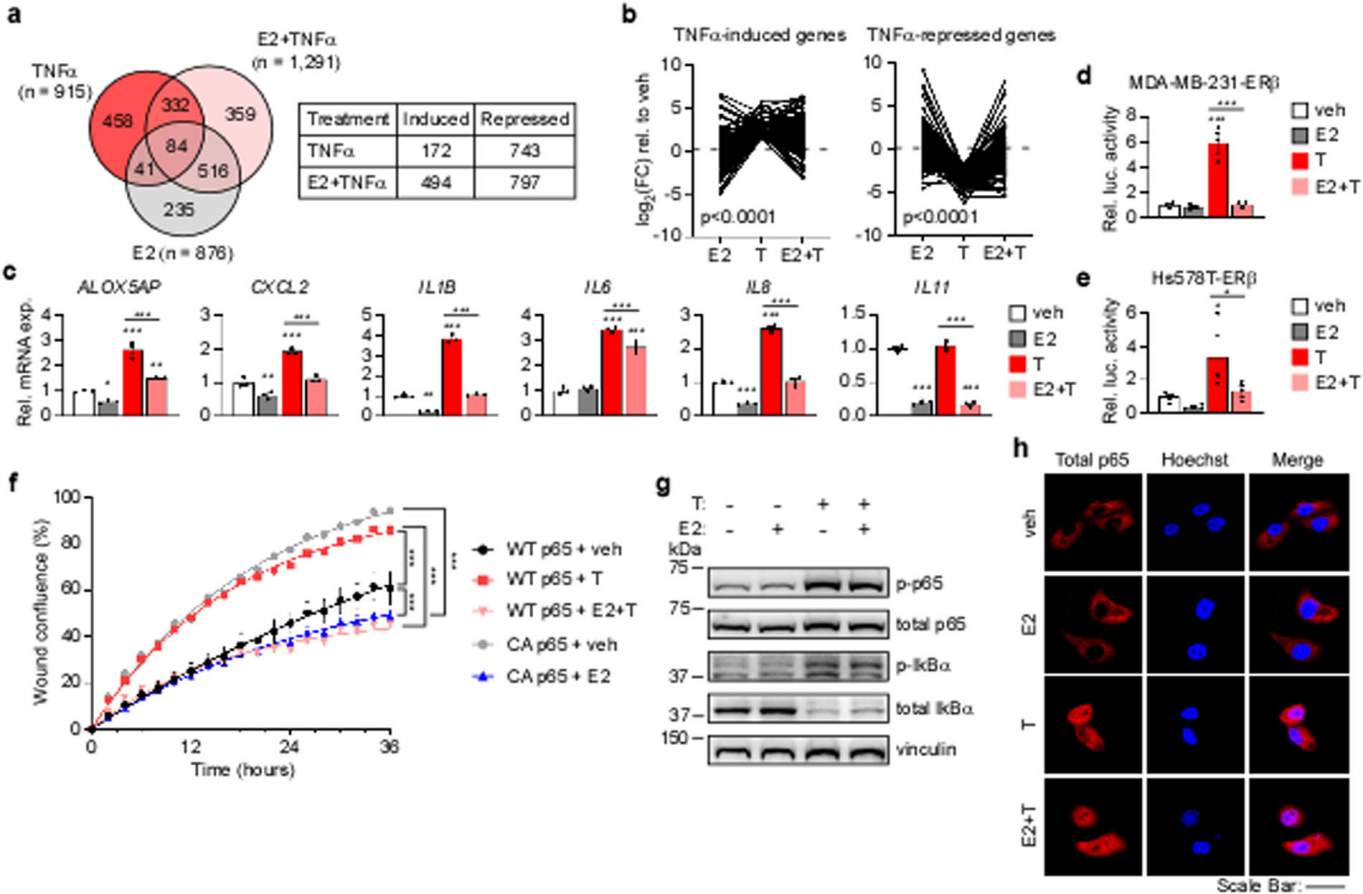
ERβ blocks ligand-mediated activation of canonical NFκB/p65 signaling and cell migration. **a** Venn diagram depicting the overlap of genes differentially expressed in dox treated MDA-MB-231-ERβ cells following TNFα, E2+ TNFα, or E2 stimulation relative to vehicle control. The number of induced and repressed genes by each treatment is indicated. **b** Fold change (FC) relative to vehicle of TNFα-induced and -repressed genes following E2, TNFα (T), or E2+ TNFα (E2+ T) treatment (Wilcoxon rank sum test). **c** RT-qPCR analysis of NFκB/p65 target gene mRNA expression relative to vehicle in MDA-MB-231-ERβ cells treated as indicated in the presence of dox. NFκB/p65 luciferase reporter activity relative to vehicle in (**d**) MDA-MB-231-ERβ and (**e**) Hs578T-ERβ cells following indicated treatments in the setting of dox. **f** Migration assay of dox treated MDA-MB-231-ERβ cells and constitutively active p65 cells (CA p65) following veh, T, E2+ T, or E2 treatment. ***P < 0.001 between indicated treatments (non-linear fit modeling). **g** Western blot analysis of phospho- and total p65 and IκBα following indicated treatments in the presence of dox. Vinculin is shown as a loading control. **h** Confocal microscopy images depicting cellular localization of total p65 in MDA-MB-231-ERβ cells following indicated treatments in addition to dox. Hoechst stain used to identify nuclei. Scale bars are equivalent to 50 µm. Data represent mean ± SEM. **c**–**e** *P < 0.05, **P < 0.01, ***P < 0.001 relative to vehicle or between indicated treatments (one-way ANOVA). See also Figure S2.

Based on our findings that ERβ repressed basal p65 signaling in TNBC, we next investigated whether ERβ could block ligand-mediated activation of the canonical NFκB/p65 signaling pathway. We chose to activate canonical NFκB/p65 signaling with tumor necrosis factor alpha (TNFα), a potent ligand for canonical NFκB/p65 signaling known to promote proliferation, migration, and primary tumor growth of TNBC^24,25^. TNFα was also chosen since it was identified as a top-upstream regulator in our IPA analysis of the ERβ transcriptome (Fig. 1d). RNA-seq analysis identified 915 genes regulated by TNFα in MDA-MB-231-ERβ cells (Fig. 3a, Supplemental Table 2). TNFα failed to significantly regulate 458 of these genes (50%) when E2 was present (E2+ TNFα treatment) (Fig. 3a, Supplemental Table 3). Indeed, E2 treatment largely blocked or attenuated the ability of TNFα to induce or repress NFκB/p65 target genes (Fig. 3b). Raw sequencing data is available in GEO under accession number GSE155685. These findings were confirmed via RT-qPCR in MDA-MB-231-ERβ cells (Fig. 3c) and in Hs578T-ERβ cells (Supplemental Fig. 2a). As with the basal gene expression levels, treatment with doxycycline (dox) alone (i.e., expression of ERβ alone) also diminished the ability of TNFα to activate p65 target genes (Supplemental Fig. 2b). In addition, ERβ also prevented TNFα-mediated activation of a NFκB/p65 luciferase reporter construct in ERβ+ TNBC cells (Fig. 3d, e). Finally, TNFα induced migration of MDA-MB-231-ERβ cells similar to that of cells expressing a constitutively active form of NFκB (Fig. 3f). E2 blocked TNFα-induced migration and potently suppressed migration of the CA p65 cell line (Fig. 3f).

To gain insight into the mechanisms by which ERβ suppresses p65 signaling, we determined the impact of ERβ on phosphorylation of p65 (RELA) and its upstream inhibitor, IκBα. As expected, TNFα induced phosphorylation of both p65 and IκBα and induced loss of total IκBα in TNBC cells (Fig. 3g). However, E2 treatment had no impact on phosphorylation of p65 or IκBα alone or in the presence of TNFα (Fig. 3g), nor did E2 treatment of ERβ expressing cells affect p65 nuclear localization following TNFα stimulation (Fig. 3h).

### ERβ interacts with p65 and alters its genomic localization

**Fig. 4:**
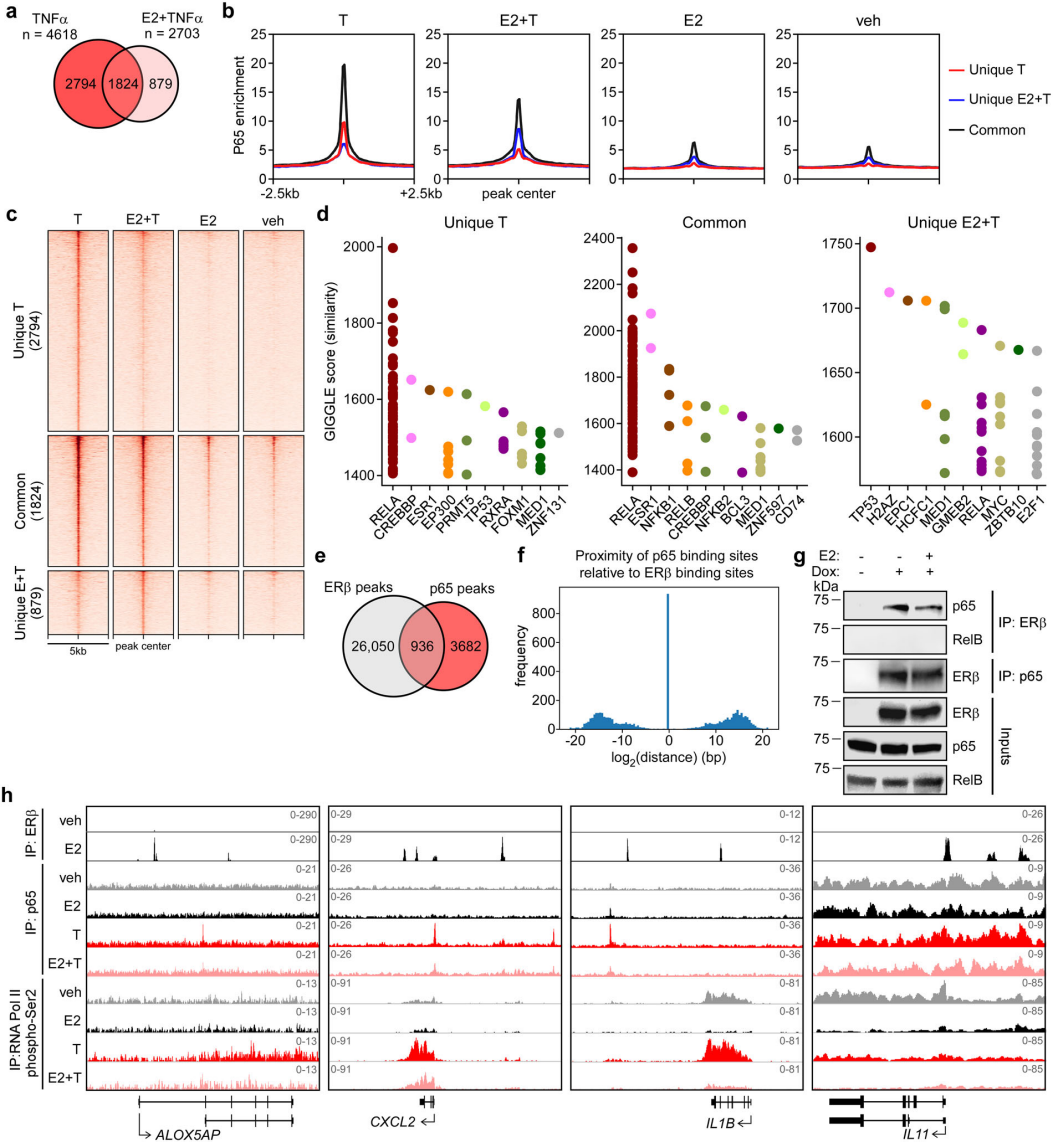
ERβ interacts with p65 and alters its genomic localization. **a** Venn diagram depicting overlap of p65 binding sites following TNFα or E2+ TNFα stimulation in dox treated MDA-MB-231-ERβ cells as determined by ChIP-seq. **b** Aggregate plots and (**c**) heat maps of p65 binding intensity in the presence of TNFα alone (Unique T), E2+ TNFα (Unique E + T), or both (Common) following indicated treatments. **d** GIGGLE analysis of identified p65 binding sites assessing their similarity with other known protein binding sites in publicly available datasets. **e** Venn diagram depicting overlap of ERβ and p65 binding sites identified via ChIP-seq. **f** Bar graph of all p65 binding sites and their distribution relative to ERβ binding sites. Note the 936 overlapping sites from (**e**) are at the exact same genomic location. **g** Co-immunoprecipitation experiments using nuclear lysates from dox treated MDA-MB-231-ERβ cells demonstrating protein interactions between ERβ and p65, but not RELB. **h** ChIP-seq tracks from IGV for ERβ, p65, and RNA Polymerase II phospho-Ser2 ChIP-seq following dox plus veh, E2, T, and E2+ T treatment at NFκB/p65 target gene loci. See also Figure S3.

Given that ERβ did not alter phosphorylation or nuclear localization of p65, we hypothesized that ERβ suppresses p65 transcriptional activity by altering its chromatin binding profiles. To address this possibility, ChIP-seq was used to elucidate p65 chromatin binding sites following TNFα stimulation in the absence and presence of E2 (GSE155684). A total of 4618 and 2703 peaks were identified for p65 in MDA-MB-231-ERβ cells following TNFα treatment alone or in combination with E2, respectively (Fig. 4a). Approximately 60% of p65 binding sites identified following TNFα stimulation (2794 out of 4618) were diminished in the presence of E2, while 40% (1824 of 4,618) were maintained (Fig. 4a). p65 binding was strongest at the 1824 common sites regardless of treatment (Fig. 4b, c). p65 primarily localized to intergenic and intronic regions of chromatin, but shifted to promoter regions at sites bound by p65 only in the presence of E2 plus TNFα (Supplemental Fig. 3a). GIGGLE analysis^26^ revealed that p65 occupancy was most strongly associated with known RELA (NFκB/p65) binding sites for peaks identified in the TNFα unique and common groups, as well as with ER (ESR1) binding sites (Fig. 4d). In comparison, the most common motif bound by p65 in the presence of E2+ TNFα was a p53 binding site while RELA motifs were much less frequent and ER motifs were rarely identified (Fig. 4d). Comparison of the p65 peaks identified following TNFα stimulation with ERβ peaks following E2 treatment revealed that 20.2% of all canonical p65 binding sites (936 of 4618) were in the exact same genomic location as an ERβ binding site, with other p65 binding sites evenly distributed up- and down-stream of ERβ peaks (Fig. 4e, f). Using ChIP-PCR for ERβ in MDA-MB-231-ERβ cells following vehicle, E2, TNFα or E2+ TNFα treatment, we confirmed that ERβ is capable of associating with chromatin at regions encoding p65 binding sites in response to both estrogen and TNFα (Supplemental Fig. 3b).

We next sought to determine if ERβ and p65 physically interacted. Co-immunoprecipitation assays using MDA-MB-231-ERβ cells demonstrated that ERβ and p65 interact at the protein level in an E2-independent manner (Fig. 4g). However, ERβ did not interact with RELB (Fig. 4g), a component of non-canonical NFκB signaling. These observations were confirmed in 293 T cells (Supplemental Fig. 3c). Representative ChIP-seq tracks for ERβ and p65 at two NFκB/p65 target genes with overlapping peaks (*CXCL2* and *IL11*) and adjacent peaks (*ALOX5AP* and *IL1B*) are shown in Fig. 4h. ChIP-seq tracks for RNA Pol II phospho-Ser2 (GSE155684) are also shown and corroborate the gene expression studies demonstrating that E2 treatment suppresses basal transcription of these genes and blocks their induction by TNFα (Fig. 4h).

### ERβ assembles a co-repressor complex with EZH2 to inhibit p65 transcriptional activity

**Fig. 5:**
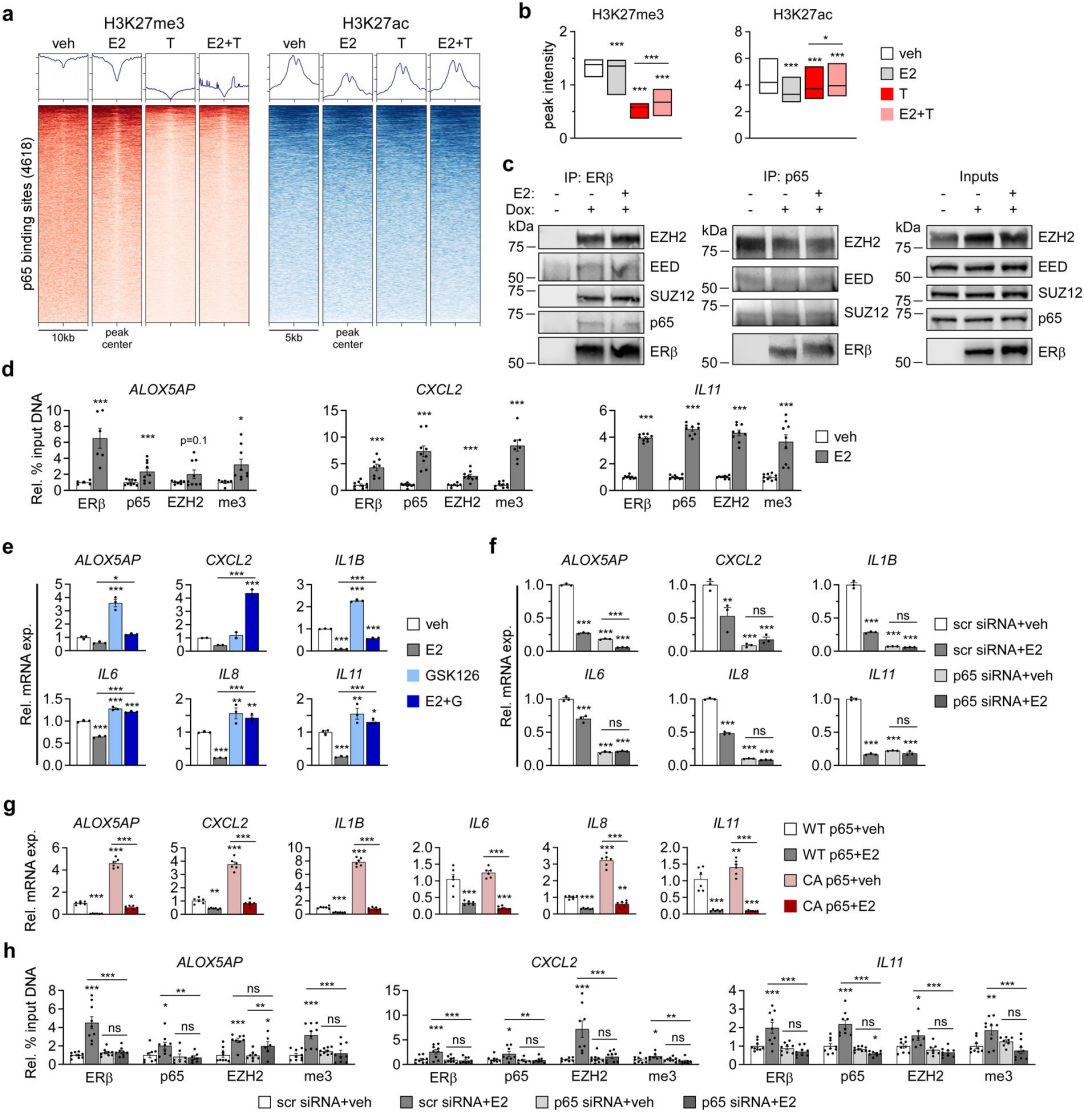
ERβ assembles a co-repressor complex to inhibit p65 transcriptional activity. **a** Heat maps, aggregate plots, and (**b**) box plots of H3K27me3 and H3K27ac peak intensity at TNFα-induced p65 binding sites in dox treated MDA-MB-231-ERβ cells following vehicle (veh), E2, TNFα (T) or E2+ TNFα (E2+ T) stimulation. *P < 0.05, **P < 0.01, ***P < 0.001 relative to vehicle or between indicated treatments (Mann–Whitney test). **c** Co-immunoprecipitation experiments using nuclear lysates from MDA-MB-231-ERβ cells treated with or without dox and veh or E2. Lysates were immunoprecipitated with an ERβ antibody or a p65 antibody followed by western blotting for indicated proteins. **d** ChIP-PCR depicting the relative binding of ERβ, p65, and EZH2, as well as enrichment of H3K27me3 (me3), at ERβ binding sites nearby NFκB/p65 target genes (*ALOX5AP*, *CXCL2, and IL11)* following dox + vehicle or dox + E2 treatment. RT-qPCR analysis of NFκB/p65 target gene expression in dox treated MDA-MB-231-ERβ cells exposure to: (**e**) veh, E2, 5 µM GSK126, or E2 + GSK126 (E2 + G), or (**f**) a non-targeting siRNA (scr) or p65-targeting siRNA in the presence of veh or E2. **g** RT-qPCR analysis of NFκB/p65 target gene expression in WT and CA p65 MDA-MB-231-ERβ cells treated with veh or E2 relative to WT cells + vehicle and dox. **h** ChIP-PCR assessing relative association of ERβ and EZH2, as well as H3K27me3 (me3), at ERβ binding sites near NFκB/p65 target genes following treatment with non-targeting siRNA (scr) or p65-targeting siRNA in the presence of dox + vehicle or dox + E2. All data is presented as mean ± SEM. except for (**b**) which depicts the minimum, maximum, and median values. *P < 0.05, **P < 0.01, ***P < 0.001 relative to vehicle or between indicated treatments. See also Figure S4.

Since ERβ did not completely block p65 association with chromatin and was found to be co-localized at many NFκB/p65 binding sites throughout the genome, we next assessed the impact of ERβ on chromatin architecture at these genomic loci. ChIP-seq tracks for H3K27me3 and H3K27ac (GSE155684) at representative NFκB/p65 target genes are shown in Supplemental Fig. 4a. At NFκB/p65 binding sites, TNFα treatment resulted in a substantial decrease in the transcriptionally repressive H3K27me3 mark with minimal changes to the transcriptionally active H3K27ac mark (Fig. 5a, b). However, E2 treatment in combination with TNFα significantly increased H3K27me3 relative to TNFα alone (Fig. 5a, b). Given that EZH2 is the catalytic component of the PRC2 responsible for trimethylation of H3K27, we determined if ERβ interacts with EZH2 and other members of the complex. Co-immunoprecipitation studies using MDA-MB-231-ERβ nuclear extracts revealed that ERβ associates with EZH2, EED, and SUZ12 (Fig. 5c). p65 was also shown to be a component of this repressive complex (Fig. 5c). ChIP-qPCR at known ERβ binding sites near specific NFκB/p65 target genes demonstrated increased ERβ binding in response to E2 treatment, which also resulted in recruitment of p65 and EZH2 and was associated with increased H3K27me3 (me3) (Fig. 5d). Considering enrichment of H3K27me3 and recruitment of EZH2 to ERβ binding sites in close proximity to NFκB/p65 target genes, we examined the impact of drug-mediated inhibition of EZH2 on NFκB/p65 target gene expression (Fig. 5e, Supplemental Fig. 4b). Treatment of MDA-MB-231-ERβ cells with GSK126, an inhibitor of EZH2 catalytic activity, resulted in significant up-regulation of five out of the 6 p65 target genes examined (Fig. 5e). In the presence of GSK126, the effects of E2 were attenuated and the ability of ERβ to suppress the expression of these genes below basal expression levels was lost with the exception of IL1B (Fig. 5e). Similar results were obtained in the Hs578T-ERβ cell line (Supplemental Fig. 4b).

### p65 is required for function of the ERβ/EZH2 co-repressor complex

Since p65 was shown to be a component of the ERβ/EZH2 repressive complex and was recruited to ERβ-bound sites near NFκB/p65 target genes, we determined the necessity of p65 for ERβ-mediated gene silencing. As expected, siRNA-mediated knockdown of p65 reduced basal expression of NFκB/p65 target genes relative to control siRNA transfected cells (Fig. 5f). However, the inhibitory effects of E2 on the expression levels of these genes were diminished or completely lost in the setting of p65 knockdown (Fig. 5f). Conversely, NFκB/p65 target gene expression was induced in cells expressing constitutively active p65 (Fig. 5g), and E2 potently suppressed their expression (Fig. 5g). When p65 was silenced, recruitment of ERβ, p65, and EZH2 to known ERβ binding sites near NFκB/p65 target genes in response to E2 was lost (Fig. 5h). This coincided with a loss of H3K27me3 at these loci following E2 treatment (Fig. 5h).

## Discussion

Treatment options for patients with TNBC are limited, and identifying new biomarkers and therapeutic targets for this disease remains critical for improving outcomes. We assessed ERβ protein expression in the largest reported TNBC cohort using a well-validated monoclonal antibody^16–18^, and found that ERβ was expressed in 18% of TN breast tumors. Patients with ERβ+ tumors were more likely to present with decreased clinical stage (smaller tumors and no nodal involvement). Like ERα expression^27^, ERβ positivity was associated with significantly decreased stromal TILs, although the composition and frequency of specific types of lymphocytes in ERβ+ versus ERβ− tumors remains to be elucidated. Interestingly, NFκB/p65 is an essential player in the recruitment of TILs to the tumor stroma^28,29^. Thus, ERβ-mediated suppression of p65 signaling may be in part responsible for the decrease in TILs in ERβ+ tumors. The proportion of AR positivity among the ERβ+ and ERβ− tumors was nearly identical in this TNBC cohort, demonstrating that these two important hormone receptors are not mutually inclusive or exclusive in this form of the disease. ERβ+ and ERβ− tumors were also similarly distributed across the known subtypes of TNBC. Interestingly, in pre-menopausal women with ERβ+ tumors, OS was found to be significantly worse than pre-menopausal women with ERβ− tumors. This association was not seen in post-menopausal patients. There could be multiple reasons for these findings including the fact that aggressive chemotherapy regimens were less frequently administered to women with ERβ+ disease. It is also possible that chemotherapy responsiveness may be diminished in ERβ+ tumors as is known to be the case for ERα+ disease^30^. Further, the fact that ERβ+ tumors were less likely to have high levels of stromal TILs may have contributed to these findings given that high numbers of TILs are associated with improved outcomes in TNBC patients as well as increased chemotherapy responsiveness^15^. Finally, it is possible that ERβ+ tumors that develop in young pre-menopausal women represent a different entity of disease compared to ERβ+ tumors that develop in older post-menopausal patients. These possibilities warrant further study in additional patient cohorts and highlight the need to include menopausal status as a stratification factor when assessing ERβ associations with tumor characteristics and patient outcomes.

The uses of tamoxifen and aromatase inhibitors for chemoprevention of breast cancer are known to successfully reduce the incidence of ERα+ breast cancer but not TNBC^31–34^. However, these treatment interventions fail to reduce breast cancer mortality^31–34^. In contrast, long term follow-up results from the randomized, placebo-controlled Women’s Health Initiative (WHI) trials in post-menopausal women showed that the use of estrogen alone as a menopausal hormone therapy decreased breast cancer incidence and death for patients with both ERα+ disease and TNBC^13,14^. Additionally, there were reductions in lymph node positive disease for estrogen treated women^13^, which parallel our findings for ERβ. Finally, E2 has been shown to improve OS of breast cancer patients when compared to tamoxifen^35,36^. While the basis for these observations has yet to be elucidated, further studies evaluating the role of ERβ in these settings is warranted.

Uncovering the mechanisms through which ERβ functions is critical towards the goal of developing it as a useful prognostic biomarker and drug target. Here, we provide evidence that ERβ+ TN breast tumors have decreased p65 pathway activity and demonstrate that ERβ directly suppresses p65 transcriptional activity in TNBC cells. We also provide evidence that suppression of p65 signaling is a primary mechanism by which ERβ elicits its anti-cancer effects. Importantly, we found through co-culture studies that ligand mediated activation of ERβ resulted in alterations in cell-to-cell communication that led to decreased proliferation of non-ERβ expressing TNBC cells, potentially as a result of decreased levels of p65 regulated cytokines. These findings indicate that ERβ targeted therapies may still elicit anti-cancer effects in tumors with heterogenous expression of ERβ, a possibility that will need to be confirmed in cell line and PDX tumor models as well as clinical trials.

Canonical NFκB signaling via p65/RELA is known to promote carcinogenesis, enhance tumor cell survival and tumor progression, promote the development of metastatic disease, and induce resistance to standard of care chemotherapy regimens in TNBC^37–42^. In addition, decreased expression of NFκB/p65 target genes is associated with improved outcomes for TNBC patients^43^ and decreased invasiveness of TNBC cell lines^44^. Finally, suppression of *IL6* and *IL8*, genes shown to be repressed by ERβ in the present study, have also been shown to potently inhibit proliferation, migration, and tumor formation of TNBC cells^45,46^. Although drugs specifically targeting the canonical NFκB/p65 pathway elicit potent anti-cancer effects in vitro, they have thus far failed in the clinic due to off-target effects, immune suppression-related issues, and substantial toxicity^47^. Our data suggest that ligand-mediated activation of ERβ is an effective way to block canonical NFκB/p65 signaling and invoke potent anti-cancer effects in TNBC.

Previous studies have shown that ERβ can suppress canonical NFκB/p65 signaling in various cell and tissue types, however the mechanism(s) remains unclear and these effects have not been studied in TNBC^48–52^. Mechanistically, we provide evidence that ERβ does not alter the upstream signaling pathways required for p65 activation, nor does it impact p65 phosphorylation or nuclear localization. Instead, we show that ERβ modifies the genomic distribution of p65 in TNBC cells. At some genomic loci, ERβ partially or completely, displaced p65 association with chromatin in the setting of E2 treatment. Indeed, a substantial proportion (20%) of all p65 binding sites directly overlapped with an identified ERβ binding site. However, an equal number of p65 binding sites were identified that were either maintained or gained in the presence of E2, suggesting that ERβ alters p65 transcriptional activity via multiple mechanisms that extend beyond simply displacing p65 from chromatin. Interestingly, in the setting of p65 and ERβ activation (ie TNFα+ E2 treatment), p53 motifs represented the most enriched binding site for p65. p53 and p65 are known to interact with one another and oppose each other’s activity^53^. Since ERβ has also been reported to interact with p53^54^, it is possible that estrogen treatment of ERβ+ TNBC cells drives the formation of a complex consisting of p65 and p53 to further dampen NFκB/p65 signaling. Indeed, we confirmed an interaction between ERβ and p65 using co-immunoprecipitation and proximity-based ligation assays. Further, we showed that E2 is a more potent inhibitor of proliferation and migration in TNBC cells expressing a constitutively active form of p65, suggesting that p65 plays an important role in mediating the anti-cancer effects of ERβ and indicating that ERβ-targeted therapies may be most effective in tumors with high NFκB/p65 activity.

To further understand the mechanisms by which ERβ suppresses p65 transcriptional activity, we assessed histone modifications and chromatin architecture at p65 binding sites. Our studies revealed that E2 treatment, both in the presence and absence of TNFα stimulation, significantly increased H3K27me3, a histone mark associated with gene silencing^55^. Trimethylation of H3K27 primarily occurs through the catalytic activity of EZH2 as a part of the PRC2 complex^56^. Previous studies have shown that ERβ interacts with the PRC2 complex^57^. We demonstrate that the methyltransferase activity of EZH2 is critical for ERβ-mediated silencing of NFκB/p65 target genes. Intriguingly, knockdown of p65prevented ERβ-mediated growth suppression of TNBC cells and diminished ERβ’s ability to repress NFκB/p65 target gene expression. Knockdown of p65 also disrupted association of ERβ, EZH2, and H3K27me3 at ERβ binding sites near NFκB/p65 target genes, further confirming that these three proteins participate in the formation of a co-repressor complex that has not been previous described.

In contrast with our findings, previous studies have found that EZH2 activates gene expression in breast and prostate tumors^58–61^, and functions as an oncogene by promoting canonical NFκB/p65 signaling in TNBC^45,62^. However, it is important to note that these previously described “co-activator” properties of EZH2 occur through methyltransferase- and PRC2-independent mechanisms^62^. EZH2 overexpression in TNBC is known to enhance tumor progression and metastasis and is associated with advanced tumor stage and increased mortality^63–66^. Paradoxically, H3K27me3 is associated with improved breast cancer patient outcomes^64,67^. These findings suggest that in TNBC, the histone methyltransferase activity of EZH2 is usurped for non-canonical oncogenic functions, including the activation of NFκB signaling^62^. Here, we demonstrate that in the absence of ERβ expression and activity, these functions of EZH2 occur, given that canonical NFκB/p65 signaling is high and H3K27me3 near p65 binding sites is low. However, upon expression and activation of ERβ, EZH2 is recruited to deposit H3K27me3 near NFκB/p65 target genes, resulting in their transcriptional repression. These findings are clinically relevant given that EZH2 inhibitors are currently being tested in clinical trials for breast cancer^68–70^. Our results suggest that the use of such drugs in patients with ERβ+ disease may actually be detrimental, a possibility that needs to be thoroughly examined in future studies.

**Fig. 6:**
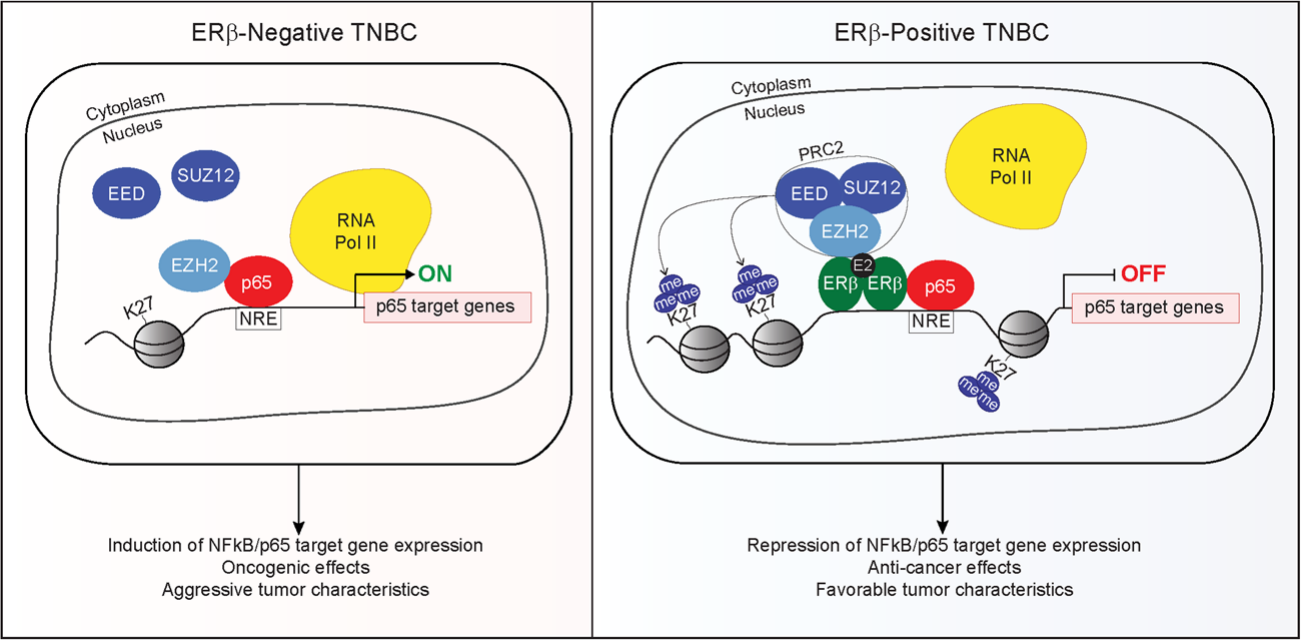
Model. In ERβ− TNBC cells (left), EZH2 associates with p65 in a PRC2-independent manner and functions to enhance p65 transcriptional activity and promote aggressive cancer phenotypes. In ERβ+ TNBC cells (right), ERβ induces formation of a co-repressor complex involving p65, EZH2, and the PRC2 complex (including SUZ12, EED, and EZH2). This repressive complex results in trimethylation of H3K27, chromatin compaction, and subsequent blockade of NFκB/p65 target gene expression, ultimately resulting in anti-cancer effects and less aggressive cancer phenotypes.

In summary, we have found that ERβ expression is associated with favorable prognostic features, but paradoxically is associated with worse OS in pre-menopausal, but not post-menopausal patients. Mechanistically, we report that ERβ assembles a co-repressor complex consisting of EZH2, other PRC2 components, and p65 to suppress p65 transcriptional activity through chromatin silencing at NFκB/p65 target gene loci, ultimately resulting in inhibition of TNBC growth and migration (Fig. 6). Additionally, our results indicate that ERβ can function as a molecular switch for EZH2 and repurpose it for anti-cancer effects, findings that may help clarify the paradox between EZH2, H3K27me3, and TNBC patient outcomes. Our findings also demonstrate that ERβ represents a therapeutic target for a proportion of TNBC patients, a possibility that is being tested through an ongoing phase II clinical trial (NCT03941730). Since NFκB/p65 can promote resistance to standard-of-care chemotherapies^38–42^, ERβ-targeted therapies may also enhance chemotherapy responsiveness in a subset of patients. Overall, identification of ERβ+ TN breast tumors, in combination with the use of ERβ-targeted therapies, has the potential to improve the outcomes of a proportion of TNBC patients for whom treatment options are currently limited.

## Methods

### Study cohort and immunohistochemistry

The Mayo Clinic TNBC Cohort^15^ was used for this study. Immunohistochemistry (IHC) analysis for ERβ expression was completed using the PPG5/10 monoclonal antibody (BioRad Laboratories, Inc., Hercules, CA, USA) and the CAP/CLIA-based protocol^16^ (Immunostains Laboratory, Mayo Clinic, Rochester, MN, USA). Stromal TILs were assessed by counting all mononuclear cells in the stromal compartment that were found within the borders of the invasive tumor. Stromal TILs are reported as the percentage of total cells in the same area.

### Materials and reagents

17β-estradiol (E2), doxycycline (dox), and Tumor Necrosis Factor alpha (TNFα) were purchased from Sigma-Aldrich (St. Louis, MO, USA). LY500307 (LY) was provided by Eli Lilly (Indianapolis, IN, USA). ICI182,780 (ICI) was purchased from Tocris Bioscience (Bristol, United Kingdom). GSK126 was purchased from Selleckchem (Houston, TX, USA).

### Cell culture

HCC1937, HCC1143, MDA-MB-453, MDA-MB-436, BT549, MDA-MB-231, BT20, MDA-MB-468, and Hs578T cells were obtained from ATCC. SUM185 and SUM159 cells were purchased from BioIVT (Westbury, NY, USA). All parental cell lines were maintained in phenol red-free DMEM/F12 medium supplemented with 10% fetal bovine serum (FBS) and 1% antibiotic/antimycotic (AA). MDA-MB-231, MDA-MB-468, and Hs578T cells were engineered to express full-length human ERβ in a doxycycline (dox)-inducible manner (MDA-MB-231-ERβ) as previously described^6,12,22^. Dox-inducible cells were maintained in the same medium supplemented with 5 mg/L blasticidin S and 500 mg/L zeocin. Parental MDA-MB-231 and MDA-MB-231-ERβ cells were developed to stably express nuclear red and green fluorescent protein, respectively, following infection with NucLight Red Lentivirus (#4625, Sartorius Stedim Biotech, Göttingen, Germany) and NucLight Green Lentivirus (#4624, Sartorius), at a multiplicity of infection (MOI) of 5. Following 72 h of infection, virus-containing medium was removed and replaced with normal medium containing 500 µg/L puromycin for selection. MDA-MB-231-ERβ cells stably expressing a constitutively active form of NFκB (S276G) were generated by transfection of the HA-tagged pCMV3 NFκB p65 expression vector (Sino Biological, Beijing, China) after point mutation generation via Quikchange® PCR. A clonal cell line was generated following selection with 50 µg/ml hygromycin B. Experiments utilizing ERβ ligands were performed in growth medium containing 10% triple-charcoal/dextran-stripped FBS (HyClone™, GE Healthcare Life Sciences, Pittsburg, PA, USA). All cell lines were routinely checked for mycoplasma contamination and were authenticated via IDEXX BioAnalytics (Columbia, MO).

### Real-time qPCR

Cells were seeded in 12-well plates in medium containing 100 ng/ml doxycycline (dox) to induce ERβ expression and, if necessary, treated with ethanol vehicle or 1 nM E2 for 5 days, with a media change on day 3. After 5 days, 20 ng/mL of TNFα was added for 24 h. RNA was isolated using TRIzol™ Reagent (Thermo Fisher Scientific, Waltham, MA, USA) and cDNA was generated from 1 µg of total RNA using the iScript™ cDNA Synthesis Kit (Bio-Rad). Real-time quantitative PCR (RT-qPCR) was conducted using PerfeCTa™ SYBR Green Fast Mix™ (Quanta Biosciences, Gaithersburg, MD, USA) and a Bio-Rad CFX Real-Time PCR detection system (Bio-Rad). RT-qPCR primer sequences are listed in Supplemental Table 4.

### Western blotting

Cells were seeded in 6-well plates and treated as described above with the exception that TNFα treatments persisted for 10 min. Cell lysates were harvested using NETN buffer (150 mM NaCl, 1 mM EDTA, 20 mM pH 8.0 Tris, 0.5% NP-40) containing 1X cOmplete™ Protease Inhibitor Cocktail without EDTA (PI) (Sigma-Aldrich) and 1X PhosSTOP™ phosphatase inhibitor (Sigma-Aldrich). Protein concentrations were determined using the Pierce™ BCA Protein Assay Kit (Thermo Fisher Scientific) and a SpectraMax Microplate Reader (Molecular Devices, San Jose, CA, USA). Equal amounts of protein were separated on 7.5% SDS polyacrylamide gels, transferred to PVDF membranes which were subsequently blocked for 1 h at room temperature using 5% milk or 5% BSA in 1X TBST depending on primary antibody. Membranes were incubated with primary antibody overnight at 4 °C, washed with 1X TBST, incubated with secondary antibody for 1 h at room temperature, and washed again. Blots were imaged on the Odyssey Fc (LI-COR, Lincoln, NE, USA) system using the chemiluminescent and 700 nm channels for 10 min and 30 sec, respectively. All blots for a given experiment were derived from the same experiment and were processed in parallel. Uncropped and unprocessed scans of all blots presented in this manuscript are provided in the Supplementary Information document. Antibody information can be found in Supplemental Table 5.

### Proliferation assays

Cells were seeded in 96-well plates in replicates of six and treated with or without dox for 24 h prior to indicated treatments. Following treatment, cells were allowed to proliferate for seven days, at which point they were fixed with 25% (v/v) glutaraldehyde (Sigma-Aldrich) for 10 min, washed four times with water, stained with crystal violet, and washed again. Crystal violet was solubilized with 100 µl of a solution containing 100 nM sodium citrate and 50% ethanol and quantitated using a plate reader at 550 nm excitation.

Co-culture proliferation assays were performed in the same manner with various proportions of ERβ+ and ERβ− cells seeded in the same wells for a total of 1000 cells/well at the start of the assay. Proliferation rates were monitored in real-time for the two populations by tracking the number of red and green nuclei using an IncuCyte® S3 system (Sartorius).

### RNA-seq

MDA-MB-231-ERβ cells were plated in triplicate in 10 cm dishes in the presence of dox. Cells were treated with vehicle or 1 nM E2 for a total of 5 days with one media change and treatment refresh on day 3. For TNFα treatments, 20 ng/ml TNFα was added for the final 3 h of treatment. Total RNA was isolated using TRIzol™ Reagent and a miRNeasy Mini kit (Qiagen, Hilden, Germany) following the manufacturer’s protocol. Total RNA was submitted to the Mayo Clinic Genome Analysis Core for sequencing (Rochester, MN). RNA libraries were prepared using 200 ng of total RNA and the TruSeq Stranded mRNA Sample Prep Kit (Illumina, San Diego, CA, USA) according to the manufacturer’s instructions. Fifty base-pair paired-end reads were generated using an Illumina HiSeq 4000 sequencer and software (HD 3.4.0.38) with approximately 50 million fragment reads per sample. Base-calling was performed using Illumina’s RTA version 2.7.7. Library preparation and primary analysis was performed by the Mayo Clinic Medical Genome Facility Genome Analysis Core. Mapped reads were assigned using featureCounts and batch effect correction was performed with RUVseq using a curated set of housekeeping genes to normalize the batches. An RPKM cutoff was used to remove lowly expressed genes (RPKM < 1) from further analyses. Comparison tests were performed using edgeR and significance was measured using |log_2_(fold change)| ≥ 1.5, *p* < 0.05, and FDR ≤ 0.1^71,72^.

### RNA-seq of TN breast tumors

RNA-Sequencing data was generated for 301 formalin-fixed paraffin-embedded (FFPE) tissues from the Mayo Clinic TNBC Cohort^15^. The raw sequencing files from RNA-seq were processed through the Mayo Analysis Pipeline for RNA-Seq (MAP-RSeq)^73^. After applying quality control filters, 269 samples remained with high quality gene expression data for further analysis. Gene expression was normalized using conditional quantile normalization (CQN)^74^ to account for gene length and library size. Differential expression analysis was performed using the edgeR package^75^ by modeling the raw gene counts predicted by ERβ status with a negative binomial model, taking into account subject and gene-specific dispersion, which were estimated in the CQN method. Differential expression results are reported as log2 fold-change between groups with false discovery rate (FDR) *p* values reported for multiple testing adjustments.

### Biological pathway and gene set enrichment analysis

Differentially expressed genes were utilized for canonical pathway and upstream regulator identification with Ingenuity Pathway Analysis software (IPA, Ingenuity Systems, Inc., Redwood City, CA, USA; http://www.ingenuity.com). Associations of gene signatures derived from RNAseq data using MDA-MB-231-ERβ cell lines and human tumors with publically available databases were performed using Gene Set Enrichment Analysis (GSEA)^76,77^.

### Cytokine/chemokine array

MDA-MB-231-ERβ cells were plated in triplicate in six-well plates in dox-containing medium and were treated with ethanol vehicle or 1 nM E2 for five days with a media change on day 2. On day 4, cells were washed with 1X PBS and media was changed to serum-free while maintaining indicated treatments. Conditioned medium was collected on day 5 and centrifuged at 4,000 rpm for 5 min at 4 °C to remove any debris. 100 µl conditioned medium was flash frozen and sent to Eve Technologies (Calgary, Canada) for analysis using the Discovery Assay® 65-Plex Human Cytokine Array/Chemokine Array Panel (HD65). Results are presented as the fold change in concentration (pg/mL) following E2 treatment relative to vehicle.

### Migration assays

Cells were seeded in 10 cm dishes and treated for five days with indicated treatments with a media change and treatment refresh on day 3. At the time of the media change, cells were lifted and re-seeded at 30,000 cells/well in IncuCyte® ImageLock 96-well plates while continuing pretreatment. After 5 days of treatment, wounds were created using the IncuCyte® WoundMaker Tool (#4563, Sartorius), cells were washed once, and medium with treatment was re-added. Plates were then placed in the IncuCyte® S3 machine and imaged once every two hours using the Scratch Wound Protocol. Wound closure was assessed using the Cell Migration Analysis software module (#9600-0012, Sartorius).

### siRNA-mediated knockdown of NFκB

Pooled siRNAs designed to specifically target human p65 were purchased from Dharmacon (Lafayette, CO, USA). Cells were transfected with 5 nM ON-TARGETplus SMARTpool siRNA using DharmaFECT 1 transfection reagent (T-2005-01, Dharmacon) according to the manufacturer’s protocol. Non-Targeting siRNA Pool 1 (D-001206-13, Dharmacon) was used as a negative control. Cells were transfected with siRNAs 24 h prior to performing indicated treatments.

### Luciferase assays

Cells were seeded in 24-well plates and treated with dox for 24 h prior to transfecting with 100 ng of a pGL3 luciferase reporter construct containing NFκB response elements (NRE) using FuGENE 6 (Roche, Basel, Switzerland). Twenty-four hours post-transfection, cells were treated with vehicle control, 1 nM E2, 20 ng/mL TNFα, or E2 + TNFα for an additional 24 h. Cells were washed once with 1X PBS and lysed using 1X Passive Lysis Buffer (Promega). Equal amounts of protein lysate were assessed for luciferase activity using Luciferase Assay Reagent and a Glomax-Dual Luminometer (Promega). Treatments were conducted in replicates of 6.

### p65 nuclear localization assay

MDA-MB-231-ERβ cells were seeded in four-well Nunc™ Lab-Tek™ II Chamber Slides™ (Thermo Fisher) and treated in duplicate the following day with dox and vehicle or 1 nM E2. After 24 h, 20 ng/ml TNFα was added for 30 min. Cells were washed with 1X PBS, fixed for 15 min at room temperature with 4% formalin freshly diluted in 1X PBS from 16% formalin (Electron Microscopy Sciences), and permeabilized for 15 min at room temperature with 1X PBST. Cells were blocked for 30 min at room temperature using SuperBlock™ (PBS) Blocking Buffer (Thermo Fisher) and incubated with primary antibody overnight at 4 °C. Slides were washed with 1X PBST, separated from the chambers, and incubated with fluorescent-labeled secondary antibody and Hoechst 33258 (Thermo Fisher) for 30 min at room temperature in the dark. Cells were washed with PBST and rinsed with PBS prior to mounting with coverslips using ProLong™ Gold Antifade Mountant (Thermo Fisher). Slides were imaged using an LSM 780 inverted confocal microscope (Zeiss, Oberkochen, Germany) and ZEN Black software (Zeiss). Antibody information can be found in Supplemental Table 5.

### Cell fractionation and co-immunoprecipitation

For cell fractionation, cells were plated in replicates of four in 15 cm dishes and allowed to adhere for 24 h. Cells were treated as indicated for 2 h, medium was removed, cells were washed with ice cold PBS, and pellets were collected in 1X PBS for subsequent nuclear, cytoplasmic, or whole cell lysate preparation. Whole cell lysates were prepared in the same manner as for western blotting. Cytoplasmic protein was extracted by incubating cells with cytoplasmic lysis buffer (10 mM Tris-HCl pH 8.0, 10 mM KCl, 0.1 mM EGTA, 0.1 mM EDTA) containing PI for 15 min on ice. Ten percent NP-40 was added, followed by vortexing and centrifugation at 14,000 rpm for 15 min at 4 °C. Supernatants were saved as cytoplasmic extracts. Remaining pellets were washed twice with cytoplasmic lysis buffer, then resuspended in nuclear lysis buffer (20 mM Tris-HCl pH 8.0, 0.4 M NaCl, 0.1 mM EGTA, 0.1 mM EDTA) containing PI for 30 min on ice. Following incubation, lysates were centrifuged at 14,000 rpm for 15 min at 4 °C and the supernatant was saved as nuclear extract.

Protein concentrations were determined using the Pierce™ BCA Protein Assay Kit. Five hundred micrograms of protein were used for overnight immunoprecipitation at 4 °C. Following immunoprecipitation, protein complexes were captured using 40 µl Protein G Dynabeads™ (Thermo Fisher) for 2 h at 4 °C with rotation. Beads were washed three times with NETN buffer and protein was eluted via boiling with 2X Laemmli Sample Buffer (Bio-Rad) containing β-mercaptoethanol for 5 min. Immunoprecipitated samples were subjected to western blotting, along with 40 µg of nuclear extract that was not subjected to immunoprecipitation as an input loading control. Antibody information can be found in Supplemental Table 5.

### Chromatin immunoprecipitation followed by PCR and sequencing

Cells were plated in 10 cm dishes and treated in triplicate as indicated above for 5 days, followed by fixation for 10 min at room temperature with 1% paraformaldehyde and quenched with 125 mM Glycine (Sigma-Aldrich) for 5 min at room temperature. Nuclear extracts were prepared and immunoprecipitation with specific antibodies was carried out as previously described^78,79^. ChIP-PCR was performed using the same conditions described for RT-qPCR using the specified ChIP-specific primers (Supplemental Tables 6) and 2µl chromatin solution (diluted 1:400).

For H3K27me3 and H3K27ac ChIP-seq, samples were prepared in an identical fashion. For p65 and RNA Pol II phospho-Ser2 ChIP-seq, fixed cell pellets were processed as previously described^80^ by the Mayo Clinic Epigenomics Development Laboratory (Rochester, MN, USA). ChIP-seq libraries were prepared from immunoprecipitated chromatin solutions and input DNA using the ThruPLEX® DNA-seq Kit V2 (Rubicon Genomics, Ann Arbor, MI, USA). Libraries were sequenced using 50 base pair paired-end sequencing on an Illumina HiSeq 4000. Raw sequencing reads were analyzed using the HiChIP pipeline^81^ to generate library-size normalized signal tracks for visualization and a list of peaks. Briefly, paired-end reads were mapped to the human reference genome (hg38) by Burrows-Wheeler Alignment (BWA)^82^ with default settings, and only pairs with at least one of the ends being uniquely mapped were retained for further analysis. Alignments were position sorted and duplicates were removed using the Picard tools (https://broadinstitute.github.io/picard/). Peaks were called using the MACS2 algorithm at FDR ≤ 1%. Visualization tracks and heat maps were generated by deeptools 2.0.

### Statistical analyses

A Fisher’s exact test was used to assess differences between those with ERβ+ TNBC and those with ERβ− TNBC with respect to patient and disease characteristic at primary diagnosis (Table 1). *P* values ≤ 0.05 were considered to be statistically significant. Cox modeling was performed to assess whether overall survival differed with respect to ERβ expression after adjusting for known prognostic factors and administration of adjuvant chemotherapy. Analyses were carried out using SAS 9.3. All in vitro experiments were conducted in biological replicates of at least three and with 3–6 technical replicates per assay. Representative data sets are shown. Student’s *t-*tests, one-way ANOVAs, and Wilcoxon Rank tests were used to determine statistically significant differences between treatments as indicated. *P* values ≤ 0.05 were considered statistically significant. Graphs and analyses were generated using GraphPad Prism 8 (GraphPad Software, San Diego, CA, USA).

### Reporting summary

Further information on research design is available in the Nature Research Reporting Summary linked to this article.

## Supplementary information

Reporting Summary Checklist

Supplemental Material

## Data Availability

All RNA and ChIP sequencing data that support the findings of this study have been deposited in the National Center for Biotechnology Information Gene Expression Omnibus (GEO) and are accessible through the GEO Series accession numbers GSE108981, GSE155684 and GSE155685. All other relevant data are included in the manuscript or available from the corresponding author upon request.

## Code availability

No custom codes were used to analyze the data presented in this manuscript and all codes are publically available.
