# Supplementary material for: Estrogen receptor beta repurposes EZH2 to suppress oncogenic NFκB/p65 signaling in triple negative breast cancer
Source: NPJ Breast Cancer. 2022 Feb 17;8:20. doi: 10.1038/s41523-022-00387-0 (PMC8854734; doi:10.1038/s41523-022-00387-0)
Supplement: Supplementary file 1 — Reporting Summary Checklist [file 41523_2022_387_MOESM1_ESM.pdf]

nature portfolio

Corresponding author(s): John R. Hawse

Last updated by author(s): Dec 22, 2021

Reporting Summary

Nature Portfolio wishes to improve the reproducibility of the work that we publish. This form provides structure for consistency and transparency in reporting. For further information on Nature Portfolio policies, see our Editorial Policies and the Editorial Policy Checklist.

Statistics

For all statistical analyses, confirm that the following items are present in the figure legend, table legend, main text, or Methods section.

n/a	Confirmed	
☐	☒	The exact sample size (*n*) for each experimental group/condition, given as a discrete number and unit of measurement
☐	☒	A statement on whether measurements were taken from distinct samples or whether the same sample was measured repeatedly
☐	☒	The statistical test(s) used AND whether they are one- or two-sided<br>*Only common tests should be described solely by name; describe more complex techniques in the Methods section.*
☐	☒	A description of all covariates tested
☐	☒	A description of any assumptions or corrections, such as tests of normality and adjustment for multiple comparisons
☐	☒	A full description of the statistical parameters including central tendency (e.g. means) or other basic estimates (e.g. regression coefficient) AND variation (e.g. standard deviation) or associated estimates of uncertainty (e.g. confidence intervals)
☐	☒	For null hypothesis testing, the test statistic (e.g. *F*, *t*, *r*) with confidence intervals, effect sizes, degrees of freedom and *P* value noted<br>*Give P values as exact values whenever suitable.*
☒	☐	For Bayesian analysis, information on the choice of priors and Markov chain Monte Carlo settings
☐	☒	For hierarchical and complex designs, identification of the appropriate level for tests and full reporting of outcomes
☐	☒	Estimates of effect sizes (e.g. Cohen's *d*, Pearson's *r*), indicating how they were calculated

Our web collection on statistics for biologists contains articles on many of the points above.

Software and code

Policy information about availability of computer code

Data collection	No software was used for data collection.
Data analysis	For RNAseq, mapped reads were assigned using featureCounts and batch effect correction was performed with RUVseq using a curated set of housekeeping genes to normalize the batches. An RPKM cutoff was used to remove lowly expressed genes (RPKM<1) from further analyses. Comparison tests were performed using edgeR and significance was measured using |log2(fold change)|≥1.5, p<0.05, and FDR≤0.1. Canonical pathway and upstream regulator identification with Ingenuity Pathway Analysis software (IPA, Ingenuity Systems, Inc., Redwood City, CA, USA; http://www.ingenuity.com). Associations of gene signatures derived from RNAseq data using MDA-MB-231-ERβ cell lines and human tumors with publically available databases were performed using Gene Set Enrichment Analysis (GSEA). A Fisher's exact test was used to assess differences between those with ERβ+ TNBC and those with ERβ- TNBC with respect to patient and disease characteristic at primary diagnosis (Table 1). P-values ≤ 0.05 were considered to be statistically significant. Cox modeling was performed to assess whether overall survival differed with respect to ERβ expression after adjusting for known prognostic factors and administration of adjuvant chemotherapy. Analyses were carried out using SAS 9.3. All in vitro experiments were conducted in biological replicates of at least 3 and with 3-6 technical replicates per assay. Representative data sets are shown. Student's t-tests, one-way ANOVAs, and Wilcoxon Rank tests were used to determine statistically significant differences between treatments as indicated. P-values ≤ 0.05 were considered statistically significant. Graphs and analyses were generated using GraphPad Prism 8 (GraphPad Software, San Diego, CA, USA).

For manuscripts utilizing custom algorithms or software that are central to the research but not yet described in published literature, software must be made available to editors and reviewers. We strongly encourage code deposition in a community repository (e.g. GitHub). See the Nature Portfolio guidelines for submitting code & software for further information.

Data

Policy information about availability of data

All manuscripts must include a data availability statement. This statement should provide the following information, where applicable:
- Accession codes, unique identifiers, or web links for publicly available datasets
- A description of any restrictions on data availability
- For clinical datasets or third party data, please ensure that the statement adheres to our policy

All analyzed datasets generated during the current study are included in this published article (and its supplementary information files). The raw data for the RNAseq and ChIPseq data are available in the Gene Expression Omnibus (GEO) under accession numbers GSE 108981, GSE 155684 and GSE 155685.

Field-specific reporting

Please select the one below that is the best fit for your research. If you are not sure, read the appropriate sections before making your selection.

☒ Life sciences ☐ Behavioural & social sciences ☐ Ecological, evolutionary & environmental sciences

For a reference copy of the document with all sections, see nature.com/documents/nr-reporting-summary-flat.pdf

Life sciences study design

All studies must disclose on these points even when the disclosure is negative.

Sample size	No sample size calculations were required for the studies detailed in this publication. All in vitro studies utilized replicates of 3-6 and all experiments were repeated a minimum of 3 times. These samples sizes were chosen based on previous studies utilizing similar techniques and/or as recommended by the scientific community.
Data exclusions	No data were excluded from this study.
Replication	All experiments were conducted in replicates of 3-6 and repeated a minimum of 3 times. All repeats produced the same patterns and outcomes with non statistically significant differences between independent repeats.
Randomization	Randomization was not required by any of the experiments, assays or data analyses performed in this manuscript.
Blinding	Blinding was not necessary nor was it possible. For the clinical cohort, it was required that ER-beta positive and ER-beta negative patients be identified and group together for analysis. For all of the in vitro studies, individuals performing the experiments were also responsible for data analysis. Critically important experiments were conducted independently by a second member of the research team to ensure reproducibility.

Reporting for specific materials, systems and methods

We require information from authors about some types of materials, experimental systems and methods used in many studies. Here, indicate whether each material, system or method listed is relevant to your study. If you are not sure if a list item applies to your research, read the appropriate section before selecting a response.

Materials & experimental systems

n/a	Involved in the study	
☐	☒	Antibodies
☐	☒	Eukaryotic cell lines
☒	☐	Palaeontology and archaeology
☒	☐	Animals and other organisms
☒	☐	Human research participants
☐	☒	Clinical data
☒	☐	Dual use research of concern

Methods

n/a	Involved in the study	
☐	☒	ChIP-seq
☒	☐	Flow cytometry
☒	☐	MRI-based neuroimaging

Antibodies

Antibodies used	All of this information is provided in Supplemental Table 5.
Validation	Validation of antibody performance was determined using knockdown or over expression of the target protein. Further, all antibodies utilized in this publication have been validated for the specified applications by the vendor.

Eukaryotic cell lines

Policy information about cell lines

Cell line source(s)	HCC1937, HCC1143, MDA-MB-453, MDA-MB-436, BT549, MDA-MB-231, BT20, MDA-MB-468, and Hs578T cells were obtained from ATCC. SUM185 and SUM159 cells were purchased from BioIVT (Westbury, NY, USA).
Authentication	Cell lines were authenticated by IDEXX.
Mycoplasma contamination	All cell lines were routinely tested and confirmed to be mycoplasm negative.
Commonly misidentified lines (See ICLAC register)	None.

Clinical data

Policy information about clinical studies

All manuscripts should comply with the ICMJE guidelines for publication of clinical research and a completed CONSORT checklist must be included with all submissions.

Clinical trial registration	NA
Study protocol	NA
Data collection	Patients included in the Mayo Clinic TNBC cohort represent women diagnosed with triple negative breast cancer in Rochester MN between 1985 and 2012.
Outcomes	Overall Survival was the only outcome measure included in this publication. Cox modeling was performed to assess whether overall survival differed with respect to ERβ expression after adjusting for known prognostic factors and administration of adjuvant chemotherapy.

ChIP-seq

Data deposition

☒ Confirm that both raw and final processed data have been deposited in a public database such as GEO.

☒ Confirm that you have deposited or provided access to graph files (e.g. BED files) for the called peaks.

Data access links
May remain private before publication.

https://www.ncbi.nlm.nih.gov/geo/query/acc.cgi

Files in database submission

Less... Less...
GSM2918262 jc2831_MDA-MB-231-ERb_ERb_Full_24_hrs_Dox_3_hrs_ethanol
GSM2918263 jc2834_MDA-MB-231-ERb_ERb_Full_24_hrs_Dox_3_hrs_ethanol
GSM2918264 jc2836_MDA-MB-231-ERb_ERb_Full_24_hrs_Dox_3_hrs_ethanol
GSM2918265 jc2847_MDA-MB-231-ERb_ERb_Full_24_hrs_Dox_3_hrs_ethanol
GSM2918266 jc2829_MDA-MB-231-ERb_ERb_Full_24_hrs_Dox_3_hrs_1nM_Estradiol
GSM2918267 jc2842_MDA-MB-231-ERb_ERb_Full_24_hrs_Dox_3_hrs_1nM_Estradiol
GSM2918268 jc2844_MDA-MB-231-ERb_ERb_Full_24_hrs_Dox_3_hrs_1nM_Estradiol
GSM2918269 jc2848_MDA-MB-231-ERb_ERb_Full_24_hrs_Dox_3_hrs_1nM_Estradiol
GSM2918270 jc2833_MDA-MB-231-ERb_ERb_Full_24_hrs_Dox_3_hrs_1nM_LY500307
GSM2918271 jc2837_MDA-MB-231-ERb_ERb_Full_24_hrs_Dox_3_hrs_1nM_LY500307
GSM2918272 jc2841_MDA-MB-231-ERb_ERb_Full_24_hrs_Dox_3_hrs_1nM_LY500307
GSM2918273 jc2846_MDA-MB-231-ERb_ERb_Full_24_hrs_Dox_3_hrs_1nM_LY500307
GSM2918274 jc2832_MDA-MB-231-ERb_Input_Full_24_hrs_Dox_3_hrs_ethanol
GSM2918275 jc2839_MDA-MB-231-ERb_Input_Full_24_hrs_Dox_3_hrs_ethanol
GSM2918276 jc2843_MDA-MB-231-ERb_Input_Full_24_hrs_Dox_3_hrs_ethanol
GSM2918277 jc2830_MDA-MB-231-ERb_Input_Full_24_hrs_Dox_3_hrs_1nM_Estradiol
GSM2918278 jc2838_MDA-MB-231-ERb_Input_Full_24_hrs_Dox_3_hrs_1nM_Estradiol
GSM2918279 jc2845_MDA-MB-231-ERb_Input_Full_24_hrs_Dox_3_hrs_1nM_Estradiol
GSM2918280 jc2835_MDA-MB-231-ERb_Input_Full_24_hrs_Dox_3_hrs_1nM_LY500307
GSM2918281 jc2840_MDA-MB-231-ERb_Input_Full_24_hrs_Dox_3_hrs_1nM_LY500307
GSM2918282 jc2849_MDA-MB-231-ERb_Input_Full_24_hrs_Dox_3_hrs_1nM_LY500307

Less... Less...
GSM4710482 MDA231ERb_me3ac_Input_veh_rep1
GSM4710483 MDA231ERb_me3ac_Input_veh_rep2
GSM4710484 MDA231ERb_me3ac_Input_E2_rep1
GSM4710485 MDA231ERb_me3ac_Input_E2_rep2
GSM4710486 MDA231ERb_me3ac_Input_TNFa_rep1
GSM4710487 MDA231ERb_me3ac_Input_TNFa_rep2

GSM4710488 MDA231ERb_me3ac_Input_E2+TNFa_rep1
GSM4710489 MDA231ERb_me3ac_Input_E2+TNFa_rep2
GSM4710490 MDA231ERb_veh_H3K27me3_rep1
GSM4710491 MDA231ERb_veh_H3K27me3_rep2
GSM4710492 MDA231ERb_E2_H3K27me3_rep1
GSM4710493 MDA231ERb_E2_H3K27me3_rep2
GSM4710494 MDA231ERb_TNFa_H3K27me3_rep1
GSM4710495 MDA231ERb_TNFa_H3K27me3_rep2
GSM4710496 MDA231ERb_E2+TNFa_H3K27me3_rep1
GSM4710497 MDA231ERb_E2+TNFa_H3K27me3_rep2
GSM4710498 MDA231ERb_veh_H3K27ac_rep1
GSM4710499 MDA231ERb_veh_H3K27ac_rep2
GSM4710500 MDA231ERb_E2_H3K27ac_rep1
GSM4710501 MDA231ERb_E2_H3K27ac_rep2
GSM4710502 MDA231ERb_TNFa_H3K27ac_rep1
GSM4710503 MDA231ERb_TNFa_H3K27ac_rep2
GSM4710504 MDA231ERb_E2+TNFa_H3K27ac_rep1
GSM4710505 MDA231ERb_E2+TNFa_H3K27ac_rep2
GSM4710506 MDA231ERb_NFkBRNAP_Input_veh_rep1
GSM4710507 MDA231ERb_NFkBRNAP_Input_veh_rep2
GSM4710508 MDA231ERb_NFkBRNAP_Input_E2_rep1
GSM4710509 MDA231ERb_NFkBRNAP_Input_E2_rep2
GSM4710510 MDA231ERb_NFkBRNAP_Input_TNFa_rep1
GSM4710511 MDA231ERb_NFkBRNAP_Input_TNFa_rep2
GSM4710512 MDA231ERb_NFkBRNAP_Input_E2+TNFa_rep1
GSM4710513 MDA231ERb_NFkBRNAP_Input_E2+TNFa_rep2
GSM4710514 MDA231ERb_veh_NFkB_rep1
GSM4710515 MDA231ERb_veh_NFkB_rep2
GSM4710516 MDA231ERb_E2_NFkB_rep1
GSM4710517 MDA231ERb_E2_NFkB_rep2
GSM4710518 MDA231ERb_TNFa_NFkB_rep1
GSM4710519 MDA231ERb_TNFa_NFkB_rep2
GSM4710520 MDA231ERb_E2+TNFa_NFkB_rep1
GSM4710521 MDA231ERb_E2+TNFa_NFkB_rep2
GSM4710522 MDA231ERb_veh_RNAPIIpS2
GSM4710523 MDA231ERb_E2_RNAPIIpS2
GSM4710524 MDA231ERb_TNFa_RNAPIIpS2
GSM4710525 MDA231ERb_E2+TNFa_RNAPIIpS2
GSM4710526 MDA231ERb_veh_rep1
GSM4710527 MDA231ERb_veh_rep2
GSM4710528 MDA231ERb_veh_rep3
GSM4710529 MDA231ERb_TNFa_rep1
GSM4710530 MDA231ERb_TNFa_rep2
GSM4710531 MDA231ERb_TNFa_rep3
GSM4710532 MDA231ERb_E2_rep1
GSM4710533 MDA231ERb_E2_rep2
GSM4710534 MDA231ERb_E2_rep3
GSM4710535 MDA231ERb_E2+TNFa_rep1
GSM4710536 MDA231ERb_E2+TNFa_rep2
GSM4710537 MDA231ERb_E2+TNFa_rep3

Genome browser session (e.g. UCSC)

No longer applicable.

Methodology

Replicates

All experiments were conducted in replicates of 3-6 and repeated a minimum of 3 times. All repeats produced the same patterns and outcomes with non statistically significant differences between independent repeats.

Sequencing depth

Paired end sequencing was utilized with an average of 50 million reads per sample.

Antibodies

All of this information is included in Supplemental Table 5.

Peak calling parameters

paired-end reads were mapped to the human reference genome (hg38) by Burrows-Wheeler Alignment (BWA) with default settings, and only pairs with at least one of the ends being uniquely mapped were retained for further analysis. Alignments were position sorted and duplicates were removed using the Picard tools (https://broadinstitute.github.io/picard/). Peaks were called using the MACS2 algorithm at FDR≤1%. Visualization tracks and heat maps were generated by deeptools 2.0.

Data quality

For each sample, FastQC was used to report the distribution of average per-base and per-read quality, as well as the level of duplication and possible sources of contaminations. In the samples included as part of this publication, there was no indication of abnormalities in mapping results. For all of the ChIPseq datasets included here, at least 50% of peaks had greater than 5 fold enrichment above input levels and relative to vehicle treatments with FDR less than 5%.

Software

ChIP-seq libraries were prepared from immunoprecipitated chromatin solutions and input DNA using the ThruPLEX® DNA-seq Kit V2 (Rubicon Genomics, Ann Arbor, MI, USA). Libraries were sequenced using 50 base pair paired-end sequencing on an Illumina HiSeq

4000. Raw sequencing reads were analyzed using the HiChIP pipeline to generate library-size normalized signal tracks for visualization and a list of peaks. Briefly, paired-end reads were mapped to the human reference genome (hg38) by Burrows-Wheeler Alignment (BWA) with default settings, and only pairs with at least one of the ends being uniquely mapped were retained for further analysis. Alignments were position sorted and duplicates were removed using the Picard tools (https://broadinstitute.github.io/picard/). Peaks were called using the MACS2 algorithm at FDR≤1%. Visualization tracks and heat maps were generated by deeptools 2.0.
