# Supplementary material for: Estrogen receptor beta repurposes EZH2 to suppress oncogenic NFκB/p65 signaling in triple negative breast cancer
Source: NPJ Breast Cancer. 2022 Feb 17;8:20. doi: 10.1038/s41523-022-00387-0 (PMC8854734; doi:10.1038/s41523-022-00387-0)
Supplement: Supplementary file 2 — Supplemental Material [file 41523_2022_387_MOESM2_ESM.pdf]

SUPPLEMENTARY MATERIAL

SUPPLEMENTAL FIGURES

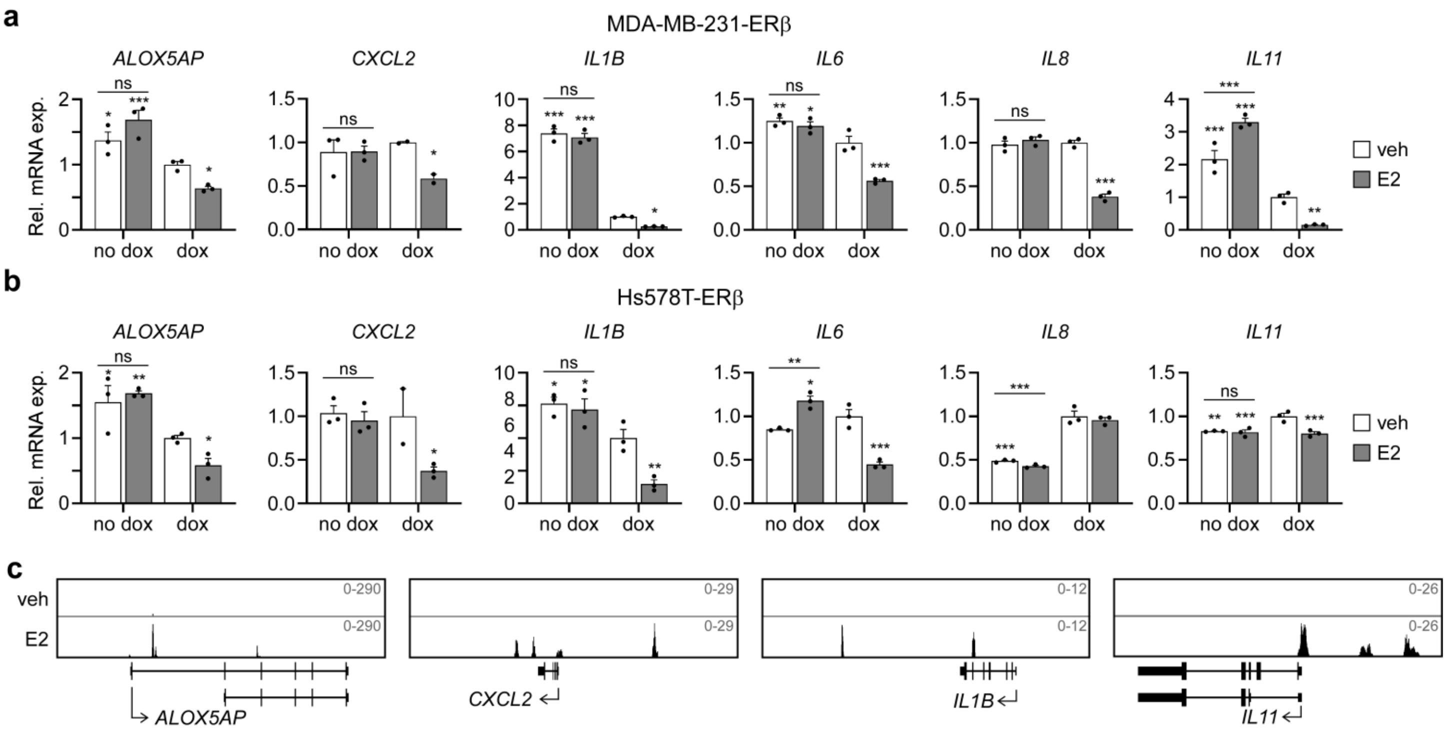

Supplementary Figure 1. RT-qPCR analysis of NFκB/p65 target gene expression in **(a)** MDA-MB-231-ERβ cells and **(b)** Hs578T-ERβ cells following vehicle or E2 treatment in the presence (+dox) and absence (-dox) of ERβ expression. Data is presented as mean ± SEM. *P<0.05, **P<0.01, ***P<0.001 relative to veh+dox and between indicated treatments (one-way ANOVA). **(c)** ERβ ChIP-seq tracks at NFκB/p65 target gene loci following veh and E2 treatment.

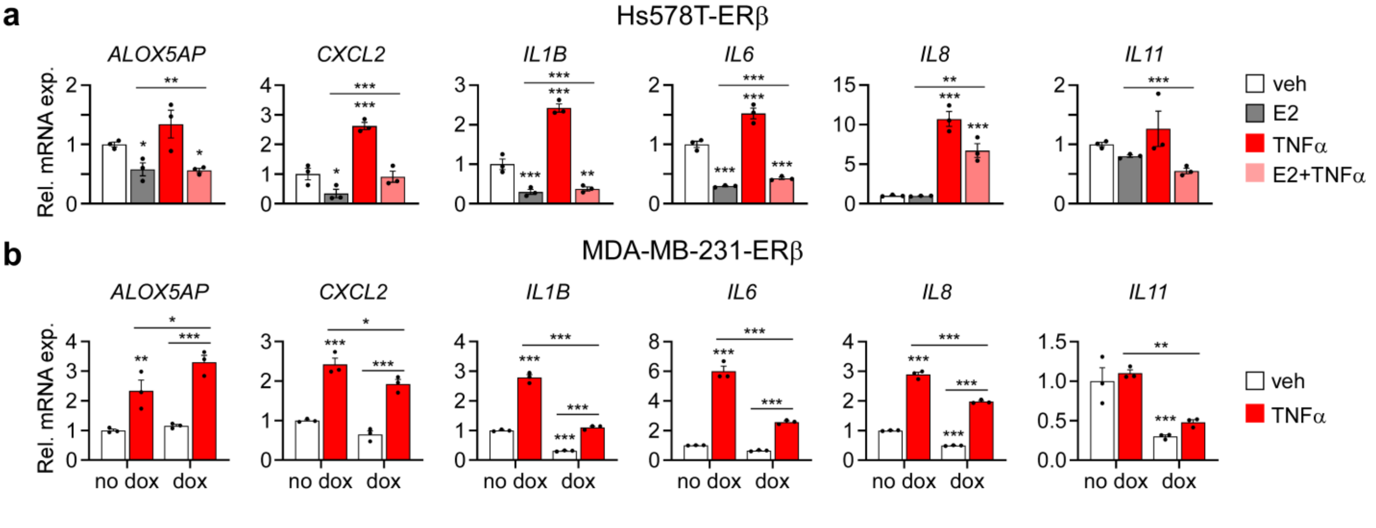

Supplementary Figure 2. RT-qPCR analysis of NFκB/p65 target gene expression in **(a)** Hs578T-ERβ cells following veh, E2, TNFα, and E2+TNFα treatment in the presence of ERβ and **(b)** in MDA-MB-231-ERβ cells following veh or TNFα treatment in the presence (dox) and absence (no dox) of ERβ expression relative to veh-dox. *P<0.05, **P<0.01, ***P<0.001 relative to veh + dox (panel A) or veh - dox (panel B) and between indicated treatments (one-way ANOVA). All data is presented as mean ± SEM.

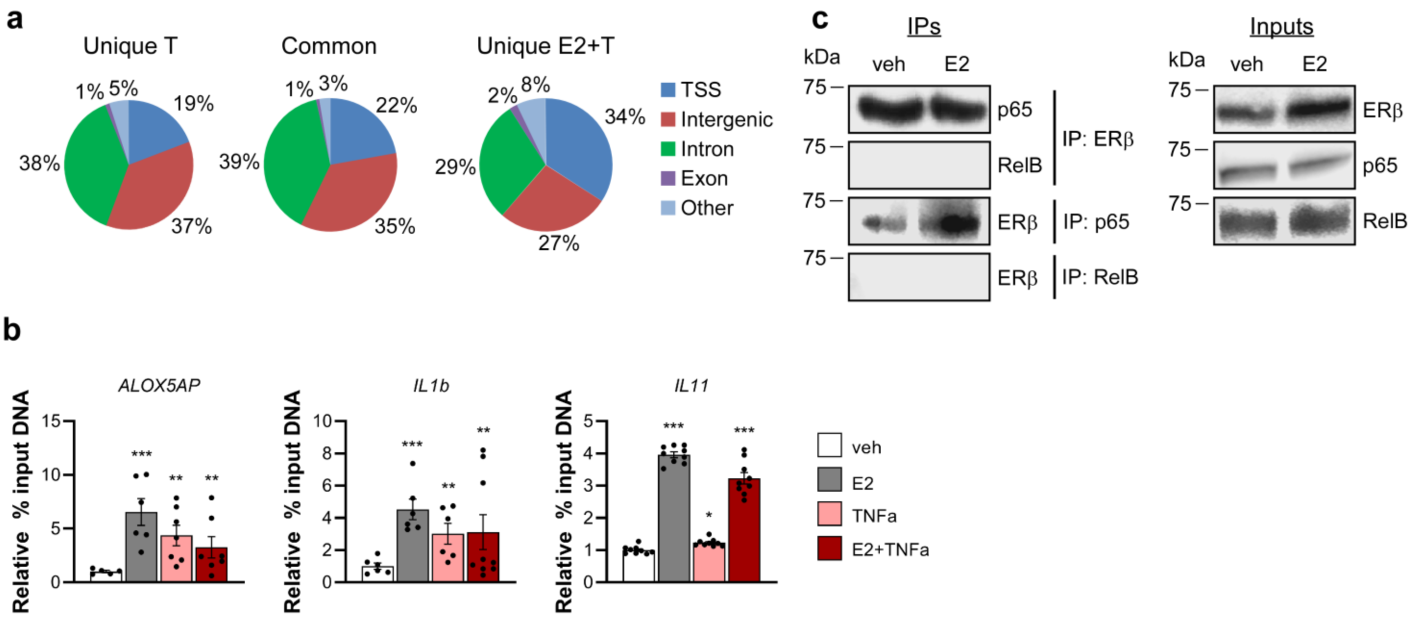

Supplementary Figure 3. (a) Genomic distribution of p65 binding in MDA-MB-231-ERβ cells at sites detected via ChIP-seq following treatment with only TNFα (Unique T), only E2+TNFα (Unique E+T), or both (Common). **(b)** ChIP-PCR for ERβ at 3 identified p65 binding sites that encode an NRE in the absence of a nearby ERE. *P<0.05, **P<0.01, ***P<0.001 relative to veh + dox treatment (one-way ANOVA). All data is presented as mean ± SEM. **(c)** Co-IP of HEK293T nuclear lysates that were immunoprecipitated with ERβ, p65, or RELB specific antibodies followed by western blotting with indicated antibodies. Non-immunoprecipitated inputs from nuclear lysates are shown as loading controls.

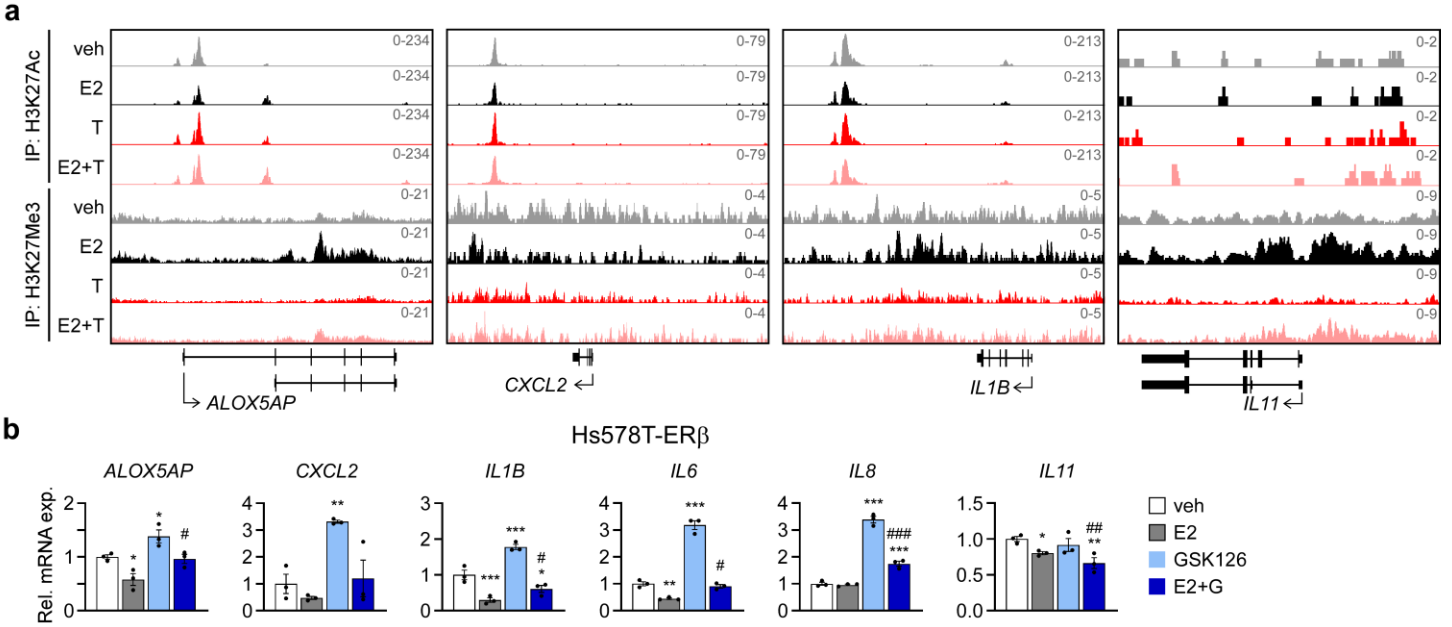

Supplementary Figure 4. (a) Example ChIP-seq tracks at NFκB/p65 target gene loci for H3K27ac and H3K27me3 following veh, E2, TNFα (T), and E2+TNFα (E2+T) treatment. **(b)** mRNA expression of NFκB/p65 target genes in Hs578T cells following veh E2, 5µM GSK126, or E2+GSK126 as determined by RT-qPCR. Data is presented as mean ± SEM. *P<0.05, **P<0.01, ***P<0.001 relative to veh + dox and #P<0.05, #P<0.01, #P<0.001 relative to E2 alone (one-way ANOVA).

SUPPLEMENTAL TABLES

Supplemental Table 1. Genes significantly regulated following 5 days of 1nM E2 treatment of ERβ expressing MDA-MB-231 cells.

Gene	logFC	logCPM	PValue	FDR
F13A1	10.13065864	4.616093019	6.52E-143	1.02E-139
CXCL14	9.755813283	7.475945458	4.22E-121	3.11E-118
CST5	9.353252791	3.844952224	6.94E-63	1.36E-60
KRT13	9.231876262	7.347656485	4.13E-91	1.91E-88
CST2	8.997605987	5.746488363	5.32E-83	1.85E-80
CST1	8.893111846	10.13262154	1.32E-55	2.29E-53
TNS4	8.638163291	7.415758926	4.74E-228	5.93E-224
MAB21L4	8.560469278	3.939475086	2.10E-101	1.31E-98
MGAT3	8.13293599	3.517365704	1.07E-80	3.25E-78
LOXL4	8.087182946	11.58090912	1.25E-150	2.61E-147
CST4	8.057760205	9.021202136	1.32E-54	2.26E-52
SEMA3B	7.811443988	9.150709877	1.87E-34	1.56E-32
CCN5	7.702070558	3.388691465	1.07E-57	1.91E-55
ALPP	7.683086111	8.767889873	1.21E-121	9.48E-119
S100A7	7.481410477	2.024144911	3.05E-22	1.35E-20
TMOD1	7.446053467	4.816867365	1.68E-99	9.54E-97
SERPINA9	7.416888746	4.046871036	8.97E-63	1.73E-60
ALPG	7.406099416	5.075516334	5.33E-74	1.33E-71
TMPRSS3	7.21350032	2.909086415	1.40E-52	2.13E-50
CYTH4	7.144312093	4.676202354	2.89E-145	5.18E-142
GJA5	7.136094236	2.838371865	9.79E-56	1.73E-53
CADM1	7.025599654	-0.919392803	6.08E-05	0.000323686
TFF1	7.013501867	-0.889260788	5.08E-10	7.15E-09
FGFBP1	6.991919838	1.551057804	1.07E-22	4.94E-21
TUBA3E	6.882127604	2.296631269	1.11E-40	1.22E-38
HAVCR2	6.749698555	5.600428538	7.59E-127	7.31E-124
GCNT3	6.715653947	2.669088218	3.60E-44	4.51E-42
DPYSL3	6.623455313	-1.217271052	2.72E-06	1.98E-05
B3GNT6	6.542503188	1.606570862	3.24E-28	2.06E-26
SYT8	6.249886285	-1.455958991	1.93E-06	1.45E-05
CPZ	6.247967884	1.692285641	1.97E-31	1.44E-29
ACKR3	6.234599023	2.749008237	1.43E-46	1.95E-44
OLFML3	6.19657661	3.744672005	4.80E-78	1.37E-75
S100A9	6.005302547	1.755060286	7.68E-22	3.25E-20
SERPINA6	5.936584377	1.037440018	2.87E-21	1.17E-19
ABCB1	5.871302181	-0.218204037	7.13E-13	1.34E-11
SLC2A5	5.865062118	2.050717302	7.91E-21	3.18E-19
INHBB	5.847414156	5.230902526	3.34E-127	3.48E-124
LUM	5.755536304	-1.830008735	0.000397548	0.001719136
VASN	5.685216909	7.354924912	2.35E-65	4.98E-63
GPR78	5.658735367	-1.87948241	0.000835599	0.003277213
CALHM5	5.550579772	1.044382406	4.54E-17	1.31E-15
PLEKHS1	5.55027047	2.961732353	2.89E-43	3.47E-41
CLEC4M	5.431112923	3.592941829	1.57E-34	1.32E-32
IL1R2	5.408502248	1.958265852	2.98E-30	2.08E-28
GASK1B	5.295729879	1.996007481	4.59E-28	2.89E-26

CDH1	5.294007211	5.237886119	6.94E-54	1.16E-51
PADI4	5.268762143	3.85701502	1.55E-76	4.23E-74
RNF223	5.218073457	4.188101708	1.12E-86	4.67E-84
NAT8	5.09839652	-0.179795023	2.91E-13	5.73E-12
C1orf116	5.081920172	5.252161946	8.56E-85	3.25E-82
GJA1	5.06937784	5.020329807	5.61E-39	5.80E-37
ELF3	5.052133997	6.740359002	1.48E-220	9.27E-217
NXPH3	4.981153122	3.504555658	8.80E-48	1.22E-45
PDZK1	4.980722583	4.326192213	2.82E-79	8.39E-77
TFF2	4.936357578	1.780275219	7.56E-28	4.69E-26
LAMC2	4.903889277	9.128859501	1.94E-108	1.28E-105
GREB1	4.88004641	5.283384284	2.07E-83	7.41E-81
SPATA46	4.877243886	0.695477138	4.21E-16	1.09E-14
KLRC2	4.811904607	3.31041594	2.06E-22	9.28E-21
FRMPD3	4.745178367	3.614999577	6.87E-34	5.55E-32
IGFBP5	4.730937085	-0.067394046	5.17E-05	0.000280847
PRB4	4.651948297	-2.436942615	0.025769804	0.060732713
RAB37	4.639441158	4.89797184	8.35E-58	1.52E-55
FRAS1	4.611816596	5.582180019	2.78E-40	2.97E-38
MPPED1	4.602265009	-2.440020085	0.009986809	0.027475447
FRK	4.589087682	0.152904112	2.16E-08	2.33E-07
KLRC3	4.586176203	2.406895074	4.47E-25	2.34E-23
LYZ	4.569375139	0.164606113	5.22E-12	9.11E-11
SYBU	4.53970004	3.514377198	4.89E-50	7.12E-48
PLET1	4.487845741	0.345418156	6.16E-12	1.07E-10
FAXDC2	4.449053202	5.194461589	6.22E-90	2.78E-87
SFTPD	4.339686464	1.454172138	9.51E-20	3.48E-18
RASGRP1	4.323150442	3.441162486	2.57E-52	3.88E-50
SPATA31D4	4.303464578	2.135016137	2.53E-19	8.93E-18
PRSS23	4.259063999	10.71230772	4.25E-155	1.06E-151
NAP1L3	4.259058229	0.576981046	1.75E-09	2.27E-08
RETREG1	4.217347085	1.716805039	1.63E-12	2.96E-11
KCNK15	4.201091447	2.382001015	2.85E-34	2.35E-32
LSMEM2	4.173212325	2.009125669	2.20E-12	3.96E-11
ISM1	4.144986921	1.471032861	1.17E-16	3.21E-15
NCAM2	4.143263652	2.37253867	4.59E-16	1.18E-14
B4GALNT2	4.101379142	0.239530834	2.10E-09	2.69E-08
ADAM12	4.06213761	3.576064122	9.41E-37	9.13E-35
KLRC4	4.042692871	-2.661951423	0.045303513	0.096586286
SYT1	4.031928075	1.061585011	1.24E-10	1.90E-09
DNAJC12	4.030336106	-1.631251251	5.63E-05	0.000302645
AFF3	4.008625313	2.59086	1.84E-36	1.74E-34
ABCG2	3.987538016	2.177764792	1.80E-17	5.38E-16
DAPK2	3.93662756	4.60778551	7.69E-81	2.41E-78
LGALS9	3.929775414	2.787065904	2.49E-16	6.61E-15
CALB2	3.911964561	7.681131278	2.46E-156	7.70E-153
SPATA31D3	3.908846686	1.841681384	9.57E-24	4.70E-22
IRX3	3.905830983	7.882908269	2.50E-139	3.47E-136
TGFA	3.884751697	9.343298433	1.00E-93	5.01E-91
SOD3	3.882931124	0.253503519	3.96E-10	5.66E-09
DRGX	3.881648379	3.07838571	2.67E-27	1.63E-25
HECW1	3.86862747	3.299138869	9.25E-42	1.05E-39

AQP3	3.841448665	5.04910764	4.80E-100	2.86E-97
UBD	3.838014174	-0.776764426	1.82E-07	1.68E-06
CDC42BPG	3.815440702	4.06332509	7.55E-43	9.00E-41
DOK7	3.793399448	1.398870585	1.26E-11	2.11E-10
C6orf99	3.780962056	2.302056503	3.09E-22	1.36E-20
COL17A1	3.772416356	7.524581479	1.08E-18	3.62E-17
ENTPD8	3.731514204	1.419594219	1.21E-18	4.04E-17
FAM25A	3.728575931	-0.28107362	9.91E-09	1.14E-07
KHDC1L	3.713351581	1.66679754	5.43E-19	1.87E-17
YPEL4	3.690411985	3.270828769	3.96E-26	2.23E-24
PLA2G4D	3.640082229	2.757825488	2.63E-26	1.50E-24
RUBCNL	3.633092003	1.463393154	2.18E-17	6.44E-16
SUSD2	3.604856713	1.290413469	4.36E-07	3.70E-06
NCF2	3.600271809	5.721943903	1.69E-68	3.93E-66
DPP4	3.598225013	3.085287132	3.02E-16	7.94E-15
KRT17	3.524937056	3.994194304	1.50E-18	4.95E-17
MALL	3.510261335	4.22830335	2.56E-33	2.04E-31
GPER1	3.502005233	2.335503545	1.17E-22	5.34E-21
CD24	3.481927475	2.112241052	5.94E-22	2.56E-20
CYP26B1	3.480508806	7.120604913	1.09E-43	1.34E-41
NXNL2	3.478024346	2.107779879	1.17E-20	4.67E-19
CLEC4O	3.460711531	-1.978107743	0.00195938	0.006844163
FGD3	3.442895759	1.089802706	2.00E-13	4.00E-12
CD52	3.441982833	-1.427609038	0.00186157	0.006548187
CDH2	3.422807194	4.31981735	1.12E-44	1.42E-42
DAPP1	3.42119794	-1.478213874	0.000229363	0.001058386
CEMIP	3.403212684	7.975925901	5.72E-67	1.30E-64
CRISPLD2	3.393725415	6.528600197	8.49E-126	7.59E-123
MYH15	3.38957344	-0.193443417	0.000278176	0.001254261
CD34	3.37151549	5.182866012	2.11E-74	5.38E-72
ITGB2	3.366536619	3.404922496	2.85E-33	2.26E-31
TTLL6	3.358240868	1.513232756	6.14E-10	8.56E-09
RGS9	3.355892559	3.656314894	3.22E-42	3.77E-40
LRG1	3.353782064	2.517231418	4.89E-11	7.79E-10
TTC9	3.333871484	1.778846195	2.38E-18	7.69E-17
CD300C	3.32406588	2.444785108	5.41E-26	3.02E-24
FMOD	3.318648249	2.371956827	1.42E-06	1.09E-05
LINC02762	3.294601039	1.616052748	2.17E-12	3.92E-11
HSPB8	3.290087758	8.220258376	2.09E-71	5.13E-69
MAOA	3.289288578	6.801576756	5.12E-81	1.64E-78
DEFB1	3.284309683	-1.556757805	0.000337698	0.001485991
MIR503	3.270329688	-1.585104739	0.00612977	0.018197437
FABP3	3.270281801	2.000324123	4.98E-20	1.86E-18
FAM83A	3.259766513	5.307578808	5.78E-30	3.96E-28
TTC22	3.237679085	1.610988709	8.80E-18	2.66E-16
GSN	3.231848053	8.746972235	5.96E-82	1.96E-79
LANCL3	3.195230825	1.570518301	5.18E-14	1.09E-12
EDN2	3.186994601	4.005739883	1.41E-10	2.14E-09
PDK4	3.185109489	1.139880421	8.36E-10	1.15E-08
H19	3.182465714	-0.325678983	4.14E-06	2.88E-05
ITGB8	3.175077539	2.233662935	4.29E-15	1.01E-13
SERPINB6	3.171194944	9.781426919	7.63E-131	8.68E-128

SLC22A1	3.168342892	0.701325557	3.06E-11	4.96E-10
PAPSS2	3.163255336	9.52785111	1.22E-65	2.67E-63
HAS3	3.144439691	3.476018111	1.99E-20	7.72E-19
DCST2	3.138339818	0.963774367	6.01E-09	7.23E-08
CPN2	3.119345492	0.184376703	2.31E-09	2.94E-08
ADAMTSL5	3.109459429	5.616774418	1.37E-41	1.53E-39
GPR132	3.088739169	2.871629181	4.72E-15	1.11E-13
CPM	3.078057836	4.168893863	8.34E-25	4.28E-23
PRR16	3.077823236	2.521063562	1.21E-20	4.81E-19
TGM1	3.075094758	2.200405269	6.46E-22	2.77E-20
PROS1	3.067199805	2.523637786	2.99E-07	2.67E-06
BEST1	3.04822533	1.572362043	9.25E-17	2.57E-15
CLIC5	3.034953265	2.606740994	3.38E-16	8.85E-15
DSEL	3.002840357	3.351821437	1.62E-15	3.96E-14
SMAD9	2.999944155	3.147932282	8.66E-11	1.35E-09
SEC14L6	2.991493069	2.645660703	3.95E-22	1.72E-20
IL2RG	2.985573266	1.470730826	8.07E-10	1.11E-08
TUBA3D	2.985229176	1.749096256	2.00E-17	5.91E-16
TNFSF14	2.961769605	0.242186738	7.09E-06	4.70E-05
IGFBP4	2.944787822	12.16396203	1.60E-108	1.11E-105
CISH	2.944500339	3.001791658	8.95E-17	2.50E-15
SEMA3A	2.92935635	2.101777962	1.02E-16	2.81E-15
CHI3L2	2.913314588	0.322462187	1.21E-09	1.60E-08
ESR2	2.908312278	9.269991581	3.54E-15	8.41E-14
TBXA2R	2.903131153	3.889239746	4.56E-25	2.38E-23
ERVV-2	2.898702594	0.209027748	1.20E-08	1.36E-07
TCN1	2.883946956	2.941458799	6.66E-14	1.39E-12
GNGT2	2.86805349	1.423499731	1.64E-13	3.33E-12
OASL	2.867893265	5.922180411	8.31E-64	1.68E-61
EPHA4	2.865955603	0.812943927	4.10E-08	4.26E-07
PSG9	2.847295153	2.333413741	4.14E-09	5.11E-08
CPA4	2.840963582	6.341498815	4.14E-62	7.84E-60
PDGFB	2.824938948	5.190075617	4.12E-44	5.10E-42
ARMH4	2.819114881	2.787798301	1.29E-13	2.64E-12
RHOJ	2.782906279	-0.143072736	0.018942818	0.046894432
IFIT1	2.78259005	4.943185983	1.96E-50	2.88E-48
NBPF4	2.771719683	-1.878416621	0.005880654	0.017582973
PTAFR	2.768694409	2.209448382	1.07E-16	2.94E-15
KRT19	2.765889944	10.40992651	1.21E-58	2.22E-56
CCDC80	2.764502303	6.837317154	8.76E-31	6.30E-29
CCNA1	2.755333046	6.53738966	1.01E-50	1.51E-48
PRPS1L1	2.73685868	1.118716149	0.001426341	0.005213538
HMOX1	2.732037861	7.656164182	2.73E-65	5.70E-63
PSG6	2.725376629	1.804827465	6.63E-05	0.000349241
CRYBG2	2.717639636	4.943737648	3.10E-30	2.16E-28
PHOSPHO1	2.713507251	1.285205482	6.26E-09	7.50E-08
LYPD5	2.70632934	0.684937688	9.36E-07	7.46E-06
CT62	2.70184132	5.212212193	1.90E-12	3.44E-11
KLF17	2.69167063	3.350030238	1.72E-20	6.79E-19
MEGF6	2.68900663	5.858670406	2.87E-12	5.11E-11
FAM222A	2.681379856	3.002094881	3.00E-19	1.05E-17
TPD52L1	2.674486027	3.365143756	6.28E-27	3.67E-25

WNT7B	2.673633881	3.251858469	1.80E-25	9.55E-24
LGALS9B	2.670795162	-0.082802583	1.91E-05	0.000114941
PLAC1	2.663948866	1.706569884	4.78E-10	6.73E-09
CDH3	2.66240079	5.040574196	6.00E-38	6.06E-36
MIR622	2.647436269	1.654749744	0.001206609	0.004517206
LBH	2.643575394	2.369597036	3.76E-20	1.41E-18
ANOS1	2.638408405	6.406587278	5.16E-29	3.35E-27
NMNAT2	2.62952929	3.875360826	1.02E-25	5.59E-24
SAMD9	2.628105096	1.64269223	2.42E-11	3.98E-10
OTUB2	2.61977832	6.381990108	3.75E-76	9.77E-74
TRIM29	2.61779924	1.798655564	4.24E-08	4.39E-07
PAG1	2.601384328	0.323693905	8.16E-08	8.06E-07
MGAM	2.596672158	2.534175738	1.81E-11	2.99E-10
ARRB1	2.59493138	6.632418923	6.19E-78	1.72E-75
IFITM10	2.590995939	3.859368025	2.26E-27	1.39E-25
MARCKSL1	2.583822973	6.521411098	1.62E-84	5.96E-82
NPFFR2	2.582987474	-0.908728754	0.013583529	0.035568333
NXF3	2.577204716	-0.990863318	0.012385139	0.032933209
ZNF488	2.575517868	3.075012684	1.35E-22	6.13E-21
RFPL4A	2.566629076	0.993414617	4.81E-09	5.89E-08
GM2A	2.54768019	7.366198701	9.38E-86	3.79E-83
MIR138-1	2.536949837	-1.43634449	0.017311558	0.043588776
GJB3	2.530560393	6.294471292	6.37E-70	1.50E-67
HSPA2	2.530467461	2.885904589	1.67E-21	6.88E-20
SPANXN3	2.498319263	-0.553731769	0.000103076	0.00051803
SEMA5A	2.49631454	3.696490127	8.29E-15	1.90E-13
AKR1C3	2.495957885	2.448317819	4.41E-14	9.38E-13
PORCN	2.484202507	6.331977694	3.81E-42	4.42E-40
SLC6A9	2.484093576	6.805168946	2.28E-78	6.63E-76
SERPINA1	2.449827148	8.336409933	2.75E-89	1.19E-86
PHETA1	2.449534909	6.123344821	1.03E-65	2.30E-63
IFI27	2.445911058	6.099358698	1.22E-35	1.10E-33
ISG20	2.445022215	5.473284181	5.32E-40	5.60E-38
TSPAN1	2.441380567	1.958288182	7.93E-12	1.36E-10
UPK2	2.421427345	0.233700726	9.87E-05	0.000499068
ZNF219	2.413504742	3.602183911	8.11E-24	4.00E-22
GCKR	2.408133237	0.71088745	4.95E-06	3.40E-05
THBS2	2.407097592	7.952886374	4.99E-46	6.50E-44
KIAA0513	2.401583698	5.839783982	9.05E-54	1.49E-51
SPTBN5	2.394294249	1.968025637	0.00012786	0.000626733
ARL4C	2.387850325	7.561635011	2.38E-76	6.35E-74
NOL4L	2.375057097	4.522464069	1.23E-23	5.99E-22
PRELP	2.363783877	-0.412345299	0.000185997	0.000876692
C6orf15	2.360084332	-0.024332822	2.66E-05	0.00015508
TSPAN15	2.348418132	5.402114431	9.03E-53	1.41E-50
ACER2	2.34150257	1.430550144	0.000424908	0.001821719
S1PR1	2.340715274	1.1474756	9.75E-09	1.12E-07
FN1	2.334877891	12.09389425	9.57E-17	2.66E-15
HKDC1	2.326681581	3.663382338	1.05E-14	2.37E-13
EPB41L1	2.307609728	6.099940138	5.71E-53	9.05E-51
USP18	2.306322789	2.574505947	1.45E-13	2.95E-12
LGALS9C	2.305232409	0.45859563	0.000568005	0.002355366

LY6G6C	2.301560327	-1.123124635	0.006694593	0.019554272
BIRC3	2.29678536	5.859719058	2.26E-14	4.92E-13
ELFN2	2.285124705	6.022608525	1.37E-42	1.61E-40
CRCT1	2.284678188	-1.159402834	0.003258616	0.010552151
PCDHB6	2.278719176	0.14679541	1.64E-06	1.25E-05
REN	2.277872271	0.798987812	9.45E-06	6.10E-05
FBXL7	2.259521165	-0.103506893	6.12E-06	4.13E-05
MISP	2.256831052	2.85382775	3.18E-15	7.58E-14
SHISA2	2.240557835	4.799374258	1.52E-11	2.53E-10
C3	2.238064274	9.442811885	9.70E-27	5.65E-25
B3GNT7	2.236460105	2.390559681	2.17E-09	2.77E-08
RFPL4AL1	2.233118119	0.232301402	0.000291938	0.00130761
DLL4	2.23268805	1.90556789	3.55E-06	2.51E-05
GRIK2	2.231358646	0.639771216	1.72E-07	1.61E-06
PPP1R1C	2.222720695	2.645900872	7.81E-09	9.20E-08
C1QTNF6	2.217753399	5.065972203	4.02E-35	3.50E-33
COL4A4	2.215391175	1.357054873	8.11E-05	0.000417831
ZBTB32	2.21210267	0.20014815	0.008860499	0.024797389
C18orf32	2.20837948	-0.311326666	0.001831693	0.006463067
CSF2RB	2.207661299	1.724325112	6.52E-12	1.13E-10
MMP12	2.203833548	-0.069902311	5.05E-05	0.000275017
PTPRH	2.202250362	6.267506313	3.77E-23	1.79E-21
IFI44	2.199473856	0.738270475	0.002030211	0.007040502
SLC19A3	2.193149906	1.827710305	7.67E-09	9.06E-08
FHL2	2.17560538	9.151857102	3.90E-71	9.38E-69
CTSD	2.155413861	9.413768316	3.79E-54	6.42E-52
SLCO3A1	2.152368991	5.145093564	2.76E-36	2.60E-34
SDK1	2.149860593	3.381326195	3.77E-17	1.09E-15
SLCO2A1	2.14289704	-0.103101579	0.000330675	0.001457134
IRX5	2.142579251	3.977451836	3.70E-26	2.11E-24
SLC1A1	2.136701225	1.367590461	2.23E-07	2.03E-06
CCDC149	2.131673911	1.869844121	1.78E-10	2.69E-09
MIR130A	2.131658957	-1.887064395	0.028962827	0.066947125
TFAP2C	2.128563314	6.234340559	2.46E-43	2.99E-41
SLC9A3R1	2.12733818	8.130998472	3.63E-53	5.82E-51
OSR1	2.127051085	1.981808083	9.76E-13	1.82E-11
SLC25A42	2.121900329	4.295202612	3.88E-27	2.32E-25
ANGPTL2	2.118854746	3.67505809	2.85E-11	4.63E-10
USP41	2.116108278	-0.205825833	3.53E-05	0.000199622
NHSL2	2.111201046	4.024712957	0.002613054	0.008779319
TRPM2	2.110949123	2.991163963	1.72E-16	4.61E-15
RASGRF1	2.109869751	5.040638545	6.90E-33	5.40E-31
PRDM1	2.107391745	2.844409939	4.36E-14	9.28E-13
IGFBP6	2.09774941	4.806335164	3.57E-22	1.56E-20
CDON	2.097267191	3.671673053	9.07E-14	1.88E-12
SCUBE1	2.085944027	-1.699688532	0.045761448	0.097446431
DHRS2	2.081945272	0.965691836	4.81E-05	0.000263518
CARD6	2.07627967	4.459029825	7.49E-25	3.88E-23
FGF11	2.058146732	2.962531895	2.14E-14	4.68E-13
SRPX2	2.055914342	3.179275463	2.26E-10	3.36E-09
PIK3CG	2.050813577	2.831786439	2.90E-10	4.23E-09
CRPPA	2.049509569	1.990195635	8.23E-06	5.39E-05

GLIPR1	2.042833799	5.485087115	5.27E-27	3.09E-25
OAS1	2.03254236	2.245182988	2.85E-09	3.58E-08
B3GNT3	2.02342878	5.71104568	3.06E-45	3.91E-43
STC1	2.01806392	7.360431389	4.55E-15	1.07E-13
HMGN3	2.017134397	1.84095255	1.88E-05	0.000113149
KRT15	2.017078814	4.891548295	3.63E-28	2.29E-26
MMP19	2.012526345	2.773749931	1.08E-05	6.89E-05
CLMP	2.008678159	5.507420404	2.75E-29	1.80E-27
GPR1	2.007294004	4.348618969	6.69E-24	3.32E-22
GIPR	2.006420504	0.957158963	1.36E-07	1.30E-06
TENT5A	2.003386148	1.492099699	3.60E-06	2.54E-05
PODXL	2.000803035	10.0423447	1.63E-29	1.09E-27
LOX	1.997538499	5.975652598	1.62E-10	2.46E-09
GPC1	1.990204819	6.293854744	1.74E-37	1.74E-35
VNN1	1.989150939	0.34810219	0.000583948	0.002412689
TCIRG1	1.986149534	7.083790335	7.31E-16	1.86E-14
DBH-AS1	1.981418048	1.286542705	0.000330295	0.001456104
CCDC85A	1.977768548	1.73548158	6.60E-08	6.65E-07
PLPP3	1.975154334	3.549323011	3.75E-17	1.09E-15
ACOT6	1.965684788	-0.028611024	0.000243201	0.00111362
SERPING1	1.95563583	2.740618459	1.56E-07	1.47E-06
LAMA5	1.942278802	8.131228415	1.14E-15	2.82E-14
IFNL1	1.941872239	-1.061124646	0.002011273	0.00699226
CCL5	1.940526	0.202029897	0.001018356	0.003897521
LRRC15	1.938567927	3.196755768	1.96E-10	2.94E-09
MATK	1.938078837	1.023395819	0.000112562	0.000559416
PTH1R	1.9223587	1.306623254	3.90E-07	3.36E-06
FBLN1	1.918398385	0.127142618	0.00023412	0.001076765
PSG8	1.918031755	0.778522053	6.30E-06	4.23E-05
ACTG2	1.917126141	0.737549105	1.75E-05	0.000106369
CASTOR2	1.910624498	6.272278339	3.23E-27	1.95E-25
C1QTNF2	1.90485269	1.157577346	8.41E-07	6.78E-06
JAZF1	1.896142709	3.333244924	3.64E-13	7.08E-12
KITLG	1.892584036	1.518377982	2.18E-05	0.000129239
CD9	1.891589833	7.820668426	3.12E-49	4.49E-47
FAM102A	1.884887493	6.005279189	1.81E-29	1.20E-27
MYO5B	1.884401344	2.680810553	1.42E-13	2.89E-12
FOLR1	1.880219517	0.604638713	0.000453057	0.001928533
KDR	1.878879213	0.74148898	0.000473896	0.00200497
ADGRD1	1.877320838	4.081742489	5.92E-19	2.03E-17
TNIP3	1.877150247	-1.586716697	0.022877514	0.055003571
RHOD	1.876648214	6.837756906	2.85E-20	1.09E-18
LYPD8	1.87431995	2.312702212	2.87E-08	3.06E-07
DAB2	1.865515005	6.337555824	3.89E-35	3.40E-33
TGM2	1.863992158	12.33915428	1.14E-52	1.76E-50
TSPAN13	1.862034025	3.770354113	3.31E-19	1.15E-17
CPE	1.843733281	1.455793753	7.16E-05	0.000373924
SYNJ2	1.838133624	6.685255792	2.56E-29	1.68E-27
OVOL2	1.837792019	-0.308222597	0.020064854	0.049185806
HIGD1A	1.829068014	6.24970203	3.77E-07	3.27E-06
CASTOR3	1.828512063	4.215251642	1.22E-17	3.66E-16
ZNF114	1.828216655	4.086554945	1.97E-13	3.95E-12

DYNLT3	1.82778679	4.006980633	1.78E-11	2.94E-10
ATP2B4	1.813617677	7.845920315	5.16E-15	1.21E-13
JUP	1.813617286	7.014654224	6.40E-40	6.68E-38
RAET1L	1.812101824	1.316758136	0.000106504	0.000532905
RGS4	1.81205338	3.47889296	1.18E-09	1.57E-08
MLLT11	1.811334146	4.836916865	7.11E-17	2.01E-15
BICDL1	1.810980451	2.668857312	1.26E-10	1.94E-09
MGAT5B	1.809256285	5.209482695	5.33E-28	3.34E-26
SATB1	1.80696001	-0.092721492	0.041217171	0.089211231
FUT8	1.804855008	6.140159999	3.71E-27	2.23E-25
GATA6-AS1	1.797407325	-0.139925911	0.000214368	0.000994783
RNF152	1.796589715	2.103414017	1.91E-09	2.46E-08
CD83	1.793297425	4.823029717	1.12E-21	4.64E-20
ZNF883	1.789826479	-1.831557764	0.038171165	0.083791832
ZMYND10	1.788872773	-0.943465524	0.044835904	0.095768756
P2RX6	1.787968172	0.04113067	0.000511605	0.002147813
TNFRSF11A	1.787772282	0.889846014	3.15E-06	2.26E-05
APOL1	1.781080153	5.41840397	4.29E-27	2.56E-25
ISG15	1.779434524	8.020736577	6.87E-19	2.35E-17
PLCD3	1.776539446	7.11684844	9.81E-35	8.47E-33
DHFR2	1.776194051	0.850999392	0.000101449	0.000511494
SDC2	1.776020039	3.13582059	4.05E-10	5.78E-09
GSTT2B	1.766171608	3.674560351	4.86E-11	7.76E-10
LIPH	1.764375657	3.296020747	1.17E-14	2.64E-13
STS	1.762276592	5.292105508	6.64E-10	9.21E-09
MMP24	1.759712663	4.159545789	2.39E-19	8.44E-18
DBH	1.754558873	-0.297877832	0.04178846	0.09033841
NRG1	1.745675512	6.53194143	1.44E-40	1.57E-38
SERINC2	1.744803849	7.892775555	6.21E-33	4.89E-31
BDKRB1	1.731562696	-1.193679607	0.006199025	0.018355156
ECM1	1.730404639	6.217221608	1.04E-13	2.14E-12
ANXA9	1.725573066	2.923948937	1.80E-09	2.33E-08
TCAF2	1.724490202	3.757040885	4.32E-07	3.67E-06
BMP8B	1.723262654	5.914305046	5.10E-34	4.17E-32
ETV7	1.719812652	1.177054327	2.23E-05	0.000131808
ZNF385A	1.716287923	4.766282651	1.07E-22	4.94E-21
IL1R1	1.711490096	4.171876103	1.38E-10	2.12E-09
SLC46A3	1.702143889	4.737623563	2.85E-22	1.26E-20
CCDC71L	1.701186434	5.758197382	1.27E-36	1.22E-34
C6orf132	1.700520223	8.182031765	1.98E-17	5.86E-16
GPR155	1.694701341	2.478876989	0.000797389	0.003153587
ADAMTSL4	1.693838358	5.23300697	8.94E-08	8.78E-07
MITF	1.689108363	2.351256067	3.67E-05	0.000206708
MINDY1	1.682084407	4.127881113	4.24E-18	1.33E-16
NUAK1	1.68144491	8.147864775	8.78E-32	6.58E-30
SEC14L2	1.680702267	4.994633881	5.16E-26	2.90E-24
SLPI	1.680523411	-0.584566567	0.005952289	0.017758987
MIEF2	1.680084015	5.918055317	1.00E-28	6.47E-27
CCDC68	1.675609223	1.044868683	3.23E-06	2.31E-05
SPIRE2	1.67088782	5.1564232	6.09E-18	1.89E-16
PCDHB9	1.669250818	0.140643612	0.004872628	0.014984138
ADRA1B	1.668976754	2.575086354	5.74E-10	8.03E-09

ADAM19	1.658107619	8.635478074	1.31E-23	6.34E-22
KCTD11	1.657958216	5.760278618	1.10E-35	9.95E-34
GREB1L	1.656663399	5.32406048	1.28E-13	2.62E-12
HPSE	1.655668988	3.920870355	5.63E-17	1.61E-15
TP53I3	1.652883233	2.971144349	2.32E-10	3.43E-09
CLIC3	1.65067708	4.923000197	7.16E-13	1.35E-11
ZNF254	1.649740966	2.922697659	7.29E-08	7.27E-07
TRANK1	1.649176742	3.030828152	5.34E-09	6.48E-08
UNC13A	1.644868375	3.603034051	6.65E-12	1.15E-10
MPPE1	1.640308262	1.476872083	9.63E-07	7.64E-06
PDZK1IP1	1.635858631	2.877861853	4.71E-07	3.97E-06
JAK1	1.633568593	8.547031166	1.36E-19	4.90E-18
ATL1	1.633322838	2.270479036	1.36E-07	1.30E-06
DGAT2	1.633010016	3.915497667	6.04E-13	1.15E-11
PCDHB8	1.631489813	-0.383993034	0.021711325	0.052624216
ARL3	1.626561826	5.734559493	5.44E-13	1.04E-11
ZNF397	1.623995323	1.69710104	0.000561654	0.002331933
SRBD1	1.620904878	3.964689022	1.60E-15	3.94E-14
PPFIA4	1.617045059	2.497675427	0.004481664	0.013928985
CILP2	1.61288453	3.463035769	6.82E-08	6.84E-07
COL5A1	1.609940425	8.338637385	6.74E-11	1.07E-09
PBLD	1.6096696	0.228813505	0.001993342	0.006943418
TMEM102	1.609444975	4.085957855	1.22E-14	2.74E-13
DIXDC1	1.609433871	3.25605421	7.64E-07	6.19E-06
NPPB	1.609413836	6.498381255	5.53E-31	4.00E-29
CEACAM1	1.609322255	1.126762257	9.83E-06	6.30E-05
SLC35F6	1.608770906	7.626206334	1.54E-36	1.47E-34
CD22	1.607435671	3.662325859	1.23E-11	2.07E-10
GINS4	1.60553131	7.06162398	3.12E-40	3.31E-38
CABLES1	1.598877208	6.967653438	6.70E-34	5.44E-32
C1S	1.596148299	1.298622197	0.003188946	0.010366815
CYP27B1	1.594858805	0.89832403	0.001334538	0.004915292
ULBP2	1.590393649	4.865066123	9.95E-23	4.63E-21
CCDC159	1.586069858	0.373710261	0.00216626	0.007460634
MXD1	1.584168916	4.772602078	3.44E-19	1.20E-17
PRRG2	1.580909968	1.910175282	6.25E-06	4.20E-05
RBM47	1.578487335	3.426546185	3.03E-12	5.38E-11
ULK1	1.576693811	6.114090007	2.38E-22	1.06E-20
RASGRP3	1.574936952	0.91594391	0.000298975	0.001334355
BMP1	1.571512719	6.29216298	1.32E-24	6.64E-23
C11orf74	1.571173331	0.335079584	0.002482462	0.008392639
SLC22A23	1.568794562	5.028714035	2.41E-13	4.80E-12
FRRS1	1.568190835	-0.841429862	0.012632743	0.033442444
LGR4	1.565330073	4.368620055	4.30E-08	4.44E-07
SMOX	1.56222549	6.308838196	2.41E-29	1.59E-27
SMAD7	1.560615097	4.409975226	2.14E-15	5.21E-14
CEMIP2	1.546037837	6.736235919	2.47E-10	3.64E-09
CGN	1.543580305	3.304520861	6.82E-06	4.54E-05
TAS1R3	1.542291993	3.779518732	9.85E-09	1.13E-07
IFI6	1.541568454	3.814507317	1.22E-09	1.61E-08
NRXN3	1.539170671	5.67874656	1.00E-09	1.35E-08
FDXR	1.538167513	4.755876469	1.20E-16	3.27E-15

ARSA	1.534683875	2.936505187	1.99E-08	2.16E-07
RGL1	1.528409712	2.352355164	3.84E-07	3.32E-06
IGSF23	1.526468394	0.261303845	0.001449567	0.005279932
JPT1	1.522587184	9.717724214	1.18E-32	9.14E-31
GNPTAB	1.520489761	4.445077413	3.26E-05	0.000185724
PTHLH	1.519184667	1.506096848	0.000102999	0.000517906
SMIM8	1.517801608	0.317449187	0.035281811	0.078539984
CXCL16	1.517605943	4.55708156	1.28E-16	3.49E-15
ERV3-1	1.515842567	2.005701247	2.82E-06	2.04E-05
ACSS1	1.514349319	4.584904108	2.73E-12	4.88E-11
FOXC1	1.513290815	5.308697162	2.79E-23	1.33E-21
CLTB	1.510622987	8.51125646	1.08E-20	4.33E-19
MYO18A	1.505948694	7.026484688	1.01E-23	4.94E-22
TBKBP1	1.501453516	4.115227865	4.94E-08	5.05E-07
SIN3B	1.501051293	7.949565222	1.30E-33	1.04E-31
PAEP	-1.500225478	0.831709525	0.000133362	0.000650901
GALNT13	-1.500454681	0.771848656	0.00018297	0.000865892
IFT74	-1.501940046	0.966906845	0.000115344	0.00057188
LYAR	-1.503742408	4.785936624	9.07E-16	2.28E-14
GSR	-1.50595103	5.129393647	1.39E-19	4.98E-18
C5orf34	-1.507121277	1.378211725	7.73E-05	0.00040037
ZFP69B	-1.510855763	0.980336092	0.002634685	0.008835688
MIF4GD	-1.511764735	4.642404613	6.38E-15	1.48E-13
FAM81A	-1.513281346	1.019935136	3.59E-05	0.000202755
NEK3	-1.514441044	1.868450378	6.79E-07	5.59E-06
SLC7A11	-1.515418399	6.250944175	1.49E-16	4.03E-15
COL13A1	-1.516791888	6.051538714	2.05E-19	7.30E-18
ENPP1	-1.520160949	4.194556322	1.44E-07	1.37E-06
FBXO45	-1.520456072	4.527221277	7.22E-14	1.50E-12
VWA5B2	-1.520943592	0.413511739	0.002882597	0.00953816
CKAP2L	-1.521253484	4.700919276	1.33E-06	1.03E-05
ALPK2	-1.52176908	4.474632916	7.00E-16	1.78E-14
MLH3	-1.522142985	1.754782738	0.000297789	0.001330487
FST	-1.522298231	5.049588538	7.20E-13	1.35E-11
CXCL1	-1.524514929	7.047453191	1.69E-30	1.20E-28
TNFRSF21	-1.524906555	7.01874405	1.90E-13	3.80E-12
CAMK2N1	-1.526630657	4.56723293	1.24E-18	4.13E-17
PDLIM5	-1.5276983	6.130774493	2.57E-13	5.10E-12
IRAK2	-1.527841902	4.588125572	2.44E-15	5.89E-14
MAK16	-1.528112373	3.042765014	2.78E-10	4.08E-09
ROBO4	-1.528818124	6.385600541	5.23E-25	2.72E-23
DUSP2	-1.528936634	0.893150889	0.00011846	0.000585704
TTC9B	-1.532015036	-1.020045974	0.012732203	0.033677257
DHRS3	-1.534967555	1.152704812	0.000278224	0.001254261
PPFIBP2	-1.537239602	0.801467821	0.002124788	0.007331924
TM4SF1	-1.540820162	7.171012281	3.97E-20	1.49E-18
CDC7	-1.547784916	2.001053561	7.49E-05	0.000389814
PLCXD2	-1.547805565	-0.218374577	0.002963885	0.009777305
TRAF5	-1.548618408	2.635171558	3.42E-06	2.43E-05
METTL27	-1.549582878	2.304343273	6.74E-07	5.55E-06
SYNPO	-1.550329886	4.829308025	3.29E-18	1.04E-16
ADCY7	-1.552238086	5.429138379	9.19E-15	2.09E-13

ENO3	-1.553189881	2.220072466	3.97E-06	2.78E-05
ADAP2	-1.553732231	3.073464633	8.02E-08	7.95E-07
IL6	-1.554069881	7.166104625	1.79E-19	6.44E-18
PPP1R32	-1.554584742	-1.299867486	0.028052285	0.065070698
PARD6A	-1.555174931	2.161747489	0.000200239	0.000935719
SYT15	-1.560704021	0.887658359	1.76E-05	0.000106807
SMURF2	-1.563962707	6.161122932	4.87E-15	1.15E-13
EFEMP1	-1.564308092	5.899249883	3.20E-13	6.26E-12
ATP11C	-1.564384195	3.00488974	0.000956743	0.003687645
SH2B2	-1.565699471	1.878990732	3.57E-07	3.11E-06
TFPI2	-1.569522743	4.484246791	1.04E-09	1.39E-08
SCAPER	-1.5710732	2.060410543	2.97E-07	2.65E-06
F2R	-1.573168124	4.135523081	7.38E-16	1.87E-14
GPT2	-1.576100716	5.294857686	2.29E-20	8.80E-19
COL6A3	-1.576918484	2.660624916	0.001131257	0.004261872
TAFA3	-1.579718029	-0.606683619	0.034946317	0.078091055
FOSL1	-1.581147873	8.289559908	2.58E-34	2.14E-32
GADD45G	-1.583021203	-0.379178003	0.003794139	0.012037208
FLRT1	-1.583560449	0.661313214	0.005194607	0.015772433
CIITA	-1.583937899	-0.129767496	0.003168816	0.010312037
PHF10	-1.588753999	4.420005025	1.26E-14	2.82E-13
ZNF239	-1.589258959	1.525850682	0.02782693	0.064691798
SLC43A3	-1.589451881	7.719761641	2.77E-37	2.73E-35
NOX5	-1.592057676	0.136267944	0.000843347	0.003306564
RGS2	-1.59362592	-0.07567419	0.000633752	0.002591946
SIM2	-1.594606357	2.924282823	1.02E-09	1.37E-08
IVNS1ABP	-1.595257766	4.882850182	2.52E-20	9.63E-19
SUPT3H	-1.595656248	2.777426818	3.99E-10	5.70E-09
MACF1	-1.595895355	5.398950882	2.44E-10	3.60E-09
MACF1	-1.595895355	5.398950882	2.44E-10	3.60E-09
UACA	-1.596031765	4.490450027	1.62E-08	1.79E-07
SAPCD2	-1.600276815	5.889836228	5.09E-30	3.50E-28
CNTNAP1	-1.600436117	4.005457773	6.28E-17	1.78E-15
DDN	-1.601199462	1.25574752	0.005799048	0.017376325
KIF13A	-1.601573026	4.895837284	2.88E-12	5.13E-11
SACS	-1.602616131	5.485623804	1.11E-06	8.70E-06
OCLN	-1.603763421	2.766970014	8.70E-09	1.01E-07
BRCA2	-1.604339424	3.638870234	0.001826995	0.00644831
CTNNBIP1	-1.605837194	4.885798042	6.39E-20	2.37E-18
KIAA0586	-1.611479653	2.905567591	2.64E-06	1.93E-05
PLLP	-1.618382863	1.412895113	4.10E-06	2.86E-05
PRKCA	-1.620582965	5.737980477	4.39E-22	1.91E-20
DUS4L	-1.623922971	1.4810982	0.000138077	0.000671817
SLC22A31	-1.625925976	2.325166635	2.99E-08	3.18E-07
BDH1	-1.627733365	5.322855835	2.30E-24	1.15E-22
LRRC75B	-1.628433575	1.968523555	4.12E-08	4.29E-07
NFIA	-1.630762782	2.331401382	2.33E-05	0.000136865
TGFB2	-1.630860619	6.454123602	1.77E-08	1.94E-07
ETS2	-1.631064679	6.401345093	2.48E-32	1.88E-30
ARHGEF2	-1.632838105	7.944939832	3.10E-37	3.03E-35
CA11	-1.633720513	0.300807993	8.79E-05	0.000450091
ZC3H12C	-1.634112029	4.919315898	2.52E-07	2.28E-06

RPS6KA5	-1.634952463	1.043882048	0.003865996	0.012227993
HIST1H2BM	-1.634999456	-0.848228098	0.036308093	0.080378606
TMEM268	-1.637041092	4.310828278	2.76E-18	8.83E-17
GEMIN2	-1.637424946	1.812758164	0.001791789	0.006337651
AGTPBP1	-1.637548556	3.166910836	9.16E-05	0.000466994
HS3ST3B1	-1.640187772	5.711931559	7.70E-26	4.25E-24
PKDCC	-1.644617121	0.683240323	0.003713107	0.01182508
RUNX3	-1.648229605	-0.873382108	0.010672459	0.029051644
RCOR2	-1.64850149	-0.310029511	0.005016107	0.015349949
MACROD1	-1.649815716	4.719675362	1.22E-20	4.85E-19
LMO4	-1.651109184	3.79402279	1.76E-08	1.93E-07
KCNJ4	-1.655081931	1.230252078	0.000126865	0.000622343
NAT8L	-1.658010918	2.604754959	1.10E-08	1.25E-07
GLI3	-1.660845689	3.037324748	1.23E-09	1.62E-08
KIF7	-1.661791762	2.857988328	3.26E-10	4.71E-09
ROR1	-1.66732379	3.916987913	3.43E-09	4.26E-08
NLRP12	-1.668764172	1.3624146	0.000118842	0.000587126
EPAS1	-1.668898235	6.605621546	2.28E-31	1.66E-29
LONRF1	-1.67010266	2.678980082	8.13E-09	9.52E-08
TFPI	-1.674549916	4.536306473	4.98E-21	2.01E-19
SCARF2	-1.674986965	2.506686799	3.02E-06	2.18E-05
THOC6	-1.678496267	4.686402797	6.28E-15	1.45E-13
FAM72D	-1.682300945	4.271760583	1.19E-09	1.59E-08
FAM72B	-1.684486129	4.569144038	2.16E-22	9.68E-21
NOTCH1	-1.686184172	5.974067653	5.84E-18	1.82E-16
CAMK1D	-1.686868748	1.03971793	0.003155188	0.010281051
MCIDAS	-1.690208692	0.996383764	3.31E-05	0.000188248
CEP290	-1.691366482	1.035907332	0.005716951	0.017154964
SNX30	-1.692824182	4.128128438	1.18E-12	2.18E-11
TFAP4	-1.695252415	3.886578943	5.65E-10	7.91E-09
FAM72A	-1.696744155	4.495961054	7.75E-20	2.86E-18
FOSB	-1.696946472	2.207656424	9.30E-09	1.08E-07
ILDR2	-1.697187361	1.075743253	0.009489937	0.026307468
CLDN4	-1.697405727	7.310627159	2.32E-27	1.43E-25
PTP4A3	-1.697526395	4.884661238	2.48E-16	6.61E-15
SLC19A2	-1.700548584	2.789365165	8.96E-10	1.22E-08
TXNIP	-1.701699338	7.269509614	1.43E-34	1.21E-32
XPOT	-1.703441415	6.147173315	4.96E-19	1.71E-17
FKBP10	-1.705311903	0.022227036	0.003091287	0.010109117
OLFML2A	-1.707055667	2.140430314	6.40E-08	6.46E-07
B3GNT5	-1.712641673	3.017763416	7.84E-12	1.35E-10
SESN2	-1.712691221	5.134648571	1.42E-30	1.02E-28
SGK1	-1.71492969	5.736980009	6.95E-22	2.96E-20
DOP1B	-1.717496276	5.098271937	5.28E-15	1.23E-13
ATP2B1	-1.719975988	5.881381797	1.90E-20	7.39E-19
ABLIM1	-1.723982649	5.132085621	2.59E-23	1.24E-21
TMEM221	-1.725790533	-0.552670564	0.001460239	0.005313906
SPTBN2	-1.725811714	5.907711268	4.54E-36	4.24E-34
PDE8A	-1.726323586	4.124430301	1.91E-14	4.21E-13
RCN1	-1.7267871	7.453958052	4.83E-49	6.87E-47
CENPF	-1.727129706	5.849705792	0.000400205	0.001728838
ALDH6A1	-1.728638185	3.416342264	9.55E-13	1.78E-11

IL1RAPL1	-1.731811743	2.364075375	6.19E-05	0.000328702
GFPT2	-1.734232391	5.305296631	1.12E-19	4.09E-18
ARG2	-1.734375637	3.221196605	8.25E-08	8.14E-07
ESF1	-1.735035809	2.227756177	7.41E-06	4.89E-05
THUMPD1	-1.741939705	2.525033481	2.26E-06	1.67E-05
FUCA1	-1.742005588	3.657519341	2.94E-16	7.75E-15
GAL	-1.746958235	2.519521494	1.84E-09	2.37E-08
PER1	-1.750554855	3.219685423	1.86E-09	2.39E-08
ZFP1	-1.750681476	1.214293375	0.000977507	0.003763041
SMAD3	-1.751992034	7.063380716	5.97E-39	6.13E-37
BEND7	-1.757327751	3.996694017	1.77E-06	1.34E-05
LIFR	-1.758990981	3.177384992	0.001737952	0.006175821
CFP	-1.760619354	1.091380031	2.77E-06	2.01E-05
EEPD1	-1.760955991	2.355204764	4.06E-09	5.02E-08
SERPINE1	-1.767469554	10.01924905	1.99E-37	1.97E-35
PCK2	-1.76785465	6.430936641	2.09E-32	1.61E-30
FMNL2	-1.768400442	4.124530463	9.03E-10	1.23E-08
ERVMER34-1	-1.78716372	0.784443056	3.44E-05	0.000195105
TSPAN12	-1.787710253	0.429927303	9.44E-05	0.000479339
TCEA3	-1.789735375	3.952778146	2.04E-14	4.49E-13
INHBE	-1.795289869	-1.182722162	0.008258147	0.023331921
EGFR	-1.798152266	7.377272612	2.33E-26	1.34E-24
RAB38	-1.804195591	-1.434616142	0.008768722	0.024574799
FAM160A1	-1.804884763	3.455467499	9.10E-13	1.70E-11
TUBE1	-1.808536081	2.141557343	1.11E-07	1.07E-06
ODC1	-1.810231413	7.221920089	2.60E-19	9.15E-18
BCL2	-1.811392153	1.753979413	3.00E-07	2.67E-06
ADRB2	-1.81234376	3.896850512	2.79E-16	7.38E-15
FOXA2	-1.8146346	0.949936692	4.46E-05	0.000246306
N4BP3	-1.817660986	0.610651683	8.82E-05	0.000451146
NXPH4	-1.818671877	2.351795231	4.80E-09	5.88E-08
CRADD	-1.820366849	4.289250763	3.08E-15	7.37E-14
SLIT2	-1.824810086	4.809367277	4.89E-22	2.12E-20
FAM135A	-1.829238581	1.924325471	2.16E-06	1.60E-05
FAM72C	-1.829999954	4.522956745	8.98E-25	4.59E-23
RHOV	-1.830762651	0.713160984	1.68E-05	0.000102756
CITED4	-1.832104811	-0.26474077	0.001760297	0.006242822
PLD1	-1.838097914	3.586752587	1.23E-11	2.07E-10
MERTK	-1.839559842	1.625037594	0.013050865	0.034399768
CLIP2	-1.840623456	4.876358658	4.28E-18	1.34E-16
TRABD2A	-1.843270025	0.662353606	7.72E-06	5.08E-05
KRTAP2-3	-1.844057736	2.727727972	1.93E-10	2.91E-09
GPR162	-1.846562567	0.210464936	0.000221137	0.001023223
ZNF697	-1.848626467	4.840480722	3.23E-12	5.72E-11
ARAP3	-1.849722627	5.7163872	1.38E-35	1.24E-33
LAMP3	-1.85031652	4.438122082	2.51E-23	1.21E-21
ADORA2B	-1.851141262	5.720736916	2.71E-13	5.36E-12
SAMD11	-1.852604806	1.653852636	1.14E-07	1.10E-06
EREG	-1.852918931	6.413940689	2.67E-19	9.36E-18
KLF2	-1.853604581	2.258945856	1.07E-09	1.43E-08
MYLK	-1.857363566	5.494056266	1.03E-15	2.56E-14
FRMD4A	-1.85775514	4.055557754	2.28E-15	5.52E-14

PPARA	-1.861266908	4.209229357	1.07E-22	4.94E-21
HECW2	-1.863230169	1.496385035	3.73E-05	0.000209314
FAM20C	-1.864011675	4.665880558	7.10E-28	4.42E-26
TCF7L1	-1.865662878	0.740320906	0.006217099	0.018399969
SEMA3F	-1.8688043	2.209983761	1.78E-11	2.94E-10
KDM7A	-1.871530949	2.80702077	2.47E-08	2.65E-07
AC104971.1	-1.879564918	-1.020652365	0.003858282	0.012206681
NPAS2	-1.881757622	5.431932051	2.13E-20	8.22E-19
NRTN	-1.881907641	0.313263181	5.37E-05	0.000290383
FUT1	-1.883919298	4.571179219	9.25E-16	2.32E-14
AC007405.3	-1.884632789	1.148630112	0.008576525	0.024090087
ERICH2	-1.884632789	1.148630112	0.008576525	0.024090087
FAM221A	-1.889398011	0.984362005	0.000458497	0.001949042
POU2F2	-1.891090649	4.228013427	1.44E-08	1.60E-07
CSF2	-1.894022874	7.081631107	1.71E-46	2.31E-44
ARHGEF4	-1.899480777	4.047727296	1.80E-22	8.12E-21
AMN	-1.902398905	-0.518874174	0.000463163	0.001966204
STK17B	-1.904182761	3.032720948	9.75E-12	1.65E-10
TNFRSF1B	-1.904397601	4.288837403	2.59E-25	1.37E-23
DUSP9	-1.90456368	0.87262729	5.59E-07	4.66E-06
IL37	-1.905759143	-1.092121406	0.0056823	0.017075543
GKAP1	-1.918538179	0.623152579	2.97E-05	0.000170386
GYPC	-1.918709434	3.082912197	1.37E-06	1.06E-05
TBC1D8	-1.924131923	2.466315233	1.06E-05	6.75E-05
CFAP45	-1.926726251	0.318924467	7.52E-05	0.000390916
E2F7	-1.927396201	5.248284392	7.56E-26	4.19E-24
SH2D2A	-1.928032895	2.513962092	1.14E-10	1.76E-09
JAG1	-1.932076137	4.206200517	6.69E-09	7.96E-08
DUSP5	-1.934869395	7.117359089	7.45E-24	3.69E-22
ZEB1	-1.936473401	2.709431282	3.08E-12	5.46E-11
TXK	-1.93918057	-1.502840625	0.01431998	0.037224218
GJB2	-1.94113668	0.694523257	5.38E-05	0.00029071
HTR1D	-1.951823564	2.236161342	2.85E-06	2.06E-05
MLXIPL	-1.952026285	0.318355317	9.74E-06	6.26E-05
TNC	-1.954361423	7.18606657	2.29E-22	1.02E-20
TSPOAP1	-1.960700228	3.410848353	5.74E-17	1.64E-15
SLC37A2	-1.964901306	4.226683958	2.02E-17	5.98E-16
SLC43A1	-1.974338233	4.401512753	3.15E-27	1.92E-25
RUNX2	-1.976846678	3.503832195	1.40E-09	1.84E-08
CAPN11	-1.976984812	-1.657077442	0.015090193	0.038783437
BBC3	-1.980975653	2.671268961	5.96E-10	8.32E-09
HLX	-1.9810281	1.154523764	1.02E-07	9.96E-07
DLG4	-2.002682232	2.695047354	9.11E-09	1.06E-07
LRP4	-2.008298289	3.066635618	2.93E-15	7.02E-14
EN2	-2.018534478	1.525372277	6.39E-06	4.29E-05
PCCA	-2.020208273	-0.026047917	0.005136662	0.015623391
PLAU	-2.022360953	7.916131614	1.53E-21	6.34E-20
ADGRA2	-2.032435566	2.167755893	6.86E-06	4.56E-05
LRP3	-2.055251002	3.105823811	5.66E-14	1.19E-12
KRTAP2-4	-2.055868264	-0.570929895	0.000533253	0.00222898
HTR7	-2.057210882	2.628681394	2.92E-14	6.31E-13
ADAM11	-2.065592775	2.376487508	1.46E-13	2.96E-12

CEBPA	-2.068191922	0.832553451	1.97E-06	1.48E-05
UGCG	-2.068699927	6.667223247	7.58E-36	6.98E-34
ADRA2C	-2.072396404	-0.00036637	0.000175802	0.000835561
UCP2	-2.075076083	5.952137716	8.52E-32	6.42E-30
APLN	-2.075960808	3.186558875	1.02E-07	9.95E-07
ANKRD33B	-2.080124756	4.506268738	1.53E-29	1.03E-27
MMP3	-2.0810422	4.593238535	9.74E-16	2.44E-14
P3H3	-2.083727566	3.649638348	1.35E-14	3.00E-13
WNT10B	-2.089504185	1.58306041	4.48E-08	4.61E-07
PLXND1	-2.095478877	5.370962641	1.58E-40	1.71E-38
RBPMS2	-2.099579017	3.034282046	8.64E-15	1.97E-13
ADAMTSL1	-2.10046197	3.616407946	7.20E-05	0.000376114
TMEM52	-2.109797484	2.276042948	6.36E-09	7.59E-08
LIMCH1	-2.119820313	7.178808406	1.94E-46	2.58E-44
DNER	-2.125006057	0.372174153	5.41E-06	3.69E-05
NNMT	-2.127061202	7.247857637	1.86E-31	1.37E-29
FAM167A	-2.14051504	2.003606089	9.56E-12	1.62E-10
RAB3IL1	-2.14759511	4.202333022	1.25E-24	6.30E-23
RELN	-2.150601065	2.715676552	6.14E-16	1.57E-14
HK2	-2.151202946	5.717756685	5.64E-26	3.14E-24
MYCL	-2.163743996	2.087326728	4.80E-12	8.40E-11
SOCS2	-2.168796681	1.927406425	1.04E-06	8.23E-06
SOCS1	-2.170600358	0.379270887	3.85E-05	0.000215214
ATF3	-2.175876258	4.993999854	1.35E-41	1.53E-39
GRAMD2A	-2.185149326	0.976736621	1.51E-05	9.29E-05
NRARP	-2.186188882	1.50717767	2.30E-08	2.48E-07
COL8A1	-2.195619479	7.757826059	7.06E-36	6.55E-34
TENM2	-2.202373221	7.400435696	2.14E-20	8.24E-19
SMAD6	-2.203021536	-0.2368583	0.000508891	0.002138574
MRC2	-2.206525593	4.749572004	1.39E-13	2.83E-12
DOCK11	-2.207144973	0.546675363	0.00027337	0.00123594
PID1	-2.207348875	2.402130613	1.72E-05	0.000104512
PSAT1	-2.231217124	6.942193888	8.14E-22	3.43E-20
GPR65	-2.231822342	3.035381041	3.40E-06	2.42E-05
FAM9B	-2.232407761	0.070266449	0.007614311	0.021783264
CNIH3	-2.234833475	2.450559672	2.21E-05	0.000130507
NR4A2	-2.237079699	0.738402286	8.57E-07	6.89E-06
SOWAHD	-2.258228596	-0.863880772	0.000459233	0.001951505
PTGES	-2.259669672	2.285818612	2.76E-09	3.49E-08
RTN4RL2	-2.259690571	1.837176754	2.49E-12	4.47E-11
NT5M	-2.271029449	1.725713702	8.26E-12	1.41E-10
MMP1	-2.281549607	6.846821158	1.57E-64	3.21E-62
CXCL2	-2.282999838	5.650671005	8.04E-36	7.35E-34
ATP6V0D2	-2.300325112	0.872684611	4.56E-06	3.16E-05
GPR68	-2.309171332	2.58674187	2.86E-11	4.66E-10
PEAR1	-2.310118444	3.09946502	1.06E-10	1.65E-09
HOXB9	-2.311695138	5.665189452	1.36E-31	1.01E-29
SLC1A3	-2.316552898	1.603695349	1.44E-12	2.64E-11
CYTIP	-2.317173871	2.269161631	5.65E-15	1.31E-13
EEF1A2	-2.341623131	5.501388672	1.19E-34	1.01E-32
CD82	-2.342839241	6.111749205	1.14E-53	1.85E-51
CLDN1	-2.376702641	2.056894139	2.25E-14	4.90E-13

ADM2	-2.382262574	3.336931537	7.43E-22	3.15E-20
SLC12A7	-2.382273586	6.337153728	1.28E-63	2.55E-61
CNKSR3	-2.396742846	2.833405434	9.18E-19	3.10E-17
PDE7B	-2.398580126	1.510837041	6.53E-11	1.03E-09
DDIT3	-2.404619476	4.751646503	4.60E-41	5.09E-39
LAMA4	-2.416096751	1.035940259	7.27E-07	5.93E-06
DEPDC7	-2.428779889	1.745764437	5.97E-10	8.32E-09
NGFR	-2.428801072	-1.20909858	0.000663171	0.002694655
CXCL3	-2.430160359	2.802493425	1.61E-18	5.29E-17
CLMN	-2.447767381	4.413956135	1.52E-29	1.03E-27
EPB41L4B	-2.450106152	2.772423262	1.81E-18	5.93E-17
PRKCE	-2.463229826	5.647415528	4.37E-27	2.59E-25
PYY	-2.464406241	-0.608170744	7.91E-06	5.19E-05
PTPRU	-2.469671873	5.088623528	7.31E-42	8.40E-40
HHIP	-2.473036456	2.669486746	1.28E-12	2.36E-11
MPP4	-2.493750845	1.638430971	1.54E-12	2.79E-11
CRABP2	-2.526816181	2.617956891	6.31E-14	1.32E-12
KCNQ4	-2.542447464	3.22965247	6.99E-19	2.38E-17
TMCC3	-2.543313102	2.228134829	7.27E-05	0.000378867
DCLK1	-2.556679915	1.384815841	1.67E-07	1.56E-06
PRG2	-2.570877639	-0.603560626	0.007118024	0.020613125
CSPG4	-2.578153331	2.661113814	4.29E-18	1.34E-16
NFIB	-2.591509886	3.131899468	1.11E-08	1.27E-07
TOX2	-2.602080278	2.709999442	4.55E-21	1.84E-19
KIF25	-2.603392161	-1.501464081	0.001260936	0.004682783
GCNA	-2.612558029	0.194071162	1.58E-05	9.66E-05
NPR1	-2.615267224	-0.185939248	3.11E-07	2.77E-06
CLDN3	-2.617732669	0.96186518	1.38E-08	1.54E-07
CES3	-2.620743945	0.537085026	4.50E-07	3.81E-06
EXPH5	-2.626865612	3.14559275	3.33E-07	2.92E-06
TEX48	-2.644279041	-0.456633267	0.00013642	0.000664531
SERTAD4	-2.647604159	2.460611947	8.70E-15	1.98E-13
CHST6	-2.671310808	2.235047912	2.93E-15	7.02E-14
RAP1GAP2	-2.672674343	5.395187505	7.94E-49	1.12E-46
BATF3	-2.698889974	3.240363111	8.15E-23	3.81E-21
TNFSF15	-2.699518311	1.808929852	1.56E-16	4.21E-15
FAT3	-2.700370692	2.63866277	1.01E-17	3.06E-16
HGFAC	-2.703612158	-2.020213068	0.01725389	0.043461056
TRIB3	-2.70898688	7.380744441	1.03E-82	3.48E-80
MCTP1	-2.709017116	0.946152245	1.58E-11	2.63E-10
TMEM88	-2.717450923	-1.208359208	0.000266564	0.001209976
PDE4D	-2.718226156	2.578699768	3.16E-08	3.33E-07
CDKN1C	-2.720810611	1.697300638	1.33E-14	2.96E-13
JDP2	-2.733787695	3.456441115	3.80E-30	2.63E-28
CXCL8	-2.808801718	8.191044176	5.49E-20	2.04E-18
KRT86	-2.821808111	0.919690325	6.75E-10	9.36E-09
PALM	-2.834001433	-1.121157106	0.00014143	0.000685731
PDGFRB	-2.835492397	3.153476548	1.10E-28	7.07E-27
RNF43	-2.851823078	3.478705369	1.58E-29	1.06E-27
SERPINB2	-2.869695133	5.85316225	8.00E-18	2.44E-16
IL4I1	-2.878658553	-0.732977565	2.18E-06	1.61E-05
KIF21B	-2.879857465	2.947866351	1.15E-12	2.13E-11

AASS	-2.880703099	3.1936986	2.29E-19	8.12E-18
HES7	-2.908504399	1.734530127	2.58E-14	5.62E-13
HTRA1	-2.934788932	2.872247752	3.00E-20	1.14E-18
BMPER	-2.945333213	2.474741693	2.01E-11	3.32E-10
PHGDH	-2.965218072	2.41037007	4.89E-17	1.41E-15
SCN5A	-2.967963955	4.517805566	4.86E-47	6.69E-45
LARGE2	-2.97761211	1.345675861	2.24E-15	5.46E-14
SLC7A2	-2.989194748	3.755196795	1.19E-19	4.33E-18
MPZL2	-2.992721321	4.709370035	8.21E-25	4.23E-23
MKX	-2.999311966	0.21355071	5.65E-09	6.81E-08
GEM	-3.013582761	2.827856645	1.71E-25	9.10E-24
RRAD	-3.024094365	4.000399981	1.34E-25	7.24E-24
AZU1	-3.030905573	-2.306584403	0.025966949	0.061119121
TLE2	-3.034537141	-0.988457358	2.04E-05	0.000121721
ASNS	-3.066564557	7.11164122	1.07E-32	8.36E-31
PLPP4	-3.148390696	1.958535526	1.22E-19	4.42E-18
IL1A	-3.194170287	6.077764437	2.76E-38	2.81E-36
LTF	-3.23806012	-0.837004175	1.49E-05	9.21E-05
GRAMD1B	-3.282735832	5.626765849	2.21E-94	1.15E-91
SPNS3	-3.295252514	-0.584673872	2.06E-05	0.00012265
DDIT4	-3.377955335	6.456421059	1.43E-35	1.27E-33
NIBAN1	-3.396317488	5.279509951	1.18E-85	4.63E-83
FHL1	-3.404919247	2.470429234	1.37E-18	4.57E-17
COLEC10	-3.459035938	-0.271302511	4.16E-09	5.13E-08
LCN2	-3.529594994	6.241588948	3.52E-98	1.92E-95
CST7	-3.538806786	2.776742814	2.18E-21	8.96E-20
AZGP1	-3.550700737	-1.14812595	1.16E-05	7.32E-05
DMBT1	-3.564657537	3.116222485	6.23E-16	1.59E-14
TBL1X	-3.569311678	6.086820645	7.91E-93	3.81E-90
RTL3	-3.589738067	-1.118978134	3.42E-06	2.43E-05
NRP2	-3.636870688	3.659216139	1.60E-18	5.27E-17
MPZ	-3.650751014	-1.465450091	8.01E-05	0.000413437
GABRE	-3.670070772	0.887447494	1.20E-15	2.95E-14
HAS2	-3.70427853	3.278016688	6.19E-11	9.81E-10
KRT83	-3.807131256	-1.365669432	6.84E-05	0.000358288
KRT81	-3.820029795	4.501231154	1.30E-65	2.80E-63
KLF15	-3.869442831	1.07165059	8.76E-16	2.21E-14
AKAP12	-3.923076324	4.651042213	7.63E-46	9.85E-44
G0S2	-3.981527346	6.685389583	1.79E-136	2.24E-133
IL11	-4.242937106	7.408015889	6.65E-165	2.78E-161
CSF3	-4.297901487	3.384121143	2.23E-46	2.93E-44
IL1B	-4.373865858	7.805527854	2.12E-61	3.95E-59
MIR142	-4.539880897	-2.507688407	0.017264166	0.043478191
IL24	-4.541230418	5.770558713	2.37E-123	1.98E-120
PAPPA	-4.594546279	2.11281044	2.02E-30	1.42E-28
LAIR1	-4.630699703	0.315833774	6.11E-14	1.28E-12
TCIM	-4.864127907	-2.367440635	0.005159912	0.015682676
NUPR1	-4.931426325	0.565777339	2.88E-18	9.18E-17
CXCL5	-6.35815513	-1.464315673	5.48E-07	4.58E-06
GDF15	-7.687383081	1.312875993	1.27E-28	8.13E-27

Supplemental Table 2. Genes significantly regulated following 3 hours of 20ng/mL TNFα treatment of ERβ expressing MDA-MB-231 cells.

Gene	logFC	logCPM	PValue	FDR
AARSD1	5.710330464	-0.212146363	0.000226146	0.000625385
TNF	5.366096685	4.19028799	2.85E-92	2.55E-89
MIR622	5.127226298	3.942466417	2.14E-25	1.51E-23
MEFV	4.925572321	1.279891893	1.15E-36	1.94E-34
LTB	4.479642966	7.142330726	6.01E-55	2.21E-52
DPYSL3	4.365371507	-2.731776514	0.019515282	0.034867395
KRTAP9-1	4.311245898	-2.733753924	0.039346043	0.065008989
NHSL2	3.917534888	5.626744538	4.95E-25	3.33E-23
PTTG2	3.916854884	2.263719341	1.18E-13	1.71E-12
NACA2	3.889514868	5.612481557	1.83E-37	3.36E-35
EFNA1	3.873809784	8.038342505	3.65E-130	6.52E-127
ASB2	3.751444801	3.880684825	6.73E-76	4.21E-73
HDGFL1	3.690212945	-0.809751395	1.91E-07	9.54E-07
SLC34A2	3.595359949	1.180700646	3.92E-17	9.63E-16
SERPINA3	3.411844949	2.369637514	5.26E-17	1.26E-15
ZNF534	3.39172502	2.586975841	1.06E-08	6.70E-08
EDARADD	3.390744519	9.46424827	3.66E-22	1.73E-20
GALNT9	3.38429053	1.148943798	5.07E-24	3.08E-22
CCL20	3.355823125	0.79337847	2.12E-18	6.19E-17
CCL2	3.338825816	-0.086088127	7.42E-10	5.77E-09
IRF1	3.336359057	7.615950353	9.65E-195	3.02E-191
TRAF1	3.334070442	8.145605432	1.12E-198	4.69E-195
CSNK1A1L	3.299800902	1.929673917	2.40E-19	7.95E-18
PCDHB5	3.292433937	0.442269808	1.64E-08	1.00E-07
TCIM	3.270914474	0.291044685	1.19E-11	1.25E-10
MGAT3	3.255750428	-0.869101837	1.53E-05	5.36E-05
F2RL3	3.240192225	2.223384402	1.27E-25	9.26E-24
CACNA2D4	3.196153868	1.943955458	5.00E-31	5.54E-29
FAM71D	3.134749305	2.770084561	8.64E-08	4.63E-07
SCLY	3.126330104	0.307504626	6.18E-07	2.81E-06
GSPT2	3.053140633	2.159493319	5.78E-14	8.84E-13
PCDHB6	3.050887035	0.868288663	1.51E-15	2.96E-14
RNF223	3.046572609	2.248052726	1.01E-34	1.50E-32
PRKACG	2.991916196	0.968703722	2.58E-09	1.83E-08
MAGEA10	2.966841516	1.125556101	2.55E-07	1.24E-06
CSF1	2.951446599	8.642619326	1.61E-236	2.01E-232
NPFFR2	2.950925416	-0.588364131	1.32E-06	5.61E-06
BDKRB1	2.880863217	-0.312485526	1.04E-09	7.91E-09
PANDAR	2.852651658	3.567348682	7.66E-13	9.68E-12
CPZ	2.847455036	-1.141829412	8.08E-06	2.99E-05
TNFAIP2	2.845502792	9.795593711	2.69E-208	1.69E-204
PABPC3	2.830642594	8.570778957	4.21E-34	5.99E-32
SOD3	2.830551491	-0.490426585	4.96E-05	0.000157324
ANP32C	2.797193157	2.899535065	4.82E-13	6.34E-12
PLPP3	2.788107322	4.252992539	2.10E-54	7.52E-52
PCDHB8	2.745005154	0.539666767	7.45E-12	8.06E-11
C11orf98	2.66753891	1.842803886	5.88E-05	0.000183599
PRPS1L1	2.666982632	1.139715662	8.94E-10	6.84E-09

OVCA2	2.601470649	2.069389881	3.82E-08	2.17E-07
FGFBP1	2.586741873	-1.997900062	0.006005071	0.012178437
ELF3	2.50541651	4.416109962	1.39E-68	6.97E-66
CXCL3	2.499696093	5.297371608	2.00E-57	8.65E-55
DHRS3	2.481321443	3.450542836	1.18E-31	1.36E-29
CSF3	2.471619769	6.028034985	2.64E-118	3.67E-115
PADI4	2.465889563	1.402565832	7.21E-16	1.49E-14
GDF15	2.453303814	4.0077191	1.58E-27	1.30E-25
PDGFB	2.447766168	4.884210528	3.83E-81	3.00E-78
KIF4B	2.438552921	4.03688882	1.08E-13	1.58E-12
LGALS12	2.405690978	-1.740093705	0.002424614	0.005331765
OTOL1	2.398454929	2.61616411	7.33E-05	0.000224663
GABRA3	2.396150087	1.833954039	1.12E-19	3.95E-18
HLA-DRA	2.389669573	-0.307747324	1.39E-07	7.17E-07
PRSS2	2.3561708	3.312328575	4.34E-14	6.73E-13
PCDHB2	2.325883707	2.469934306	2.21E-20	8.54E-19
POTEF	2.321958922	4.631225282	9.15E-19	2.83E-17
DIO2	2.293681555	1.293201797	1.81E-17	4.75E-16
CEBPD	2.264835715	6.188992009	1.96E-41	4.30E-39
CYTH4	2.255027914	0.27426383	2.86E-09	2.02E-08
SLPI	2.241847347	-0.161749738	0.000233499	0.000643304
CD69	2.202221559	0.16791666	3.71E-06	1.46E-05
MB	2.196837127	0.739348443	1.37E-12	1.68E-11
SSX7	2.192600304	-0.825872553	0.000912387	0.002212312
SERPINA5	2.192376461	1.379129655	4.38E-09	2.98E-08
IL6	2.191717699	9.221201668	1.73E-109	1.81E-106
PTX3	2.168497209	7.844145063	6.32E-111	7.91E-108
DMBT1	2.153621235	5.452750986	3.36E-30	3.48E-28
MMP2	2.146683717	2.935059963	6.40E-19	2.02E-17
PDE4B	2.1194923	4.974974604	3.91E-43	9.23E-41
SNORA12	2.118995281	-1.918322155	0.041492913	0.068205065
TNFAIP3	2.1128217	8.462707112	1.26E-38	2.39E-36
FP565260.3	2.111365196	5.683599646	6.63E-52	2.13E-49
CXCL5	2.104721945	0.71888908	1.78E-06	7.44E-06
ICOSLG	2.081332518	5.218206712	1.21E-68	6.29E-66
TMEM204	2.072854977	-0.074758723	3.87E-06	1.52E-05
P2RX6	2.071099094	0.337936264	2.10E-05	7.15E-05
POTEE	2.068548999	6.03628738	1.43E-32	1.75E-30
RAB17	2.058704757	2.727028502	2.33E-14	3.76E-13
PCDHB16	2.050843915	2.076340246	2.61E-14	4.19E-13
C1QTNF1	2.049205895	5.502086584	6.64E-56	2.52E-53
NLRP12	2.029573095	3.326229386	8.48E-22	3.79E-20
SDC4	2.014131832	10.18285531	7.38E-110	8.40E-107
KLHDC9	1.993408427	0.021923585	8.11E-05	0.000246584
CSAG1	1.989752707	4.11272007	5.67E-13	7.39E-12
N4BP3	1.988253799	2.571572164	9.74E-16	1.96E-14
PRKAA2	1.986195054	-0.26443137	0.005262279	0.01079444
H3F3C	1.98461846	5.093260379	2.48E-17	6.33E-16
NKX1-2	1.964525272	-1.519231736	0.004899698	0.010124552
TAF1L	1.963713013	2.977819322	1.64E-18	4.88E-17
KIF25	1.962746378	0.35637087	2.36E-08	1.40E-07
CSAG3	1.955389852	1.991087016	8.72E-10	6.68E-09

PHGDH	1.913121016	4.501225271	3.58E-43	8.61E-41
CSF2	1.911072555	8.989875758	2.22E-64	1.07E-61
NME2	1.908694679	3.423278038	1.08E-07	5.68E-07
MYCT1	1.895360852	0.728054923	2.28E-07	1.12E-06
FMO5	1.889165327	2.916558975	4.60E-11	4.36E-10
NFKBIA	1.888551907	7.847004431	4.29E-93	4.13E-90
WNT10A	1.865072611	2.56288506	6.86E-13	8.72E-12
DDIT4	1.849962049	8.528269435	1.62E-46	4.32E-44
IL1B	1.845459116	9.937662572	6.08E-88	5.08E-85
TNFRSF9	1.844626662	3.570743082	1.31E-30	1.42E-28
HCAR1	1.831652135	1.684824983	2.81E-11	2.75E-10
MAGEA12	1.829037303	5.231344552	1.89E-16	4.28E-15
SEMA3A	1.805533122	1.261774802	4.76E-12	5.35E-11
PTAFR	1.797540387	1.49202363	3.21E-12	3.70E-11
CITED4	1.791275851	1.505280625	1.75E-11	1.77E-10
BBC3	1.790271685	4.5131525	9.77E-38	1.83E-35
VDR	1.786738596	6.433583421	2.28E-77	1.58E-74
IRAK2	1.785220302	6.315307405	4.07E-57	1.64E-54
VGLL2	1.777105395	1.71554181	6.25E-06	2.36E-05
TEF	1.762094408	4.040786413	4.60E-28	4.00E-26
AFF2	1.759103861	-0.060811859	0.000702306	0.001744132
FIRRE	1.743091386	1.581057776	1.11E-07	5.83E-07
PLLP	1.73574093	3.135859285	6.13E-19	1.94E-17
CLEC3B	1.733892253	-1.322150467	0.004157577	0.008731539
EIF5AL1	1.724059701	8.67481959	1.16E-20	4.65E-19
LOXL4	1.715661485	5.599260753	1.59E-15	3.12E-14
HLA-G	1.712489915	2.432376687	3.10E-09	2.18E-08
GLTPD2	1.711455896	0.319651589	1.50E-06	6.34E-06
SCAT1	1.705012491	-0.353169883	0.000170984	0.000485606
TPGS1	1.703739477	3.575062779	1.26E-06	5.41E-06
INAVA	1.687948081	5.996512514	3.41E-28	3.02E-26
NUAK2	1.683212189	7.773685933	1.11E-56	4.33E-54
ARHGEF37	1.676109601	1.922087457	3.02E-13	4.08E-12
SERPINA1	1.670948356	7.711438761	8.35E-81	6.15E-78
MRPS24	1.663601399	2.017503605	5.34E-09	3.55E-08
CHAC1	1.654974028	6.585504605	1.33E-48	3.97E-46
SLC45A1	1.650083212	1.67434239	6.00E-13	7.75E-12
PAEP	1.647255931	2.453280396	6.99E-15	1.23E-13
CLDN1	1.646522276	3.860730712	8.90E-17	2.10E-15
CCDC85B	1.645631986	7.447690053	9.13E-16	1.84E-14
NPB	1.643891666	1.04366566	0.000192042	0.000540506
C11orf86	1.62471638	-0.678452982	0.00126532	0.002966949
GLUD2	1.615684286	6.056375872	1.10E-19	3.92E-18
MIR27A	1.614784298	-1.736819871	0.041681204	0.068478608
ZC3H12A	1.610063747	7.475244097	2.08E-48	6.05E-46
IL2RB	1.6048302	0.48205866	0.000277269	0.000752726
UBE2NL	1.602832183	1.303400875	2.66E-06	1.07E-05
PTGS2	1.600333097	6.134340321	1.53E-24	9.73E-23
PRR29	1.592298814	1.228123818	1.41E-06	5.96E-06
CIITA	1.586439368	1.444734139	4.26E-06	1.66E-05
CACHD1	1.569080081	1.253659674	2.94E-08	1.71E-07
RUNX3	1.568310925	0.628554846	4.91E-06	1.89E-05

USP43	1.561696313	4.455240149	1.23E-32	1.52E-30
HNRNPCL1	1.556002977	1.993803656	2.43E-07	1.19E-06
ELAC1	1.554664046	0.970336188	1.21E-05	4.32E-05
ACOX2	1.554363337	-0.543794282	0.002203669	0.004884514
C10orf55	1.54570417	1.449330931	1.03E-09	7.79E-09
BCAN	1.544764028	1.910044372	8.03E-10	6.18E-09
TMEM238	1.540536321	2.298024997	9.21E-09	5.86E-08
CA9	1.535817055	0.308543252	0.000207647	0.000579606
LTA	1.534193774	1.767266887	7.00E-08	3.80E-07
LGALS2	1.53309312	0.324452526	1.20E-05	4.27E-05
ICAM1	1.529371789	9.823672669	3.34E-72	1.82E-69
KCNK15	1.528867017	0.229833188	5.34E-06	2.04E-05
SLC6A9	1.527684361	6.047928743	7.02E-46	1.79E-43
PDF	1.523988681	1.331959417	2.61E-07	1.27E-06
NUPR1	1.523843843	2.480477012	6.95E-14	1.04E-12
PCDHB9	1.515025324	0.089019018	0.001173892	0.002777013
SEMA3B	1.5133021	3.320793615	2.09E-06	8.59E-06
SMOX	1.512408171	6.276973367	1.44E-40	3.06E-38
VMO1	1.510369855	-0.425752223	0.00161353	0.003692831
NLRP2	1.502862519	4.53821391	3.20E-13	4.29E-12
KIAA0586	-1.502134009	2.888890343	2.12E-11	2.13E-10
TRIM61	-1.502644219	0.755010265	2.43E-07	1.19E-06
CKAP2L	-1.50417875	4.689440801	3.55E-18	1.01E-16
INTS6L	-1.504303052	0.736067709	0.000854302	0.002083578
TAX1BP1	-1.505245126	4.835580304	2.38E-23	1.30E-21
PRMT9	-1.505505518	0.508015176	4.76E-05	0.000151474
ETFRF1	-1.506689172	-0.446648344	5.05E-05	0.000159983
CLDN12	-1.507483157	4.21570905	1.42E-14	2.36E-13
DCTN6	-1.50765589	2.684374255	5.25E-09	3.50E-08
TTLL7	-1.508510969	0.660359961	4.35E-07	2.03E-06
PYROXD1	-1.508962932	-0.40209355	9.02E-05	0.000271787
POLR2B	-1.509295231	5.892215227	5.76E-32	6.81E-30
VCPIP1	-1.511519757	3.556792287	1.48E-11	1.52E-10
TMEM126B	-1.511709655	2.772572461	3.00E-11	2.92E-10
WDR44	-1.512178947	3.738273202	1.77E-17	4.64E-16
KLHL8	-1.512750144	2.965296721	3.33E-15	6.14E-14
EIF2A	-1.512950036	4.048379843	1.21E-14	2.04E-13
POC5	-1.513341656	1.976611661	5.05E-09	3.38E-08
GPR89B	-1.513685195	2.504008789	5.05E-12	5.63E-11
CTDSPL2	-1.514286397	2.932999738	3.41E-10	2.79E-09
PGM2	-1.514562464	4.690455251	1.84E-30	1.94E-28
OSBPL8	-1.514564867	4.020477702	2.66E-06	1.07E-05
BIRC6	-1.515529638	5.227793223	7.75E-12	8.35E-11
ATP5MD	-1.517579932	4.978429322	7.74E-30	7.62E-28
BMT2	-1.518047839	0.344201365	8.68E-07	3.83E-06
SHLD2	-1.518956239	2.829327912	1.18E-09	8.89E-09
CDC40	-1.518991932	2.033798949	3.29E-11	3.18E-10
USP24	-1.519688525	4.981944496	1.44E-13	2.05E-12
SYNE2	-1.520636507	4.025737663	1.98E-16	4.47E-15
GCC2	-1.521318756	2.68411583	1.96E-09	1.42E-08
TYW3	-1.521698542	2.362227396	6.34E-15	1.12E-13
ZNF420	-1.521962632	0.708294768	3.16E-06	1.26E-05

ZNF615	-1.522471749	0.551760571	0.001319339	0.003082012
C7orf57	-1.525580396	-0.201362974	2.86E-05	9.50E-05
MAP4K5	-1.526089588	3.452587311	2.19E-06	9.01E-06
C2orf69	-1.52784956	2.752734913	2.19E-14	3.55E-13
NPIPB6	-1.528449054	0.095535339	0.000274237	0.000745531
RPL18A	-1.529586413	8.625262834	3.29E-09	2.29E-08
DNAJA1	-1.529632413	6.251202147	6.36E-36	1.01E-33
IFIT2	-1.530658516	3.14660995	1.01E-17	2.74E-16
SC5D	-1.530878256	3.711479601	2.50E-11	2.48E-10
PRKAR2B	-1.531098304	0.592887891	0.001535786	0.003534934
NPIPB3	-1.532674978	4.089630948	1.12E-11	1.18E-10
FAM126B	-1.533528737	1.700896564	2.67E-08	1.57E-07
ZNF714	-1.535278612	2.740926717	4.52E-12	5.11E-11
TEX30	-1.535734285	1.700619685	3.23E-09	2.26E-08
BCLAF1	-1.536078394	4.744916955	1.08E-18	3.31E-17
NPIPA2	-1.536846963	1.060224868	2.36E-06	9.63E-06
COMMD8	-1.537223042	1.937936548	1.63E-07	8.28E-07
VSIG2	-1.537391432	-0.756100532	0.000862768	0.002100543
SRFBP1	-1.53743354	2.11835922	6.66E-09	4.37E-08
NAV3	-1.537490904	4.429509335	1.16E-19	4.10E-18
SETX	-1.538117788	4.481139443	3.91E-12	4.46E-11
WRN	-1.538279313	1.849713749	1.93E-09	1.40E-08
LINC02762	-1.538730494	-1.291645767	0.008434139	0.016490236
SACM1L	-1.538807367	2.67333116	3.86E-09	2.67E-08
ZBTB11	-1.539952186	2.764619908	4.48E-10	3.60E-09
HMGB1	-1.541268793	7.048607075	1.47E-23	8.33E-22
DNAJB9	-1.541712923	3.611707376	8.99E-18	2.46E-16
POU2F1	-1.542506063	3.619695041	3.37E-17	8.39E-16
CERT1	-1.542807459	3.06849478	6.23E-15	1.11E-13
NUDT12	-1.542844842	0.681941339	7.07E-05	0.000217183
SLC10A7	-1.543049037	1.069706659	2.05E-08	1.23E-07
ZNF451	-1.543224083	4.103247321	4.76E-10	3.80E-09
GEMIN2	-1.543773118	1.759746584	1.06E-07	5.61E-07
USO1	-1.544014954	5.375577331	8.29E-22	3.74E-20
JKAMP	-1.544454974	3.863352378	2.89E-19	9.49E-18
IL6ST	-1.545304079	5.725425442	5.02E-09	3.37E-08
CLIP1	-1.545925991	4.98683565	5.66E-15	1.01E-13
EML1	-1.546571307	0.545723136	1.85E-07	9.29E-07
STAG1	-1.547985501	2.773281579	4.05E-15	7.40E-14
STRN3	-1.548630755	2.695523263	2.30E-10	1.93E-09
STK17B	-1.548780569	3.073730532	1.13E-11	1.19E-10
FIGNL1	-1.549820469	2.727476268	4.06E-13	5.39E-12
CYTIP	-1.551012431	2.373082041	9.45E-07	4.14E-06
MOB1B	-1.551029147	3.13620832	3.30E-11	3.19E-10
PRPF40A	-1.551187897	4.749572925	4.06E-16	8.74E-15
PUM3	-1.551475132	4.256541747	1.71E-33	2.30E-31
ARHGAP5	-1.552052899	4.023800613	1.56E-05	5.46E-05
VEPH1	-1.55329615	0.718564612	1.40E-08	8.63E-08
SLC35A3	-1.55416006	2.894327822	9.03E-14	1.33E-12
RPL6	-1.555065661	8.368458573	1.40E-15	2.76E-14
UXT	-1.555080772	4.246678282	8.34E-16	1.70E-14
FAR1	-1.556085136	4.020554817	1.87E-09	1.35E-08

MALT1	-1.557001754	3.216030767	1.20E-15	2.39E-14
AC004839.3	-1.558748381	3.561984149	1.70E-11	1.73E-10
BCAP29	-1.558748381	3.561984149	1.70E-11	1.73E-10
SMARCA5	-1.558836883	4.092868069	8.65E-12	9.27E-11
MBOAT2	-1.559006046	4.390261727	2.47E-15	4.65E-14
HECTD1	-1.55937366	5.533053001	2.30E-16	5.11E-15
NUSAP1	-1.559577962	5.326645668	7.10E-19	2.22E-17
BOD1L1	-1.561151878	4.313093119	1.46E-14	2.42E-13
GNB4	-1.562504896	4.630171613	7.01E-17	1.66E-15
NBN	-1.56336365	3.499231549	2.01E-18	5.91E-17
NEK11	-1.563521202	0.515831513	4.11E-08	2.33E-07
SMC2	-1.563546302	3.591907025	6.07E-12	6.68E-11
CCNT2	-1.56597408	2.594803139	1.16E-06	5.01E-06
WDR35	-1.566016419	3.065914122	1.39E-14	2.31E-13
NIPBL	-1.566891521	4.311424735	2.72E-14	4.35E-13
SYDE2	-1.568722381	1.886391513	3.32E-08	1.91E-07
LTF	-1.569500646	-0.735688138	0.002297733	0.005076829
CEP55	-1.570330239	4.506497087	2.24E-13	3.09E-12
USP34	-1.572343886	4.514405643	5.93E-12	6.55E-11
PKN2	-1.572730946	3.385669645	2.50E-10	2.08E-09
RPF2	-1.572947631	3.497725341	1.34E-20	5.27E-19
FSD1L	-1.574095288	0.048890721	3.71E-06	1.46E-05
SOS1	-1.575117051	3.774718981	1.68E-12	2.02E-11
VGLL3	-1.575507981	2.653076217	1.07E-11	1.13E-10
SUV39H2	-1.576524578	2.937819385	3.81E-14	5.98E-13
DDIAS	-1.576646775	2.593441332	1.97E-17	5.13E-16
NPIPB11	-1.577866365	2.322809579	5.22E-08	2.90E-07
RPS13	-1.578132144	7.70458247	1.42E-16	3.25E-15
UBR1	-1.578250112	3.417425955	2.61E-11	2.58E-10
CENPF	-1.580342557	5.877468804	9.12E-17	2.14E-15
ALCAM	-1.580453653	5.323953263	5.93E-20	2.14E-18
TAF13	-1.580678034	3.11053411	2.29E-20	8.82E-19
WWP1	-1.580722367	2.793158917	1.19E-15	2.38E-14
GALNT1	-1.581230732	3.121085796	7.95E-15	1.38E-13
BLID	-1.581251998	-1.516545602	0.014497604	0.026737699
PSD3	-1.581382136	3.639396418	6.40E-22	2.93E-20
POLR3G	-1.581452903	2.236688577	1.19E-08	7.42E-08
XRN1	-1.581504641	1.554020387	7.34E-07	3.29E-06
GPR15	-1.583677811	-1.012848338	0.000806644	0.001978522
PHF3	-1.58376397	4.785263256	1.72E-10	1.46E-09
ZNF720	-1.583851022	0.909730586	5.70E-06	2.17E-05
AHI1	-1.584098871	0.319596748	6.70E-05	0.000206529
ZNF354A	-1.584430335	1.719862841	1.07E-08	6.73E-08
PLS3	-1.584536697	6.291298425	1.97E-39	3.99E-37
INTS8	-1.584771078	3.479659188	2.32E-19	7.74E-18
TBC1D3L	-1.585601923	0.980361005	3.39E-08	1.94E-07
TBC1D3L	-1.585601923	0.980361005	3.39E-08	1.94E-07
CAMK2D	-1.586208026	2.992419909	1.11E-18	3.39E-17
IL6R	-1.589114728	3.656121527	3.01E-13	4.08E-12
IFT80	-1.591300588	0.118730878	0.000118059	0.000347026
DENND4C	-1.591766378	2.412912018	3.48E-07	1.66E-06
IFT74	-1.591936874	0.844496348	1.61E-09	1.18E-08

MFN1	-1.592244082	2.834680327	3.77E-19	1.22E-17
TNPO1	-1.594608407	5.57765078	3.00E-14	4.77E-13
LIG4	-1.594724996	0.207832425	2.76E-05	9.19E-05
EDEM3	-1.594894323	3.307631726	1.17E-08	7.30E-08
FKBP14	-1.595752362	3.431172899	1.45E-11	1.50E-10
MTF2	-1.59618999	2.649761677	2.03E-10	1.71E-09
CCDC186	-1.596305482	1.063314869	5.25E-06	2.01E-05
APAF1	-1.596390679	3.29753272	1.15E-12	1.42E-11
CCDC14	-1.597880761	2.895710969	1.16E-10	1.01E-09
SCLT1	-1.597932014	1.808217263	7.66E-11	6.96E-10
CGB8	-1.601135647	-1.715524247	0.007909406	0.015598276
SMC4	-1.601335113	4.821970568	2.30E-12	2.71E-11
CWC22	-1.601644527	2.224923779	5.98E-13	7.74E-12
UBA3	-1.6028171	2.552877094	2.65E-11	2.61E-10
UBA2	-1.603430235	5.461538775	4.19E-23	2.24E-21
UPF2	-1.604165754	2.702192873	1.34E-11	1.39E-10
PIK3CA	-1.604199131	2.28441843	3.39E-11	3.25E-10
NPIPB5	-1.605292922	4.669267117	2.21E-12	2.61E-11
AKR1C2	-1.605641609	-1.61547173	0.00938137	0.018113104
FAM106A	-1.606081213	-1.192248444	0.001223281	0.002880786
PGM2L1	-1.608956974	2.163620905	2.94E-09	2.07E-08
ZNF322	-1.609826855	2.643811187	1.94E-16	4.37E-15
CD24	-1.610508271	-1.013705116	0.006991619	0.013944253
NEDD1	-1.611752117	2.912537174	1.69E-14	2.77E-13
ABCE1	-1.61241481	5.181823337	1.82E-14	3.00E-13
BIRC2	-1.612640343	5.119129782	4.15E-21	1.76E-19
RAB18	-1.613264553	3.587065956	3.24E-18	9.23E-17
SUCO	-1.615694485	3.905944107	1.21E-09	9.08E-09
PGM3	-1.615971966	4.253847023	7.15E-14	1.07E-12
MRPL13	-1.616317797	4.036581175	6.23E-12	6.83E-11
TRAM1	-1.617248309	5.622742613	1.77E-25	1.26E-23
NGF	-1.617973054	3.215994868	2.87E-09	2.03E-08
ARMT1	-1.618079567	1.963627554	2.35E-08	1.39E-07
RPL34	-1.61960541	6.704030351	7.34E-22	3.35E-20
EXPH5	-1.620431412	3.297775297	4.82E-17	1.17E-15
CKAP2	-1.6213371	5.232035787	4.28E-21	1.79E-19
MGAM	-1.621860187	0.080570419	1.56E-06	6.56E-06
ACAP2	-1.623141318	3.257466857	6.57E-12	7.15E-11
PAQR3	-1.623363953	3.287308308	7.74E-15	1.35E-13
PUS7L	-1.623656732	1.279492526	1.84E-05	6.35E-05
CBX3	-1.624455351	5.098428874	1.41E-17	3.76E-16
GAB1	-1.624688848	1.069936964	4.29E-07	2.01E-06
CAPZA1	-1.6256354	5.858734245	8.11E-25	5.34E-23
NAIP	-1.62716926	0.253213244	0.006507119	0.013092676
PPM1K	-1.627926716	3.041079983	3.16E-14	5.00E-13
CWF19L2	-1.629045431	0.994122029	6.90E-07	3.12E-06
FXR1	-1.629936851	4.6828688	6.02E-16	1.26E-14
CBWD6	-1.630483847	2.335380315	1.20E-10	1.04E-09
SBNO1	-1.630599435	4.723610762	3.21E-14	5.09E-13
CEP85L	-1.63076804	0.451148219	8.73E-07	3.85E-06
WDR75	-1.631870152	3.628497762	2.91E-22	1.40E-20
TMEM263	-1.632037072	4.557738802	9.92E-19	3.06E-17

CCT8	-1.63207773	6.498711138	8.87E-18	2.43E-16
CPE	-1.632094123	-0.373345217	0.000228442	0.000630899
KIF11	-1.632202519	4.090474637	1.39E-19	4.78E-18
CREBRF	-1.632309614	1.603044338	1.72E-06	7.20E-06
EEF1E1	-1.632454369	2.239172713	1.62E-08	9.92E-08
TOP2B	-1.632642745	4.273385817	3.00E-16	6.53E-15
PSME2	-1.634218279	4.26764113	2.30E-23	1.26E-21
PHIP	-1.634453862	3.005048661	4.19E-07	1.96E-06
CBWD5	-1.634477766	2.922519736	3.19E-17	7.99E-16
CLSPN	-1.635035824	4.356116731	4.04E-21	1.72E-19
ZNF30	-1.635388504	-0.105407731	5.55E-05	0.000174189
KRTAP2-4	-1.637161536	-0.630135818	0.000148164	0.000425918
RPS6KB1	-1.637384236	3.176231901	1.20E-09	9.01E-09
ZNF765	-1.637634313	1.705024068	6.52E-13	8.36E-12
HACE1	-1.638562433	1.12103004	0.000497311	0.001276832
NCKAP1	-1.640295736	5.396788606	5.11E-11	4.79E-10
LOX	-1.642664779	4.034288961	2.62E-15	4.88E-14
TCEAL9	-1.643105659	4.00938903	1.32E-14	2.21E-13
COL6A3	-1.643767929	2.590535977	1.09E-17	2.95E-16
FOXN2	-1.647064264	2.295472459	1.35E-09	1.00E-08
STAG3L3	-1.647605376	0.627160536	9.19E-09	5.85E-08
TRIM74	-1.647605376	0.627160536	9.19E-09	5.85E-08
MIS18BP1	-1.649341752	2.653375767	3.24E-07	1.55E-06
ZNF699	-1.650647052	2.864460089	5.90E-09	3.90E-08
PJA2	-1.651239582	4.847667392	4.37E-13	5.77E-12
U2SURP	-1.656783025	4.0834431	8.23E-11	7.42E-10
MYO9A	-1.659639017	3.783032685	2.00E-18	5.89E-17
ADAM9	-1.660134524	6.551118932	1.17E-13	1.70E-12
C2CD5	-1.660920996	1.796361963	8.17E-13	1.03E-11
DEK	-1.66128772	5.862052412	7.53E-25	5.02E-23
FSBP	-1.662878134	1.647279841	5.71E-07	2.62E-06
RAD54B	-1.662878134	1.647279841	5.71E-07	2.62E-06
GDPD3	-1.663367887	-0.685496221	6.08E-05	0.00018933
SEC62	-1.664848213	4.172281461	1.98E-14	3.24E-13
ZNF148	-1.664951255	2.458881319	8.12E-07	3.60E-06
RNF146	-1.66834766	2.383555677	3.34E-15	6.16E-14
PPIAL4G	-1.668705285	-0.770344714	0.000608814	0.001531394
SSX2IP	-1.668852143	2.098134025	2.61E-09	1.85E-08
ZNF780A	-1.669369562	1.225058521	2.77E-11	2.71E-10
NDC80	-1.670782974	3.420963485	6.39E-26	4.76E-24
TBC1D3G	-1.672292603	-0.065597951	3.34E-06	1.32E-05
RAP1B	-1.673855879	4.768965869	4.74E-20	1.75E-18
GTF2H2	-1.674640189	2.332668086	3.08E-18	8.85E-17
GTF2H2	-1.674640189	2.332668086	3.08E-18	8.85E-17
GTF2H2C	-1.674640189	2.332668086	3.08E-18	8.85E-17
VPS13A	-1.675944103	2.865157701	6.28E-10	4.93E-09
DCP2	-1.676316331	4.113388125	7.33E-08	3.97E-07
ZBTB33	-1.678205092	3.077628958	2.03E-13	2.82E-12
SSB	-1.679169602	4.669135978	2.05E-23	1.13E-21
JMJD1C	-1.679731013	3.953228981	5.50E-08	3.04E-07
NFYB	-1.679956683	2.107416321	6.91E-17	1.64E-15
TRMT13	-1.681111138	0.705812887	5.13E-05	0.000162277

EDNRB	-1.681594856	-0.100532193	1.99E-06	8.23E-06
BLZF1	-1.682022418	1.931122878	1.93E-13	2.71E-12
TRMT10A	-1.684775402	-0.615500125	2.59E-05	8.67E-05
RO60	-1.685025746	3.035556744	1.10E-07	5.79E-07
TRAPPC8	-1.685326676	3.293329108	4.42E-08	2.48E-07
SLC35A1	-1.685971163	1.218950071	2.96E-07	1.43E-06
HMMR	-1.686686616	0.380063175	9.40E-06	3.43E-05
RIF1	-1.688441978	4.30228093	4.08E-15	7.45E-14
GSKIP	-1.68940847	2.112423984	1.99E-05	6.82E-05
THOC2	-1.690894432	3.02181201	8.82E-12	9.42E-11
DNTTIP2	-1.691757187	4.383771205	2.23E-12	2.63E-11
KIF14	-1.692753905	3.397720905	3.53E-09	2.45E-08
SLC5A3	-1.693780044	3.823698864	6.98E-10	5.44E-09
SENP7	-1.69971826	0.815419809	1.24E-08	7.72E-08
RPS26	-1.699788714	5.811199174	2.58E-14	4.15E-13
SLCO1B3	-1.700308861	-1.215889025	0.000548653	0.001393506
FABP5	-1.700558856	2.838892914	1.62E-12	1.96E-11
ECT2	-1.700919123	4.175393345	7.27E-20	2.61E-18
THOC1	-1.702516045	2.183259364	1.89E-10	1.60E-09
UBR2	-1.70278623	5.09763316	5.21E-18	1.45E-16
SNX4	-1.703402512	1.472357832	2.91E-13	3.95E-12
KCTD12	-1.703608503	1.869559004	2.46E-13	3.37E-12
SGMS2	-1.705145765	3.630557579	6.04E-12	6.66E-11
LMO7	-1.705894606	3.568601913	3.17E-18	9.05E-17
DENND4A	-1.707157302	3.154159597	2.77E-11	2.71E-10
MORF4L1	-1.70925048	6.229634473	8.09E-23	4.12E-21
PPIG	-1.710391863	2.660698596	3.37E-10	2.76E-09
SLK	-1.71148319	4.10507224	1.89E-11	1.91E-10
USP15	-1.712024747	2.927095862	1.27E-14	2.14E-13
MIER3	-1.712794585	1.807502226	9.39E-11	8.37E-10
GMFB	-1.713919334	5.329443622	1.10E-14	1.88E-13
RABGGTB	-1.714234471	3.977442587	8.06E-21	3.30E-19
SMC3	-1.716001449	4.55828533	2.16E-17	5.58E-16
PSMC1	-1.717238144	5.658417682	1.21E-10	1.05E-09
WDR47	-1.720359395	2.264964208	3.15E-09	2.20E-08
SLC39A10	-1.721880817	3.633140476	1.57E-16	3.58E-15
TRIM23	-1.722427678	1.559965049	0.000257793	0.000704808
KIF5B	-1.722711666	5.0571929	6.75E-13	8.61E-12
DDX60L	-1.722990124	3.021533454	2.06E-19	6.91E-18
ZNF440	-1.723022073	1.592793542	1.54E-14	2.53E-13
HLTF	-1.723723201	2.840982429	9.96E-15	1.71E-13
FRMD4B	-1.728694904	0.035651336	8.23E-07	3.65E-06
PIGA	-1.728998368	2.381062788	4.64E-08	2.60E-07
TPR	-1.729494168	4.709540076	5.48E-12	6.09E-11
CENPJ	-1.729941803	3.042819514	5.61E-20	2.04E-18
ZNF93	-1.730173153	1.381595758	1.96E-07	9.79E-07
ATP6V1D	-1.73052889	4.224690418	1.06E-20	4.24E-19
CCDC146	-1.731679986	-0.364931811	0.000154262	0.000442633
WASHC4	-1.732309965	3.206062845	6.10E-09	4.02E-08
DEPDC1	-1.732400417	3.73828012	2.21E-13	3.05E-12
GLS	-1.735125968	4.674572402	1.33E-22	6.54E-21
ZNF708	-1.735765134	-0.064070958	7.16E-07	3.21E-06

UBL3	-1.736207474	3.3489265	1.66E-09	1.21E-08
SAMD9L	-1.737806018	-1.646604706	0.004510308	0.00938885
KLHL15	-1.738875166	2.989040977	3.08E-17	7.76E-16
GALNT3	-1.739710892	4.014464157	1.30E-20	5.14E-19
SLF2	-1.740417217	3.901800369	1.39E-27	1.16E-25
PLS1	-1.74051467	1.398883521	1.54E-08	9.42E-08
CCDC66	-1.741422262	0.1377194	5.49E-08	3.03E-07
ZNF267	-1.742220136	1.822374916	1.07E-12	1.33E-11
AIDA	-1.742841977	4.854228517	2.83E-17	7.17E-16
DNAJC10	-1.743562367	4.566743703	1.03E-19	3.68E-18
MAD2L1	-1.745886405	4.482104611	5.29E-18	1.47E-16
CEP350	-1.746575439	3.647692282	1.80E-11	1.82E-10
UHRF1BP1L	-1.746921297	2.791236799	1.71E-13	2.42E-12
SHISA2	-1.748158945	2.608397283	4.51E-06	1.75E-05
MIB1	-1.749293967	4.441588926	5.96E-13	7.72E-12
EGR1	-1.749896213	4.691914932	1.64E-15	3.18E-14
SUCLA2	-1.751616324	2.503001109	5.55E-11	5.19E-10
TTC21B	-1.751942646	1.155870334	2.21E-07	1.09E-06
MOSPD2	-1.753501415	2.142651976	0.000506987	0.001298745
ZNF430	-1.753756866	1.185752742	4.66E-13	6.14E-12
ASAH2	-1.753839952	0.650649727	1.52E-09	1.12E-08
NKTR	-1.754144055	2.923120906	4.46E-12	5.05E-11
ZUP1	-1.757333209	1.304931732	6.66E-13	8.53E-12
RND3	-1.758035607	5.259285203	1.52E-11	1.56E-10
GOLGB1	-1.758251515	4.196446528	6.07E-12	6.68E-11
ANKRD12	-1.758996768	2.534331936	9.95E-10	7.55E-09
TWF1	-1.759310019	3.382476551	4.22E-09	2.88E-08
GNL3	-1.76192619	4.59144385	2.93E-26	2.26E-24
CIP2A	-1.763997336	3.036067731	3.75E-08	2.14E-07
SLC40A1	-1.766640301	-0.90507862	8.43E-05	0.000255424
SCAF11	-1.767212548	3.856345596	1.14E-22	5.65E-21
COL1A2	-1.768047003	-1.029531362	0.000312638	0.000838277
RASA2	-1.770005605	2.64022714	6.10E-08	3.35E-07
PDCD10	-1.77294354	2.88909298	1.04E-07	5.52E-07
PPP1R12A	-1.773124578	3.899411231	1.25E-16	2.88E-15
DMXL2	-1.774846311	2.488114037	1.36E-11	1.41E-10
HMGCS1	-1.775469301	4.862842597	4.43E-32	5.28E-30
CHD1	-1.775524248	3.467435865	1.28E-12	1.57E-11
STAG2	-1.77600195	3.500848326	3.45E-09	2.40E-08
ATP13A3	-1.778482424	6.159532762	1.83E-13	2.57E-12
UACA	-1.781748942	4.424808659	6.44E-18	1.78E-16
ATP11C	-1.783008329	2.90266334	7.02E-14	1.05E-12
ALG11	-1.784163312	1.214916055	4.81E-12	5.40E-11
FAM171B	-1.784211725	1.278056048	6.45E-12	7.04E-11
CCDC138	-1.784958298	0.490997819	1.26E-06	5.38E-06
GPR160	-1.785850592	-0.200589827	2.85E-06	1.15E-05
TBK1	-1.78603344	2.042938012	3.26E-17	8.14E-16
ZNF582	-1.786547915	-1.167072946	0.000486372	0.00125157
ZNF638	-1.79030133	3.368495898	2.53E-19	8.32E-18
GPR17	-1.79073251	0.198882319	3.03E-05	0.000100145
ESCO2	-1.792728721	1.494978542	9.12E-11	8.14E-10
N4BP2L2	-1.793893015	2.585068052	4.89E-13	6.43E-12

CDK1	-1.794191971	4.655366411	8.16E-10	6.27E-09
NPM1	-1.797541713	8.214491327	1.19E-20	4.76E-19
ZWILCH	-1.798627662	2.780376707	2.25E-12	2.65E-11
MAN2A1	-1.800040902	4.558802783	3.75E-16	8.13E-15
PGAP1	-1.8000422	0.947318042	2.96E-08	1.72E-07
LRRC40	-1.800102199	1.004395382	6.14E-12	6.75E-11
SNRNP48	-1.80098661	2.610391282	6.02E-20	2.17E-18
CEP152	-1.802016599	1.689948578	9.94E-05	0.000296581
RGPD3	-1.803395672	1.005247028	6.18E-05	0.000192017
ZNF799	-1.804181676	0.425302581	1.20E-07	6.27E-07
SCML1	-1.804236339	3.23438713	3.54E-17	8.76E-16
OPA1	-1.805044653	4.323933288	5.05E-14	7.81E-13
ZBTB41	-1.805345911	1.157204085	7.05E-05	0.00021694
STC1	-1.805723801	5.376029211	6.08E-12	6.68E-11
THEM5	-1.8061642	0.219820666	1.10E-06	4.75E-06
NEK1	-1.807431894	1.536383432	1.04E-07	5.52E-07
CBWD1	-1.808471704	2.757785876	2.06E-21	8.99E-20
FAM135A	-1.80858188	1.845529102	1.56E-07	7.96E-07
RPS27A	-1.810121973	7.960845032	1.02E-23	5.87E-22
PAPOLA	-1.811035542	5.459352673	3.07E-32	3.73E-30
ATP5ME	-1.811152131	3.052396558	2.01E-22	9.78E-21
GXYLT1	-1.814247764	2.937999774	8.15E-13	1.03E-11
ZFYVE16	-1.814492956	2.575002361	3.81E-09	2.63E-08
SNRPE	-1.819695739	4.360966705	5.48E-17	1.32E-15
DNER	-1.820114349	0.306911782	4.98E-09	3.34E-08
AP4E1	-1.82099913	3.127143746	1.28E-19	4.46E-18
BAZ1A	-1.823374162	4.002312458	8.07E-37	1.38E-34
MCEE	-1.82581395	0.913595308	1.01E-10	8.87E-10
SNRPA1	-1.828452467	4.768518324	1.99E-37	3.57E-35
COPS2	-1.828885864	3.623372425	4.81E-16	1.02E-14
ZNF569	-1.831957677	0.87354271	3.73E-06	1.47E-05
CLK4	-1.831957909	0.543176061	2.70E-08	1.58E-07
NAA15	-1.83348247	4.906714046	2.42E-27	1.98E-25
TOP2A	-1.833595885	6.227750891	3.02E-23	1.63E-21
CENPU	-1.838805767	3.021537783	8.77E-16	1.78E-14
NMD3	-1.840185879	2.9081511	1.75E-17	4.60E-16
CDC37L1	-1.841022183	0.573187423	3.93E-10	3.19E-09
SMIM11B	-1.843298683	0.003430321	5.25E-08	2.91E-07
SMIM11B	-1.843298683	0.003430321	5.25E-08	2.91E-07
MORC3	-1.845039791	2.855711267	1.99E-12	2.37E-11
SMCHD1	-1.845323216	3.85261878	1.62E-13	2.30E-12
CADPS2	-1.846641416	0.234753537	2.19E-09	1.58E-08
CYB5R4	-1.849250424	1.941168814	1.98E-11	1.99E-10
DPM1	-1.850178905	3.251098801	1.91E-12	2.29E-11
TCAF2	-1.852388534	1.938459866	5.68E-10	4.49E-09
CBWD3	-1.852583593	2.571864262	3.88E-16	8.39E-15
ROCK1	-1.852712566	4.430919067	6.17E-10	4.85E-09
ARHGAP11A	-1.85274383	5.044704931	1.48E-24	9.45E-23
CEP112	-1.85286367	0.6538644	8.87E-11	7.94E-10
LUC7L3	-1.854229476	3.108684153	1.30E-24	8.41E-23
ORC3	-1.854365089	2.319531168	8.14E-08	4.38E-07
TTLL6	-1.854406373	-1.487952724	0.008702121	0.016934844

CUL2	-1.855835815	3.612850759	2.25E-35	3.48E-33
KIAA1109	-1.857976213	3.665002102	3.23E-09	2.25E-08
EXOC5	-1.861446179	4.791406629	5.13E-16	1.08E-14
DIAPH3	-1.861981909	5.660395438	2.13E-31	2.40E-29
GABPA	-1.862379664	1.978531877	6.62E-14	9.96E-13
FAM91A1	-1.862893536	5.153175261	2.47E-17	6.33E-16
GNG10	-1.862900119	-0.713137408	2.62E-05	8.75E-05
ZNF813	-1.863234389	1.326195852	4.47E-07	2.08E-06
DIS3	-1.867050149	2.04690804	3.49E-07	1.66E-06
TENT5C	-1.867093127	1.065310874	2.81E-11	2.75E-10
RABL3	-1.870087507	2.583448507	3.27E-24	2.01E-22
RPS18	-1.870558726	9.491573496	2.89E-13	3.93E-12
ATP6V1C1	-1.870954831	3.82233792	2.36E-19	7.83E-18
ZNF823	-1.871486973	1.37645167	2.91E-09	2.05E-08
NCF2	-1.872746527	2.303181693	6.34E-22	2.92E-20
RANBP2	-1.874029465	5.081043909	6.20E-13	7.98E-12
KRT17	-1.875310481	0.636883058	5.54E-10	4.39E-09
IDI1	-1.875849911	3.829919533	1.49E-27	1.24E-25
TMTC3	-1.877467482	2.6135206	6.68E-06	2.51E-05
ZNF292	-1.878396904	1.945325773	1.53E-06	6.44E-06
NEMF	-1.878676585	2.456728611	2.31E-11	2.30E-10
KRR1	-1.880283298	2.349201729	5.80E-07	2.66E-06
ARL5A	-1.881559516	3.043789399	2.28E-15	4.33E-14
CHORDC1	-1.882762297	3.825551956	1.35E-23	7.69E-22
RPS24	-1.884909728	7.099950093	6.11E-11	5.67E-10
GRHL1	-1.8865251	-0.965216813	3.11E-05	0.000102556
ABCD3	-1.886718521	3.47043729	4.22E-21	1.79E-19
RPS25	-1.887583479	7.310545904	1.58E-40	3.29E-38
ADAMTS6	-1.889232236	5.62291887	3.00E-33	3.91E-31
SACS	-1.889568705	5.407129446	6.06E-14	9.19E-13
GEN1	-1.890292314	0.683193994	7.29E-09	4.75E-08
ESCO1	-1.891011704	2.333931062	2.24E-11	2.23E-10
PBK	-1.891318153	3.38414659	2.57E-14	4.12E-13
TTC3	-1.894110729	4.661741485	4.67E-20	1.73E-18
CCP110	-1.894731126	1.852633439	8.29E-16	1.69E-14
MIR100HG	-1.896127854	2.615003161	6.33E-15	1.12E-13
KIF15	-1.896253504	1.248762178	1.71E-05	5.94E-05
ANKRD36C	-1.896683153	0.504459658	1.37E-08	8.43E-08
HLA-DOB	-1.897536009	-0.1751431	3.83E-06	1.50E-05
VPS50	-1.898788944	0.784912874	5.49E-07	2.53E-06
SLF1	-1.900920243	0.53636584	8.51E-08	4.57E-07
ZBTB6	-1.901127051	1.571577815	3.52E-14	5.54E-13
MYSM1	-1.904523403	1.794377473	5.62E-14	8.60E-13
CEP57L1	-1.906263472	0.500880717	1.15E-08	7.20E-08
BZW1	-1.908877637	5.997598122	6.02E-28	5.09E-26
USP16	-1.909294835	2.856821606	5.44E-19	1.74E-17
SUGT1	-1.910133726	3.49531038	2.30E-13	3.17E-12
ZNF850	-1.911233994	3.106987703	3.90E-30	4.00E-28
EEA1	-1.91474175	2.629481335	7.93E-12	8.54E-11
RAP2A	-1.92394986	2.04818327	2.82E-19	9.25E-18
TTC37	-1.924253782	3.253554213	3.01E-09	2.12E-08
GOLGA4	-1.925343809	4.023541545	1.71E-15	3.32E-14

GPAM	-1.926194055	2.668805622	2.87E-12	3.32E-11
GUCA1B	-1.928572097	1.018429603	1.64E-13	2.33E-12
CPNE8	-1.928603542	1.557058919	1.50E-08	9.21E-08
SASS6	-1.930381618	1.382646278	2.14E-14	3.46E-13
PRKCI	-1.930678172	3.872666937	1.76E-24	1.10E-22
DYNC1I2	-1.930727665	4.237062079	1.86E-15	3.59E-14
IL1RAPL1	-1.934357912	2.247821339	4.90E-16	1.03E-14
TGDS	-1.936014056	0.100299978	2.02E-07	1.01E-06
SAMD9	-1.936166618	-0.862797314	9.76E-06	3.55E-05
ZNF83	-1.93684234	3.470793968	3.20E-26	2.46E-24
DPY19L4	-1.939496655	2.961031451	6.45E-12	7.04E-11
PNPT1	-1.939931159	3.364309078	8.53E-23	4.33E-21
NME7	-1.940531195	2.998133225	6.41E-24	3.80E-22
USP53	-1.94056741	3.079782373	9.10E-11	8.13E-10
CYP24A1	-1.942027282	3.900069357	4.86E-34	6.84E-32
SMARCAD1	-1.942861309	2.881047703	1.69E-17	4.46E-16
CEP192	-1.945418427	2.949411949	5.10E-17	1.23E-15
AKAP9	-1.94616252	3.214425264	8.41E-12	9.02E-11
NEK7	-1.947131829	4.175803208	2.59E-15	4.84E-14
SMC6	-1.947586396	2.508969057	1.06E-14	1.82E-13
RGPD6	-1.947828579	2.877133355	7.18E-06	2.69E-05
ATAD2	-1.950535742	4.992112426	1.44E-30	1.53E-28
ATR	-1.953056909	2.592008131	4.81E-11	4.55E-10
NUCB2	-1.956522408	3.609922308	8.16E-19	2.54E-17
CEP170	-1.95749268	4.661670827	2.68E-17	6.82E-16
CEP170	-1.95749268	4.661670827	2.68E-17	6.82E-16
METTL14	-1.959721054	1.696400227	3.50E-17	8.67E-16
BLM	-1.960016982	2.792949039	1.51E-23	8.51E-22
TGFBR1	-1.960496163	4.60358114	3.18E-13	4.28E-12
CCDC15	-1.960757137	1.21203738	1.56E-13	2.21E-12
HAUS6	-1.968666772	3.116823315	4.52E-12	5.10E-11
LYSMD3	-1.974183996	2.678630233	1.17E-14	1.98E-13
COBLL1	-1.974816596	1.807306453	2.72E-08	1.59E-07
CHUK	-1.980198992	3.206442808	7.37E-16	1.52E-14
ATM	-1.988172518	3.914602942	1.14E-14	1.94E-13
HSPH1	-1.988463363	6.657504719	3.23E-73	1.93E-70
ERCC6L	-1.989929469	3.158436323	1.68E-21	7.43E-20
SLC38A4	-1.990399688	-1.213668474	0.000317197	0.000849045
INTU	-1.998439552	1.160309045	5.99E-13	7.75E-12
HAT1	-1.999143285	3.777981267	1.11E-10	9.69E-10
AKR1C1	-1.999177798	-0.427395886	9.46E-06	3.44E-05
DLGAP5	-1.999231169	5.330243131	5.02E-58	2.25E-55
PROS1	-2.001495758	-0.409118199	2.03E-07	1.01E-06
SEPTIN7	-2.005304805	4.483668324	6.32E-14	9.54E-13
BRIP1	-2.00553617	2.649122026	4.53E-17	1.10E-15
ZNF114	-2.006218593	2.155647203	7.23E-16	1.50E-14
ZBED8	-2.010202863	0.382961659	2.21E-11	2.21E-10
EIF3E	-2.010537466	6.036765349	7.38E-09	4.80E-08
IFT88	-2.010882614	-1.025101101	0.000118413	0.000347903
NUP107	-2.01173665	3.227060878	1.06E-31	1.22E-29
ODF2L	-2.016052518	2.15122722	1.95E-23	1.08E-21
ANXA1	-2.022295966	5.407904014	3.92E-14	6.13E-13

PPP1R3C	-2.02773432	0.588541945	5.01E-12	5.60E-11
GPR180	-2.027806568	1.034822318	9.79E-14	1.44E-12
NOL11	-2.027878878	3.725597397	5.02E-30	5.03E-28
NUDCD1	-2.028085053	4.941049095	2.19E-15	4.16E-14
TRMT11	-2.029189631	0.961127838	2.21E-13	3.05E-12
PSG8	-2.029660637	-1.160819055	0.000981736	0.00236444
KTN1	-2.036309336	5.83852024	6.10E-15	1.09E-13
ZNF681	-2.041399905	0.074157587	2.79E-08	1.63E-07
PLOD2	-2.042584759	5.173996926	1.17E-14	1.99E-13
AP1AR	-2.043029112	2.003707025	3.32E-13	4.45E-12
AHCTF1	-2.043978434	4.476208547	1.84E-13	2.58E-12
DAW1	-2.044907216	-0.178855202	3.05E-08	1.77E-07
RPS3A	-2.045951155	8.279427358	1.15E-15	2.29E-14
ZNF654	-2.04665166	0.797362705	1.17E-07	6.11E-07
C6orf99	-2.046792558	-1.192691413	9.84E-05	0.000294354
CASP8AP2	-2.048036098	1.461704016	9.53E-11	8.47E-10
COPB1	-2.048591028	6.133554997	8.06E-49	2.46E-46
ZEB1	-2.048939029	2.623995934	2.15E-16	4.78E-15
LAMTOR3	-2.056740776	1.625988957	3.12E-17	7.82E-16
RGPD5	-2.057608771	2.82096233	2.79E-07	1.35E-06
NCAPG	-2.061276472	3.945873465	7.29E-47	1.98E-44
MSS51	-2.062050941	0.194553472	5.10E-10	4.06E-09
G2E3	-2.063721043	2.138186844	3.43E-20	1.30E-18
SMAD9	-2.063807892	0.221215286	1.58E-10	1.35E-09
AC107982.1	-2.066293535	-1.454156371	0.000619923	0.001555586
TMEM269	-2.067191573	-1.979205574	0.003750862	0.007951993
TLL1	-2.067213385	2.472965679	1.50E-18	4.50E-17
CSE1L	-2.069346605	6.567101734	1.18E-53	4.10E-51
CLK1	-2.073210765	2.488534192	9.09E-11	8.13E-10
APC	-2.075287359	2.308349513	1.98E-15	3.79E-14
FANCM	-2.076315696	0.670300367	1.36E-09	1.01E-08
NPAT	-2.076619928	2.989840347	2.76E-25	1.93E-23
RASGRP3	-2.077671933	-0.817802754	0.000232073	0.000639515
UBA6	-2.078870117	3.95260244	7.50E-14	1.12E-12
RANBP6	-2.080517212	1.851517728	5.65E-12	6.26E-11
ZGRF1	-2.086156676	0.176411757	2.02E-09	1.46E-08
HSP90B1	-2.087826984	8.359136047	3.18E-25	2.20E-23
BRCA2	-2.087941834	3.491895103	6.84E-16	1.43E-14
HSP90AA1	-2.089272834	8.44450738	1.34E-23	7.66E-22
KIF20B	-2.089377533	3.109102695	8.55E-14	1.27E-12
CAVIN2	-2.09032302	4.263357135	1.78E-23	9.96E-22
ESF1	-2.091550967	2.071221536	8.33E-22	3.74E-20
DDX60	-2.094130034	1.041996619	5.11E-12	5.69E-11
DST	-2.095248799	6.449711828	1.74E-24	1.09E-22
TBC1D15	-2.096555246	1.798903242	4.06E-20	1.51E-18
EDN2	-2.096651523	0.896431644	6.82E-15	1.20E-13
CEP97	-2.097921575	2.166051467	2.00E-17	5.19E-16
NAT1	-2.098393302	0.442851015	5.21E-12	5.79E-11
ZFC3H1	-2.098410616	3.499224408	8.13E-16	1.67E-14
CAMK1G	-2.101977793	-2.302130619	0.018885711	0.033833747
LGALS9B	-2.103906046	-2.303024806	0.02043205	0.036343958
PIK3R1	-2.105056218	1.295591717	4.22E-16	9.02E-15

KPNA5	-2.106016011	-1.238561827	7.40E-05	0.000226459
ZNF382	-2.111524551	-0.986694393	2.14E-05	7.27E-05
FANCB	-2.114026447	0.580757145	1.03E-11	1.09E-10
OLAH	-2.121579536	-1.426151733	0.000103626	0.000308438
CU634019.1	-2.125463702	3.067591101	1.20E-11	1.26E-10
RICTOR	-2.125845522	3.230513052	6.10E-13	7.87E-12
TMF1	-2.130163401	2.867364952	4.77E-09	3.22E-08
TUBA4B	-2.132148094	-0.563695798	4.32E-06	1.68E-05
TMEM161B	-2.137895828	1.738191433	4.79E-13	6.31E-12
ANKRD30B	-2.142823427	-0.345980067	2.48E-06	1.01E-05
POLQ	-2.146406915	1.433256021	2.80E-17	7.11E-16
KNTC1	-2.147219571	2.785659927	3.31E-29	3.16E-27
AGTPBP1	-2.151532797	3.003699018	2.06E-19	6.91E-18
RAD51AP1	-2.152900496	1.359706732	1.95E-12	2.32E-11
ZNF165	-2.154198968	2.949401587	1.35E-19	4.68E-18
MTREX	-2.155559345	2.486268132	7.89E-11	7.14E-10
LGR4	-2.160926618	2.613995105	9.24E-15	1.60E-13
RB1CC1	-2.162256394	3.418307264	1.28E-14	2.15E-13
HBS1L	-2.164797945	3.175349801	6.00E-39	1.17E-36
PPIP5K2	-2.16730218	2.60729897	3.96E-07	1.87E-06
SLC4A7	-2.170353241	3.04800557	7.22E-13	9.16E-12
ELMOD2	-2.175041381	1.494154859	5.81E-20	2.11E-18
TVP23B	-2.175665451	2.981363816	4.82E-20	1.77E-18
LIFR	-2.186277402	3.035794236	5.95E-13	7.72E-12
KITLG	-2.190244022	-0.492183816	2.98E-07	1.44E-06
MALAT1	-2.192951354	8.412762136	3.76E-11	3.59E-10
EMC2	-2.193824137	2.061894488	3.10E-17	7.80E-16
CDC26	-2.199744768	3.05580273	8.47E-19	2.63E-17
CPEB2	-2.209231503	2.16669617	1.87E-09	1.35E-08
LIN9	-2.20987107	1.470418144	1.19E-18	3.59E-17
ANKRD26	-2.211756034	0.338075331	2.68E-10	2.22E-09
COG6	-2.212595663	2.133599259	8.10E-25	5.34E-23
SERPINA9	-2.21453111	-2.467414213	0.018654399	0.033472041
CCDC82	-2.215332771	2.788359372	2.66E-24	1.65E-22
RGPD2	-2.215817266	-0.251041447	8.70E-09	5.57E-08
RPAP3	-2.218618195	1.9836536	4.51E-16	9.60E-15
TBC1D3K	-2.218916138	-0.119264534	1.33E-08	8.23E-08
TBC1D3K	-2.218916138	-0.119264534	1.33E-08	8.23E-08
RASSF8	-2.220516435	1.786800072	8.38E-16	1.70E-14
ZNF804A	-2.22374466	0.210451491	9.89E-10	7.52E-09
SWT1	-2.231743737	-0.746790552	3.37E-05	0.000110422
LGALS9C	-2.236191756	-1.706787961	0.003477682	0.007439696
CEBPZ	-2.237462087	3.621159854	3.03E-36	4.92E-34
WDHD1	-2.240742205	3.970816	3.39E-37	5.98E-35
TRPC1	-2.247070433	-0.045869194	4.62E-10	3.70E-09
LARP7	-2.252976146	1.555591866	6.89E-19	2.16E-17
FAM133B	-2.257542403	0.289976193	1.10E-12	1.36E-11
CHM	-2.258008921	2.216176676	1.20E-22	5.93E-21
KNL1	-2.261907264	3.313616082	1.40E-13	2.01E-12
ARHGAP29	-2.267637079	5.687443593	1.60E-53	5.43E-51
PNPLA8	-2.273482721	2.667318286	3.62E-20	1.37E-18
FP236241.1	-2.274401898	3.097758386	1.30E-26	1.03E-24

CU633904.1	-2.274467653	2.999186192	2.75E-40	5.63E-38
PLK4	-2.278582789	2.385227555	4.41E-32	5.28E-30
KRT13	-2.278933674	-1.456567064	5.40E-05	0.000169877
CCDC144A	-2.286618867	-0.687667984	1.87E-06	7.77E-06
IBTK	-2.291412589	3.824329987	7.48E-29	6.93E-27
CRYBG3	-2.296333398	2.456238431	1.27E-20	5.04E-19
ABHD12B	-2.297936008	-1.856310346	0.000819694	0.002006991
LTN1	-2.302204917	2.893413767	6.03E-11	5.59E-10
YOD1	-2.303249425	2.565426397	4.12E-11	3.91E-10
RPL39	-2.304477732	7.657759789	1.13E-38	2.18E-36
OPN1MW	-2.305042097	-1.211867591	1.19E-05	4.24E-05
GPR155	-2.305269234	0.566509376	9.64E-11	8.56E-10
ARHGAP42	-2.305745962	2.782456874	4.33E-14	6.73E-13
ERO1B	-2.317882453	1.555062184	5.89E-12	6.50E-11
ODR4	-2.318716417	1.322480747	1.78E-20	6.95E-19
ZNF563	-2.32280782	0.109774989	1.08E-10	9.46E-10
BDH2	-2.324579678	-0.585260788	7.00E-07	3.15E-06
NUF2	-2.325751249	2.957463365	2.07E-18	6.08E-17
RPS7	-2.333805065	7.352565737	1.70E-25	1.22E-23
MIR7-1	-2.346252343	-2.408686354	0.012001045	0.022589247
AIM2	-2.347933887	-1.6761674	0.000171415	0.000486609
FGF5	-2.355379941	1.249011579	4.53E-10	3.64E-09
TSPAN12	-2.358894639	0.169023435	2.39E-12	2.79E-11
NFE2	-2.367385244	-1.819333894	0.000387416	0.001015182
PIBF1	-2.374409029	-0.134287701	1.27E-09	9.51E-09
MAPK15	-2.378292078	-1.070803735	2.51E-06	1.02E-05
FAM111B	-2.379416636	1.22386983	5.31E-15	9.52E-14
GAS5	-2.384211627	4.908138056	3.60E-23	1.93E-21
MAMDC2	-2.385391341	4.970417919	2.89E-76	1.91E-73
PSMA3	-2.38674617	3.611176352	2.21E-42	5.04E-40
EIF4E	-2.391420138	3.272126603	1.24E-43	3.03E-41
MIR142	-2.396043443	-2.653570786	0.029693871	0.050542159
RWDD3	-2.396986123	-0.361166617	2.66E-09	1.88E-08
TBC1D23	-2.412532982	3.007248647	2.33E-22	1.13E-20
GRAMD1C	-2.41270272	-0.856935026	8.97E-07	3.95E-06
ZNF117	-2.413534283	-1.778085144	0.001225687	0.002885367
RECQL	-2.420674207	2.610235	4.26E-16	9.11E-15
ASPM	-2.426542223	3.79645823	3.23E-24	1.99E-22
CU633967.1	-2.443403998	3.170731929	2.25E-25	1.58E-23
ID2	-2.448477566	-0.839551955	5.83E-08	3.21E-07
CHD9	-2.450741815	2.55476935	4.13E-29	3.89E-27
TIE1	-2.456311683	-1.608483282	8.05E-05	0.000244821
COL15A1	-2.459767758	0.843784392	3.31E-18	9.42E-17
PSG5	-2.460960889	1.264005336	1.81E-21	7.94E-20
EPGN	-2.461846829	-1.609360735	8.16E-05	0.000247869
ANLN	-2.484724477	6.176856318	1.36E-52	4.48E-50
RPS21	-2.485130079	6.39298031	5.00E-29	4.67E-27
MTERF1	-2.486782198	0.746324015	4.76E-11	4.51E-10
GCNA	-2.48795465	0.050289011	2.69E-12	3.12E-11
TBC1D8B	-2.493453725	0.182091149	1.91E-12	2.28E-11
BCL2A1	-2.494979316	-2.104585022	0.00228356	0.005047296
ZRANB2	-2.496792711	2.24442622	2.74E-11	2.69E-10

HSPA4L	-2.50279394	2.523331375	3.40E-34	4.90E-32
ADGRL2	-2.504527592	-2.105054193	0.002087518	0.004655082
PSMC6	-2.505114926	3.855287576	4.87E-18	1.37E-16
KRTAP2-3	-2.506416634	2.531868693	6.87E-33	8.69E-31
RGPD8	-2.518362199	2.579973579	1.79E-16	4.07E-15
ZNF92	-2.522480234	-0.260569131	5.98E-10	4.72E-09
RFPL4AL1	-2.53757591	-1.894590146	0.000164147	0.000468312
NAE1	-2.542997124	3.284233456	7.74E-34	1.08E-31
MIR222	-2.560379957	-2.58821489	0.009149538	0.017690049
RPS28	-2.566895704	5.859195597	1.22E-14	2.06E-13
DDIT4L	-2.599903608	-2.061591564	0.001278578	0.002994112
GOLGA6L4	-2.604637348	-0.579485711	0.000104363	0.000310337
CEP170	-2.617046263	0.425590262	4.05E-13	5.38E-12
CEP170	-2.617046263	0.425590262	4.05E-13	5.38E-12
TTK	-2.625899764	2.642858511	1.91E-14	3.13E-13
DOCK11	-2.650891325	0.321019217	1.01E-14	1.73E-13
IFI44	-2.665591852	-1.483064438	9.87E-06	3.58E-05
SELENBP1	-2.670875284	-0.428519392	7.76E-11	7.04E-10
COL17A1	-2.681510968	3.823753618	3.70E-57	1.54E-54
FOS	-2.682917551	1.612984285	4.91E-28	4.24E-26
CENPK	-2.691414393	1.608033779	6.74E-16	1.41E-14
GCNT4	-2.715470711	-1.452592254	1.90E-06	7.88E-06
PSG6	-2.724776428	-0.937409372	2.08E-08	1.25E-07
SGO2	-2.729981954	2.178085785	2.38E-28	2.13E-26
CLGN	-2.734327853	1.100607063	9.02E-23	4.55E-21
LEPR	-2.763320524	0.512757579	1.43E-11	1.47E-10
MOB3B	-2.764767216	1.394417393	5.72E-27	4.65E-25
RGPD4	-2.768355164	0.715757993	9.12E-17	2.14E-15
FRMPD3	-2.829156504	-0.979154149	2.32E-07	1.14E-06
PRELP	-2.834915836	-2.464676297	0.011264217	0.021349997
CRIP1	-2.846628225	-2.466236167	0.003082454	0.00665791
CENPE	-2.855794542	2.660944135	1.08E-17	2.91E-16
LYPD5	-2.884328027	-1.890698362	0.000148658	0.000427041
OPN1MW3	-2.899413815	-1.041719727	2.02E-08	1.22E-07
OPN1LW	-2.903092365	-1.671151544	2.85E-05	9.46E-05
RPL21	-2.929169057	6.480830458	7.72E-22	3.50E-20
POLK	-2.929216148	1.296947754	2.33E-23	1.27E-21
SYCP2L	-2.931415456	-1.158794487	3.33E-08	1.92E-07
RPS27	-2.990405675	6.511463171	1.37E-19	4.73E-18
SARNP	-3.046752361	1.651380068	4.49E-26	3.38E-24
RPL7	-3.139961719	6.753542081	1.84E-15	3.54E-14
PTBP2	-3.170334876	-0.394858514	6.80E-13	8.67E-12
RPL9	-3.219577989	7.022348593	2.84E-47	7.90E-45
GOLGA6L3	-3.250627266	-1.25615598	9.01E-08	4.80E-07
RGS2	-3.251839588	-0.529721773	3.51E-13	4.69E-12
MMP3	-3.268977293	4.39749948	3.01E-25	2.10E-23
CCNE2	-3.272068904	1.167535487	1.24E-11	1.29E-10
OPN1MW2	-3.292165384	-0.888900194	2.57E-10	2.14E-09
MMP1	-3.293456344	6.71005792	1.75E-72	9.94E-70
DNAJB4	-3.308152929	1.113116455	4.68E-12	5.26E-11
MYOZ2	-3.310255909	-2.650851368	0.009071743	0.01755862
ACKR3	-3.311484639	-2.650699741	0.00909472	0.01759765

PSG1	-3.320570052	-1.891195214	3.04E-05	0.000100647
HSPA1B	-3.323258091	9.018934867	1.26E-149	3.15E-146
CPNE7	-3.327685089	-1.390374725	5.42E-08	3.00E-07
MSLN	-3.345272369	-1.602926641	3.24E-06	1.29E-05
MT1G	-3.4546157	-2.586141595	0.005564662	0.011364438
TATDN1	-3.459647883	-0.146949374	1.96E-17	5.11E-16
SCFD1	-3.487616177	2.098123855	1.42E-42	3.29E-40
HSPA1A	-3.489534612	8.68834055	6.05E-148	1.26E-144
MTRNR2L12	-3.553261014	0.240519646	1.47E-11	1.51E-10
CNN1	-3.569135156	-0.406990211	3.89E-14	6.09E-13
RHOJ	-3.589082736	-2.523830952	0.003202905	0.006902595
FOLR3	-3.611881612	-2.058371731	2.20E-05	7.46E-05
KBTBD8	-3.670719773	-0.748888603	7.49E-11	6.81E-10
ARC	-3.687150665	2.06646395	4.32E-28	3.78E-26
RFPL4A	-3.723936511	-1.635630965	2.79E-07	1.35E-06
MCTP1	-3.726033577	0.673286914	2.04E-26	1.60E-24
MTRNR2L8	-3.849979994	0.493860178	1.61E-15	3.13E-14
IGIP	-3.990721494	-0.492453686	1.93E-16	4.36E-15
FBXL7	-4.02867134	-2.297822784	0.000211646	0.000589191
GCKR	-4.058161498	-1.775177536	1.78E-07	8.95E-07
HSPA6	-4.116265258	4.27697262	5.07E-127	7.93E-124
MIR221	-4.136331582	-1.035539431	1.24E-12	1.52E-11
ALOX15B	-4.174648802	-3.026266392	0.036208372	0.060366575
GJA5	-4.176534385	-3.02607378	0.035887464	0.059903355
GK	-4.31935354	0.599696346	1.36E-30	1.45E-28
KRTAP19-5	-4.424852498	-2.94286541	0.018762698	0.033637436
PAGE2B	-4.425885319	-2.942753694	0.018777222	0.033658654
CRCT1	-4.426971901	-2.942682363	0.019518388	0.034867395
ADCY10P1	-4.43660044	-2.05670426	1.20E-06	5.16E-06
ZNF404	-4.507143346	-2.012648211	3.71E-07	1.76E-06
MTRNR2L6	-4.573401976	-1.969954564	2.51E-07	1.22E-06
MMP10	-4.590900933	-0.989739014	5.84E-14	8.90E-13
SAA2-SAA4	-4.635053432	-1.928773438	1.35E-07	6.98E-07
MIR125B1	-4.641078615	-2.863713714	0.010888926	0.02072024
MTRNR2L10	-4.861426155	-1.773651131	2.16E-08	1.29E-07
S100G	-4.917591454	-1.73682475	4.07E-09	2.79E-08
SPANXN3	-4.98689896	-2.717888512	0.001480239	0.003417132
KCTD4	-5.267465762	-2.584998729	0.000430909	0.001119201
PPIAL4F	-5.608858109	-2.405364497	8.29E-05	0.000251739
DEFB103B	-6.287771653	-0.635650646	1.81E-19	6.16E-18
DEFB103A	-6.419600198	-0.518091365	5.67E-21	2.34E-19

Supplemental Table 3. Genes significantly regulated following 5 days of 1nM E2 treatment + the addition of 20ng/mL TNFα for the final 3 hours in ERβ expressing MDA-MB-231 cells.

Gene	logFC	logCPM	PValue	FDR
F13A1	9.46032249	3.93027114	6.28E-80	2.02E-77
MAB21L4	8.761734096	4.131705954	4.90E-88	2.36E-85
CXCL14	8.729687593	6.451599105	1.22E-111	7.65E-109
TNS4	8.368343067	7.146442622	6.17E-314	7.72E-310
CST5	8.262791224	2.76261212	9.97E-54	1.66E-51

MGAT3	8.169807748	3.551037979	5.98E-75	1.59E-72
S100A7	8.044748706	2.550222071	5.78E-32	4.39E-30
KRT13	7.865868995	5.988158135	1.91E-132	1.71E-129
CST2	7.739438874	4.497455746	3.60E-62	8.19E-60
LOXL4	7.466508858	10.96329354	1.29E-236	4.05E-233
TFF1	7.403841353	-0.689960277	2.75E-11	4.28E-10
FGFBP1	7.380758209	1.923023801	4.60E-37	4.68E-35
CYTH4	7.209565322	4.742850822	3.56E-140	4.45E-137
ALPP	7.11556154	8.204670991	6.29E-252	2.62E-248
CST1	7.008945239	8.257414608	1.36E-59	2.71E-57
CCN5	6.966057726	2.679627495	5.27E-60	1.08E-57
ALPG	6.909121638	4.588434695	8.05E-110	4.80E-107
GJA5	6.837805856	2.556687346	1.83E-57	3.52E-55
TMPRSS3	6.676893252	2.398162227	5.71E-45	7.45E-43
SYT8	6.667882099	-1.274799912	1.01E-08	1.05E-07
S100A9	6.640517617	2.368690671	4.59E-46	6.12E-44
TMOD1	6.560450877	3.948817687	6.44E-57	1.19E-54
SERPINA9	6.369384814	3.02918746	9.87E-36	9.43E-34
CPZ	6.366823281	1.829409953	6.41E-32	4.83E-30
B3GNT6	6.35680742	1.453432165	8.12E-37	8.13E-35
MIR622	6.326855074	5.069303084	3.00E-15	7.27E-14
GPR78	6.287788963	-1.58112412	9.17E-07	6.42E-06
CST4	6.204661633	7.183905817	1.19E-50	1.84E-48
RNF223	6.200097938	5.149416891	4.23E-61	8.97E-59
GCNT3	6.115848466	2.101756065	8.75E-34	7.36E-32
TUBA3E	5.974628308	1.452977109	3.06E-35	2.78E-33
SEMA3B	5.965223481	7.323318327	4.46E-67	1.12E-64
KLRC4	5.959978714	-1.876429122	0.000614787	0.0020148
DPYSL3	5.957291305	-1.79631487	4.74E-06	2.76E-05
ELF3	5.91056356	7.579481541	2.57E-310	1.61E-306
ABCB1	5.885445291	-0.203681623	1.23E-14	2.80E-13
OLFML3	5.721825927	3.293446003	2.31E-54	3.90E-52
AARSD1	5.717934231	-0.277903965	0.001481181	0.0043033
CALHM5	5.604379351	1.112816002	3.38E-24	1.63E-22
HAVCR2	5.570455458	4.447321609	3.92E-47	5.33E-45
PADI4	5.560701339	4.147257841	1.37E-60	2.85E-58
SERPINA6	5.43999892	0.615360432	5.59E-25	2.90E-23
VASN	5.386506086	7.064440951	2.41E-119	1.77E-116
PLEKHS1	5.286037876	2.723233318	2.67E-41	3.07E-39
SOD3	5.165560338	1.433424532	2.57E-24	1.26E-22
KRTAP9-1	5.104821419	-2.322089786	0.000967988	0.002976
INHBB	5.066632524	4.47732594	5.81E-85	2.35E-82
IL1R2	5.058071082	1.653166558	1.16E-31	8.59E-30
ACKR3	4.967679909	1.571597202	4.56E-31	3.30E-29
NAT8	4.966431842	-0.264404593	1.08E-14	2.48E-13
CADM1	4.953591187	-2.413789341	0.001027384	0.0031347
LYZ	4.859519573	0.424754994	1.06E-13	2.16E-12
LUM	4.836170584	-2.477300595	0.013881697	0.0293061
RAB37	4.698360199	4.95853593	3.12E-93	1.63E-90
LAMC2	4.643503021	8.87807886	3.30E-154	6.90E-151
SLC2A5	4.596286051	0.910342742	2.20E-22	9.57E-21
NHSL2	4.590203418	6.250324598	3.35E-19	1.15E-17

DEFB1	4.574690593	-0.590687091	5.58E-08	5.12E-07
PDGFB	4.49046976	6.725672284	3.22E-84	1.18E-81
SPATA31D4	4.486246226	2.325593718	1.05E-27	6.69E-26
CDH1	4.426475003	4.409513248	2.14E-38	2.23E-36
NXPH3	4.371851664	2.942996031	1.49E-44	1.90E-42
DNAJC12	4.356591935	-1.495809253	0.000344515	0.0012197
PRPS1L1	4.355248098	2.55127174	1.50E-16	4.04E-15
GREB1	4.321030474	4.754001318	1.44E-80	4.88E-78
KLRC2	4.295577673	2.819560436	2.69E-09	3.10E-08
AQP3	4.285304147	5.469178072	5.33E-125	4.17E-122
SYBU	4.277853265	3.278868607	8.54E-49	1.24E-46
KCNK15	4.236277047	2.435948131	2.13E-35	1.99E-33
SPATA31D3	4.234359825	2.154305843	1.76E-21	7.10E-20
IFNL1	4.210484634	0.742183184	4.54E-22	1.93E-20
C1orf116	4.183609591	4.398484128	2.84E-36	2.78E-34
NPFFR2	4.129665544	0.311512708	3.09E-12	5.40E-11
SPATA46	4.124620937	0.085509772	7.07E-13	1.32E-11
RASGRP1	4.115305471	3.25812989	3.56E-43	4.33E-41
RETREG1	4.070755844	1.576960318	2.16E-09	2.52E-08
PTTG2	4.054910774	2.31664791	6.27E-09	6.74E-08
CLEC4M	4.042555568	2.298369015	5.39E-22	2.26E-20
NAP1L3	4.040010244	0.396431207	4.95E-11	7.48E-10
ENTPD8	4.038982056	1.727361299	1.57E-26	9.19E-25
GASK1B	4.015549745	0.831527349	1.51E-11	2.44E-10
BDKRB1	3.967547254	0.531188213	3.91E-16	1.02E-14
EDARADD	3.966835541	9.997326265	6.44E-17	1.80E-15
CDC42BPG	3.905340176	4.1553192	4.36E-44	5.46E-42
DAPP1	3.896656595	-1.132503514	1.30E-05	6.79E-05
PDZK1	3.888808154	3.302317253	4.59E-26	2.60E-24
IRX3	3.868384294	7.848396969	1.07E-145	1.49E-142
GJA1	3.863843268	3.880054601	1.06E-14	2.45E-13
SERPINA3	3.857873914	2.711670875	3.84E-10	5.06E-09
FAM71D	3.83621965	3.350708702	2.87E-06	1.77E-05
TFF2	3.797465065	0.786622084	1.73E-20	6.41E-19
PRSS23	3.789344421	10.27004069	7.84E-140	8.92E-137
AFF3	3.778652373	2.396130331	9.89E-27	5.92E-25
ISM1	3.771292153	1.173136252	1.00E-25	5.44E-24
KLRC3	3.765122551	1.664334636	3.42E-14	7.44E-13
PRKACG	3.763578624	1.561259147	6.32E-09	6.78E-08
ITGB8	3.756515127	2.766628046	5.54E-22	2.31E-20
EFNA1	3.742389868	7.91346644	1.74E-61	3.82E-59
FRMPD3	3.703507986	2.658311057	2.44E-22	1.05E-20
FAM25A	3.692723526	-0.273561073	2.48E-11	3.85E-10
FAXDC2	3.678697028	4.476877141	5.37E-49	7.91E-47
PCDHB6	3.669162304	1.326405428	1.90E-21	7.62E-20
HECW1	3.6333913	3.095674308	4.03E-32	3.10E-30
SFTPD	3.609680673	0.84763393	4.19E-18	1.32E-16
FRAS1	3.600007597	4.635024715	1.89E-25	1.01E-23
PLET1	3.574879972	-0.375373263	1.77E-11	2.84E-10
CALB2	3.569259008	7.363276866	1.69E-151	2.65E-148
DAPK2	3.56022443	4.265947046	5.81E-57	1.10E-54
PLPP3	3.554000936	4.913833641	7.16E-62	1.60E-59

CRISPLD2	3.533589358	6.657926774	6.83E-115	4.50E-112
PCDHB5	3.532962721	0.574561992	7.78E-09	8.26E-08
PLA2G4D	3.529507773	2.673066891	1.70E-33	1.41E-31
ZNF534	3.520922262	2.643887593	1.84E-06	1.20E-05
NACA2	3.505993845	5.245863309	6.45E-14	1.36E-12
LTB	3.482865161	6.203658808	1.76E-17	5.20E-16
MIR503	3.476344491	-1.433494605	1.09E-05	5.81E-05
H19	3.463228786	-0.0236464	3.49E-07	2.69E-06
RUBCNL	3.453450166	1.338996594	3.14E-22	1.35E-20
DOK7	3.450785271	1.138048839	6.91E-18	2.14E-16
LSMEM2	3.444583741	1.394819353	4.73E-20	1.73E-18
UBD	3.438422026	-1.045330081	4.49E-07	3.37E-06
MAGEA10	3.385090677	1.399959368	1.23E-07	1.05E-06
TNF	3.358806613	2.294625909	2.76E-27	1.72E-25
TNFSF14	3.341334066	0.611167448	1.03E-15	2.59E-14
OVCA2	3.318549891	2.626917531	8.75E-09	9.22E-08
CDH3	3.305480012	5.61170761	6.35E-57	1.19E-54
GPER1	3.305461185	2.181012924	4.62E-19	1.56E-17
CEMIP	3.300854438	7.883336918	3.37E-134	3.25E-131
ERVV-2	3.289920341	0.567996289	6.80E-15	1.59E-13
FAM222A	3.283822044	3.540507203	1.18E-31	8.66E-30
IGFBP5	3.279549229	-1.190132694	1.68E-05	8.55E-05
PANDAR	3.276021776	3.903542277	6.51E-09	6.97E-08
SPATA45	3.268575167	-2.147730921	0.001993235	0.0055899
CSNK1A1L	3.260241372	1.826490668	5.21E-11	7.85E-10
SYT1	3.256893741	0.402460327	1.89E-06	1.23E-05
KHDC1L	3.251765206	1.290645722	2.84E-18	9.12E-17
SEMA3A	3.201985053	2.354161859	2.38E-17	6.91E-16
CD34	3.149282882	4.986155218	4.90E-63	1.16E-60
ITGB2	3.143537777	3.218607684	6.62E-29	4.46E-27
NCAM2	3.134105832	1.485948556	1.18E-09	1.44E-08
NPB	3.115114154	2.180516349	4.30E-08	4.04E-07
BEST1	3.108422022	1.650614052	1.14E-18	3.74E-17
PCDHB8	3.107414855	0.782588421	2.14E-10	2.92E-09
CD69	3.098155608	0.797232414	0.000789137	0.0025017
ABCG2	3.088579704	1.391060901	1.73E-08	1.73E-07
ANP32C	3.080872461	3.098392467	4.86E-08	4.50E-07
CD83	3.06825662	5.893716083	2.61E-66	6.40E-64
OVOL2	3.065525995	0.653077389	9.65E-10	1.19E-08
SLPI	3.054230322	0.466403248	7.72E-12	1.30E-10
IGFBP4	3.03411991	12.24332192	1.31E-130	1.10E-127
C11orf98	3.03348412	2.085609401	0.000626132	0.0020488
B4GALNT2	3.022965498	-0.657871474	0.000453324	0.0015476
CD24	3.010836708	1.718191421	2.32E-15	5.63E-14
TBXA2R	3.009812363	3.990995412	3.99E-36	3.87E-34
PTAFR	2.997472762	2.422323436	2.71E-27	1.69E-25
FRK	2.994399225	-1.093324759	1.05E-05	5.62E-05
TNFAIP2	2.989747544	9.922340931	1.44E-83	5.16E-81
WNT7B	2.984531855	3.531665105	5.29E-27	3.23E-25
TGFA	2.976790888	8.513905448	2.68E-26	1.54E-24
FGD3	2.955889663	0.715581692	2.24E-16	5.94E-15
CYP26B1	2.954947142	6.648069224	8.00E-51	1.27E-48

GSN	2.949359897	8.494580773	1.59E-84	6.04E-82
ZNF219	2.941929341	4.062431378	1.19E-23	5.52E-22
RGS9	2.940505875	3.298050085	3.25E-33	2.66E-31
CCL2	2.903752637	-0.526693192	5.55E-05	0.0002468
GSPT2	2.903248639	1.971703025	4.77E-06	2.77E-05
SERPINB6	2.889234048	9.530479185	2.63E-119	1.83E-116
CD300C	2.888304184	2.084875863	7.42E-24	3.48E-22
DRGX	2.886513705	2.206775543	1.18E-14	2.70E-13
SERPINA1	2.883773864	8.711331347	1.63E-134	1.70E-131
C18orf32	2.876962443	0.253669614	0.000139276	0.0005548
ADAM12	2.868759614	2.512897614	4.81E-14	1.04E-12
TCN1	2.867442219	2.935095313	2.18E-14	4.88E-13
OASL	2.864439957	5.921184752	2.95E-85	1.23E-82
ADAMTSL5	2.850323905	5.390414277	2.06E-54	3.53E-52
OTUB2	2.841937749	6.576097142	4.63E-96	2.52E-93
C6orf99	2.833262611	1.495024625	3.53E-18	1.12E-16
CCDC85A	2.830676537	2.451619351	8.17E-19	2.71E-17
TTC9	2.819981162	1.368336424	1.45E-13	2.93E-12
LY6G6C	2.811676551	-0.765697086	7.86E-07	5.59E-06
SLC6A9	2.810426175	7.087053789	3.47E-44	4.39E-42
C6orf15	2.799450242	0.351910124	2.12E-11	3.34E-10
PABPC3	2.791981182	8.535515336	1.25E-16	3.39E-15
CD52	2.772690294	-1.822447057	0.047722773	0.0836167
POTEF	2.772049099	4.996387877	2.83E-16	7.47E-15
IFITM10	2.767412901	4.017951298	3.97E-31	2.90E-29
SUSD2	2.762363011	0.628283285	3.29E-13	6.32E-12
TTLL6	2.75773893	1.037875624	6.87E-14	1.45E-12
TNFRSF9	2.754985914	4.294706837	3.41E-30	2.39E-28
MARCKSL1	2.735462948	6.653212719	7.04E-90	3.53E-87
GJB3	2.72279713	6.4608397	1.54E-76	4.28E-74
SSX7	2.694241025	-0.514467974	3.68E-07	2.83E-06
CRCT1	2.68390548	-0.836676495	1.89E-05	9.49E-05
HSPB8	2.680679391	7.680386993	1.23E-55	2.20E-53
BIRC3	2.676185941	6.181412278	2.59E-06	1.62E-05
HSPA2	2.671905624	3.017096622	1.10E-24	5.56E-23
TSPAN15	2.659050835	5.667546351	1.46E-53	2.40E-51
OTOL1	2.649329068	2.777349833	7.85E-05	0.0003356
NXNL2	2.644365365	1.416380899	2.72E-18	8.79E-17
FABP3	2.64064739	1.484494838	1.87E-17	5.50E-16
PHETA1	2.616523132	6.266727405	3.03E-50	4.51E-48
TPD52L1	2.614016538	3.322902409	4.29E-27	2.64E-25
ZNF488	2.613460372	3.11777412	1.34E-26	7.96E-25
TTC22	2.610146123	1.107825461	2.10E-15	5.13E-14
LANCL3	2.604179348	1.098649003	7.97E-14	1.66E-12
UNC13A	2.593568112	4.372213932	1.60E-40	1.79E-38
POTEE	2.586882728	6.461051881	4.48E-24	2.11E-22
IGFBP6	2.578158723	5.208772959	3.76E-24	1.80E-22
MEFV	2.577508332	-0.753428463	1.43E-06	9.58E-06
LGALS9	2.569762127	1.617770209	1.84E-14	4.13E-13
CSAG1	2.568455815	4.568955401	4.52E-13	8.56E-12
PLAC1	2.563130055	1.636462708	6.82E-12	1.15E-10
MISP	2.555936645	3.113293105	1.34E-16	3.65E-15

CCNA1	2.551400893	6.362992913	3.45E-77	9.81E-75
KRT17	2.548912184	3.147646717	5.27E-22	2.22E-20
KRT19	2.545347171	10.219721	4.85E-87	2.25E-84
PTH1R	2.542098161	1.821766394	2.77E-18	8.91E-17
GPR132	2.538428893	2.421121537	5.56E-16	1.43E-14
ARRB1	2.53597895	6.583175141	2.81E-76	7.65E-74
CPM	2.533792817	3.703270711	1.36E-16	3.68E-15
FAM83A	2.526789661	4.669367968	7.85E-34	6.64E-32
CTSD	2.521798452	9.719643853	1.29E-39	1.40E-37
PAPSS2	2.513722962	8.958774954	6.50E-37	6.56E-35
SCLY	2.510094495	-0.249747403	0.003417939	0.0088334
KIF4B	2.497854891	4.061416412	8.96E-09	9.41E-08
SLC52A3	2.482961077	3.752087923	1.41E-20	5.31E-19
CPN2	2.476576222	-0.289968492	1.10E-07	9.53E-07
THBS2	2.469183589	8.005643906	8.97E-86	3.87E-83
IFIT1	2.467306964	4.675599471	2.01E-10	2.75E-09
PCDHB16	2.451437929	2.351619233	3.31E-18	1.05E-16
CISH	2.431615515	2.582772225	1.14E-18	3.75E-17
DPP4	2.429608928	2.077764814	5.70E-07	4.19E-06
NCF2	2.427768087	4.687977922	1.42E-20	5.35E-19
GPC1	2.42732385	6.653560701	7.76E-34	6.61E-32
CGN	2.425663526	4.00633001	1.11E-26	6.62E-25
KLF17	2.422153882	3.132073505	7.00E-21	2.69E-19
MALL	2.419322158	3.277586111	3.21E-09	3.65E-08
RHOD	2.406253302	7.26951948	3.54E-33	2.88E-31
MAGEA12	2.39257255	5.677841848	2.03E-12	3.60E-11
TRPM2	2.382839652	3.222741066	2.36E-19	8.24E-18
SLCO3A1	2.379915796	5.335141932	6.55E-44	8.12E-42
HMOX1	2.369938395	7.34779763	1.90E-79	5.94E-77
MIR27A	2.365776569	-1.288351183	0.000677362	0.0021969
PTX3	2.35954871	8.000350701	3.89E-78	1.19E-75
PRR16	2.356852764	1.926007196	8.96E-14	1.84E-12
SEC14L6	2.355844799	2.125932179	4.05E-11	6.20E-10
NLRP2	2.348168141	5.190701132	1.30E-21	5.28E-20
MYH15	2.344923066	-0.923125717	3.36E-05	0.0001592
TGM1	2.344151659	1.607095786	1.23E-12	2.22E-11
PHOSPHO1	2.32206316	1.00356928	1.91E-11	3.02E-10
TRAF1	2.313721562	7.251977571	4.28E-86	1.91E-83
PORCN	2.312802926	6.189308171	1.23E-50	1.87E-48
C3	2.302982778	9.496717662	1.95E-69	5.07E-67
SLC9A3R1	2.295439647	8.269462772	2.23E-61	4.81E-59
SLC22A1	2.292149974	0.035879239	2.41E-08	2.35E-07
CDH2	2.290747919	3.340336452	1.75E-08	1.75E-07
B3GNT3	2.290463906	5.930222922	1.08E-48	1.52E-46
COL17A1	2.290286471	6.211744343	4.37E-14	9.42E-13
C1QTNF6	2.288273208	5.126722259	6.98E-47	9.40E-45
CCDC80	2.281197305	6.42763541	2.84E-35	2.59E-33
PRSS22	2.278662216	1.499568908	1.70E-10	2.36E-09
ELFN2	2.269163211	6.010730199	8.87E-49	1.28E-46
TPGS1	2.266848577	3.995745063	1.72E-06	1.13E-05
KCTD11	2.266672945	6.244580884	7.62E-63	1.77E-60
ARMH4	2.264533629	2.33620073	1.54E-08	1.56E-07

TGM2	2.25061081	12.65077323	5.62E-78	1.64E-75
PCDHB2	2.24419367	2.359317137	3.59E-13	6.88E-12
SDK1	2.243288234	3.464966322	1.13E-22	4.98E-21
PCDHB9	2.234847443	0.58773104	3.72E-06	2.22E-05
RAB5IF	2.229493086	5.674129853	1.88E-14	4.21E-13
EHD1	2.224262634	10.04819741	8.39E-42	9.81E-40
H3F3C	2.212693479	5.266115001	1.31E-09	1.58E-08
ISG20	2.209478565	5.279772557	3.80E-33	3.07E-31
ANKRD2	2.20656133	0.286399702	0.000150866	0.0005943
FGF11	2.191922935	3.080217707	4.66E-18	1.46E-16
SAMSN1	2.187253844	3.483725744	0.000474098	0.0016076
ARL4C	2.185906592	7.394618121	2.20E-80	7.24E-78
PRR29	2.183411636	1.635428913	8.97E-09	9.42E-08
PRDM1	2.175318895	2.907912219	1.03E-11	1.70E-10
YPEL4	2.167699308	1.975332534	6.72E-15	1.58E-13
SDC4	2.161799329	10.30202167	5.46E-78	1.63E-75
RAB17	2.149001675	2.761583363	7.38E-07	5.28E-06
OAS1	2.145058129	2.347818423	1.21E-16	3.31E-15
P2RX6	2.138871136	0.323514735	3.53E-08	3.36E-07
GRIK2	2.137176363	0.58406287	1.04E-06	7.21E-06
ISG15	2.12110019	8.29229541	3.14E-23	1.44E-21
SLC25A42	2.119854719	4.297947497	2.50E-26	1.44E-24
CDON	2.115229916	3.693061095	4.13E-22	1.76E-20
S1PR1	2.113321379	0.985452177	1.06E-07	9.23E-07
MGAT5B	2.111426502	5.451026828	3.58E-32	2.77E-30
FMO5	2.11085827	3.053972408	8.84E-05	0.0003724
IRX5	2.106176848	3.953464269	3.56E-19	1.22E-17
HLA-DRA	2.105413479	-0.562194299	0.000179331	0.0006923
NMNAT2	2.091755088	3.435855067	3.70E-12	6.40E-11
NME2	2.085900871	3.534952213	2.67E-05	0.0001295
CSAG3	2.078374181	2.040470188	3.85E-08	3.64E-07
SMOX	2.072420219	6.70647175	1.02E-27	6.61E-26
GABRA3	2.064591422	1.511137227	2.82E-10	3.79E-09
KIAA0513	2.051339873	5.553662179	1.74E-40	1.92E-38
HAS3	2.048098502	2.563808444	1.20E-12	2.18E-11
MAOA	2.047977407	5.734841788	3.02E-09	3.46E-08
LRG1	2.047092141	1.447165499	1.21E-07	1.04E-06
CPA4	2.04673622	5.674558193	4.41E-33	3.54E-31
LAMA5	2.045412954	8.214009239	7.77E-23	3.46E-21
CCL20	2.035786107	-0.356590635	0.00145682	0.0042414
LYPD5	2.032064532	0.163012543	0.000242487	0.0009005
ZNF385A	2.02955807	5.013898136	2.04E-29	1.40E-27
NOL4L	2.026751714	4.242796084	3.46E-22	1.48E-20
FAM102A	2.023567713	6.116483248	1.36E-41	1.58E-39
TSPAN1	2.022183828	1.640599633	1.03E-12	1.88E-11
CRYBG2	2.017810546	4.36527921	4.87E-24	2.29E-22
JUP	2.013936112	7.173284709	6.24E-33	4.98E-31
EPB41L1	2.001974446	5.852267477	1.62E-39	1.75E-37
SLC1A1	1.999127538	1.271783498	1.53E-06	1.01E-05
GM2A	1.992681229	6.907932822	5.95E-30	4.14E-28
FMOD	1.984827516	1.289158649	5.20E-07	3.85E-06
ETV7	1.982004206	1.397790783	3.97E-10	5.20E-09

FRRS1	1.981081135	-0.572342702	0.000401036	0.0013958
MAGEA2B	1.975137023	3.498575363	8.40E-10	1.04E-08
TP53I3	1.973658542	3.226189645	9.18E-15	2.12E-13
DSEL	1.973211904	2.498261636	9.93E-06	5.36E-05
MAGEA2	1.971149091	3.541699363	1.59E-09	1.89E-08
BICDL1	1.969919228	2.80314941	6.69E-15	1.58E-13
SSX4B	1.968530155	-0.458348006	0.004209091	0.010545
GNGT2	1.965576184	0.72272793	2.29E-08	2.24E-07
ANOS1	1.964030056	5.848943222	9.09E-18	2.75E-16
TMEM102	1.953829299	4.355170562	4.61E-23	2.11E-21
MRPS24	1.951912197	2.198710878	3.32E-06	2.00E-05
TFAP2C	1.946810363	6.089768094	2.22E-39	2.36E-37
MIR138-1	1.925715388	-1.784555204	0.012172846	0.0261169
MLLT11	1.924142443	4.927164881	3.55E-14	7.71E-13
UBE2NL	1.920601781	1.491897913	0.00018372	0.0007065
ADGRD1	1.916495397	4.117017333	3.59E-21	1.40E-19
UCN2	1.914358435	-0.503205034	4.50E-05	0.0002053
CD22	1.901958711	3.892972172	6.19E-21	2.39E-19
USP43	1.890466292	4.693496907	3.20E-24	1.55E-22
CHI3L2	1.888904104	-0.446425967	0.001042702	0.0031737
SCAT1	1.885871289	-0.268939992	0.001005392	0.0030721
ICAM1	1.885428711	10.09576237	2.44E-59	4.78E-57
CSAG2	1.885138919	1.427655798	1.25E-07	1.06E-06
SMAD7	1.880544796	4.657249886	5.43E-26	3.04E-24
PLCD3	1.878092846	7.196446748	1.22E-48	1.69E-46
CLIC3	1.874936689	5.098286706	1.77E-14	3.99E-13
KLHDC9	1.870274094	-0.124264869	3.51E-05	0.0001649
TMEM160	1.866661009	4.832737008	3.74E-07	2.86E-06
DEPP1	1.864890503	3.113228001	3.50E-16	9.14E-15
MEGF6	1.859067954	5.177026403	1.11E-17	3.34E-16
ULK1	1.851992528	6.325660133	8.71E-21	3.32E-19
MXD1	1.844753631	4.974090289	8.54E-18	2.60E-16
CDH18	1.836552678	0.158562452	0.000627404	0.0020524
RBM47	1.834692138	3.626751661	5.28E-15	1.25E-13
LFNG	1.82830161	4.275916032	4.56E-10	5.89E-09
PSG9	1.827963743	1.527371158	3.07E-07	2.41E-06
RPS6KA2	1.827390181	6.8087263	2.75E-35	2.54E-33
ABHD8	1.826291462	5.962966742	6.52E-17	1.82E-15
CT62	1.818639438	4.489355914	1.87E-11	2.97E-10
MIEF2	1.818206064	6.025290067	1.87E-33	1.54E-31
EIF5AL1	1.811773028	8.741638671	7.12E-13	1.32E-11
FN1	1.811559313	11.67137094	1.04E-21	4.25E-20
TCIRG1	1.797594159	6.936164498	1.39E-22	6.12E-21
DCST2	1.796095457	-0.050149498	2.75E-05	0.0001331
CH25H	1.793716449	0.066526259	2.44E-05	0.0001192
SERINC2	1.791254557	7.928912444	2.89E-32	2.25E-30
APOL1	1.778266058	5.41798463	1.98E-27	1.25E-25
SPINT1	1.773113978	7.545696799	3.12E-35	2.81E-33
FIRRE	1.770033314	1.549735084	1.12E-07	9.71E-07
IRF1	1.768476158	6.281864161	2.11E-24	1.04E-22
CASTOR2	1.768334743	6.162371044	3.89E-34	3.38E-32
SLC35F6	1.758309128	7.740534958	5.71E-52	9.16E-50

SLCO4A1	1.757339355	1.01645266	2.62E-06	1.63E-05
FHDC1	1.753698838	2.336967534	4.26E-11	6.50E-10
RDH14	1.753383737	0.822822218	2.24E-06	1.42E-05
LRRC15	1.752184169	3.061716617	4.94E-11	7.48E-10
DLL4	1.752127554	1.557929618	3.27E-09	3.70E-08
SOX4	1.747121698	2.224112333	5.49E-11	8.23E-10
CXXC5	1.746899617	4.205074827	1.57E-17	4.67E-16
TRANK1	1.743498634	3.108662226	6.41E-11	9.52E-10
TAS1R3	1.74261337	3.936156433	7.62E-11	1.12E-09
SMAD9	1.736337738	2.12544093	8.64E-08	7.65E-07
ANXA9	1.734685577	2.93944575	2.53E-13	4.92E-12
B3GNT7	1.732820627	2.015097253	4.69E-11	7.13E-10
SAMD9	1.731414478	0.935764177	0.004724134	0.0116512
CCDC71L	1.73085507	5.782180398	1.21E-35	1.15E-33
SYT12	1.727060655	2.446321697	5.99E-09	6.45E-08
METRNL	1.724913624	5.201055304	2.09E-15	5.10E-14
PTPRH	1.719583096	5.885757567	1.29E-27	8.23E-26
KRT15	1.716575519	4.65951827	1.63E-21	6.60E-20
CCL5	1.716219521	0.073112034	2.65E-05	0.0001288
FAM43A	1.71510418	3.651103617	1.24E-12	2.25E-11
LINC02762	1.710178418	0.364446621	1.84E-06	1.20E-05
COX7B2	1.7101665	2.32513351	1.63E-06	1.08E-05
CDKN1A	1.707872152	9.960632684	4.74E-55	8.36E-53
TICAM1	1.707104204	6.429457438	9.77E-26	5.34E-24
RASGRF1	1.70615707	4.726033783	6.40E-22	2.66E-20
NOXO1	1.701508116	1.70331908	8.09E-06	4.48E-05
SPTBN5	1.700303769	1.456692879	3.30E-07	2.57E-06
C6orf132	1.700158789	8.182029172	8.20E-31	5.87E-29
LCTL	1.696251586	0.518734134	2.23E-05	0.0001099
BMP8B	1.69228896	5.891931459	4.48E-29	3.05E-27
PRRG2	1.691640435	2.005343125	1.08E-07	9.33E-07
EPHA4	1.688153526	-0.075820375	0.000182001	0.0007011
FOXC1	1.68596665	5.440150744	1.33E-29	9.17E-28
SPIRE2	1.68522091	5.16923824	8.68E-25	4.47E-23
COL4A4	1.678261437	0.969624004	0.000311127	0.0011176
CYP27B1	1.674727252	0.981271109	3.49E-08	3.34E-07
TM4SF18	1.674085275	-1.233364913	0.005618049	0.0135151
SEC14L2	1.673669243	4.991655359	2.09E-25	1.11E-23
DGAT2	1.671598269	3.949103576	4.31E-18	1.36E-16
PAG1	1.670822335	-0.358933098	0.003356003	0.0087075
PODXL	1.670109374	9.784225959	3.18E-28	2.06E-26
MYO5B	1.661103535	2.519766189	4.25E-09	4.71E-08
MMP24OS	1.6586823	3.61215117	1.90E-05	9.51E-05
COBL	1.657304182	2.871513372	7.68E-07	5.47E-06
SERPING1	1.654601613	2.523538106	9.24E-11	1.33E-09
ESR2	1.65033334	8.231081942	1.78E-07	1.46E-06
C1orf226	1.648567677	4.708521063	7.07E-23	3.17E-21
FBXO16	1.647758434	0.315987119	8.84E-06	4.84E-05
SYNE3	1.647080905	4.899218137	9.38E-23	4.16E-21
CLIC5	1.644914726	1.494028382	1.57E-06	1.04E-05
DHFR2	1.643364285	0.76209287	0.000508926	0.0017045
NPEPL1	1.64086394	1.34476332	0.026986229	0.0515158

CCDC149	1.640673787	1.510824601	6.42E-08	5.82E-07
GDPGP1	1.638424456	-0.110817255	0.000458414	0.001562
MYO18A	1.638406607	7.125895128	9.74E-32	7.29E-30
MMP24	1.637524061	4.070579939	4.54E-16	1.18E-14
CABLES1	1.636918941	6.996834792	3.55E-41	4.03E-39
SEMA5A	1.629851217	3.012870111	4.55E-08	4.25E-07
PIGM	1.629699218	1.702900563	6.18E-07	4.50E-06
SIN3B	1.626122794	8.043157567	3.72E-37	3.82E-35
FHL2	1.625315857	8.718460732	1.11E-30	7.91E-29
HLA-G	1.625287713	2.332168668	9.31E-05	0.0003897
RAET1L	1.623560148	1.203863891	4.06E-08	3.82E-07
SLC19A3	1.62303466	1.403429138	9.07E-07	6.36E-06
TEF	1.621244712	3.915686539	9.24E-13	1.70E-11
MAGEB1	1.616473864	3.124770549	7.44E-07	5.31E-06
ULBP2	1.611303504	4.883274639	3.87E-18	1.23E-16
PKP3	1.608758917	6.169601679	9.72E-12	1.61E-10
CARD6	1.607898242	4.098206027	5.15E-10	6.61E-09
USP18	1.599729929	2.038627632	1.23E-09	1.49E-08
MXRA8	1.599585551	1.506195332	4.44E-06	2.59E-05
N4BP3	1.599440306	2.235476478	1.10E-07	9.55E-07
C4orf48	1.59728433	3.527988631	0.000136006	0.0005438
NEDD9	1.59333705	7.740845129	5.90E-31	4.24E-29
LBH	1.593200572	1.552980902	3.00E-06	1.83E-05
FOLR1	1.592307719	0.404767688	8.80E-06	4.82E-05
HNRNPCL1	1.592137881	1.980426836	0.000205611	0.0007795
SLCO2A1	1.589136677	-0.47727108	0.000782617	0.0024842
USP41	1.587251971	-0.564132939	0.001806275	0.0051253
SLC45A1	1.587206114	1.593709408	8.67E-06	4.76E-05
MB	1.586194009	0.229189285	7.00E-06	3.93E-05
INAVA	1.585688404	5.91357273	1.07E-14	2.46E-13
MINDY1	1.585163751	4.05879728	5.19E-15	1.23E-13
PLEKHF1	1.582492609	5.272606884	5.12E-09	5.57E-08
RHOF	1.580703981	4.515998328	3.04E-09	3.47E-08
TENT5A	1.580436889	1.181279597	0.001185101	0.0035468
SHB	1.577646688	6.663888274	1.14E-23	5.29E-22
CLTB	1.577463127	8.561182146	7.35E-20	2.65E-18
HS3ST1	1.575999231	4.200641272	8.02E-12	1.35E-10
GINS4	1.573933427	7.038556766	2.26E-35	2.09E-33
LINC00511	1.572187286	3.545025241	3.17E-10	4.23E-09
LIPH	1.571616246	3.157415011	1.40E-11	2.27E-10
BMP8A	1.568704743	3.251463589	1.07E-08	1.10E-07
ANGPTL2	1.56826507	3.255050254	9.14E-07	6.40E-06
STX11	1.566898157	2.461568943	1.17E-09	1.42E-08
HSPB1	1.563425241	9.258081591	4.14E-21	1.61E-19
GALNT9	1.561337718	-0.323464381	0.007681448	0.0176351
TRIM29	1.559342941	1.001453223	1.18E-06	8.06E-06
IER5L	1.554501253	4.136014647	0.000367746	0.0012954
TAGLN2	1.553000232	9.804907499	2.41E-47	3.31E-45
TNFAIP3	1.547404965	8.020806964	4.48E-22	1.91E-20
TUBA3D	1.541627842	0.632661654	2.12E-06	1.36E-05
PCDHGB2	1.541267086	2.423805109	2.39E-09	2.77E-08
SPRY4	1.540882325	7.240433262	2.53E-32	1.98E-30

MANEAL	1.537619289	5.902178571	9.18E-26	5.04E-24
CASTOR3	1.537138299	3.998452265	6.79E-16	1.74E-14
TUBA8	1.535683972	0.362687839	0.000239594	0.0008916
MTHFS	1.530147607	1.997871113	0.000189385	0.0007255
DUSP23	1.529378724	4.641893911	9.32E-13	1.72E-11
DENND1C	1.528137211	2.778385861	1.62E-08	1.63E-07
ACOT4	1.526947546	3.22795264	2.12E-10	2.91E-09
SLC22A23	1.525127316	4.998333446	3.04E-21	1.19E-19
MAGEB2	1.521344311	6.23447367	6.93E-22	2.87E-20
PTGS2	1.521217537	6.070770182	9.97E-09	1.04E-07
C9orf116	1.519999624	2.198332966	6.43E-07	4.66E-06
GLUD2	1.515365208	5.976393125	6.05E-08	5.52E-07
MAGEA3	1.514265141	6.163253071	3.81E-09	4.27E-08
TOGARAM2	1.511850885	-0.757159889	0.018081473	0.0366935
UPK2	1.510293858	-0.382116034	0.000698027	0.0022534
CSF1	1.506238494	7.45627295	1.54E-26	9.08E-25
TAF1L	1.503960855	2.595175622	7.32E-06	4.09E-05
ACOT11	1.502276276	1.274413075	1.06E-05	5.64E-05
TMIE	1.50215995	1.692937616	5.57E-08	5.12E-07
C1orf53	1.50006283	0.603292795	1.07E-05	5.70E-05
NEK3	-1.501341968	1.842517567	0.003995943	0.0100796
CCZ1B	-1.501405019	4.671064034	1.27E-07	1.08E-06
KIF7	-1.50154653	2.883575324	7.45E-09	7.94E-08
USP34	-1.501818192	4.546131215	0.001467768	0.0042683
MOSPD2	-1.502275347	2.259079565	0.001266644	0.0037603
TRIM37	-1.502337315	4.360700625	1.26E-06	8.54E-06
CWC22	-1.502629619	2.291286382	0.001644092	0.0047164
TECR	-1.502648855	5.755060134	7.20E-18	2.22E-16
BTAF1	-1.502718101	3.697236108	0.000497016	0.0016698
RNF168	-1.502803103	4.201501672	2.32E-06	1.47E-05
FAR1	-1.503835663	4.052881062	3.75E-05	0.0001742
ZNF184	-1.503848413	1.165223761	0.038944178	0.07016
COL13A1	-1.504648128	6.052759108	1.98E-23	9.12E-22
PEX1	-1.504756891	2.52568263	0.000141577	0.0005627
RPS27A	-1.504983595	8.035988905	6.03E-06	3.43E-05
ZNF566	-1.505955283	1.160450883	7.11E-07	5.10E-06
CNOT6L	-1.506496099	3.365081852	0.000778325	0.0024724
ALKBH8	-1.506698964	2.213937698	0.00014991	0.0005913
SHPRH	-1.506933325	0.636719591	0.003849694	0.0097718
SOCS2	-1.507098393	2.050574935	3.23E-09	3.67E-08
PGM3	-1.508123734	4.297877824	1.58E-05	8.08E-05
NPIPB9	-1.508510385	0.49125641	0.000302402	0.0010891
PCGF6	-1.508592343	2.76626604	3.99E-07	3.04E-06
DDX1	-1.509937509	4.606592655	5.23E-05	0.0002346
NOC3L	-1.510609856	3.41046214	0.005205826	0.0126747
INTS2	-1.511770207	3.370926411	5.46E-05	0.0002432
LBR	-1.511847502	5.225936257	2.86E-06	1.77E-05
NUSAP1	-1.512189825	5.347269948	7.30E-08	6.54E-07
METAP2	-1.512489631	4.641817404	8.28E-08	7.36E-07
CCZ1	-1.512762546	4.618420905	5.52E-09	5.99E-08
ARHGAP5	-1.513376602	4.053373787	0.000510822	0.0017099
ADM2	-1.513971466	3.507650439	2.25E-10	3.07E-09

TTC37	-1.514053135	3.384826808	0.000134084	0.000537
PPAT	-1.514439816	3.69095042	9.37E-05	0.000392
PRPF40A	-1.514785156	4.770421575	0.000323656	0.0011549
ZBTB6	-1.514955635	1.726063125	0.000294136	0.0010649
NFIL3	-1.515424388	3.741085014	8.02E-11	1.17E-09
BCLAF1	-1.516189014	4.761583498	0.000105084	0.000433
RPS6KB1	-1.517158253	3.237246183	7.34E-06	4.10E-05
ADORA2B	-1.519194056	5.797508227	2.09E-19	7.31E-18
RNF138	-1.520185956	3.16214881	0.000504866	0.0016918
IL1B	-1.520225025	8.168770486	1.73E-28	1.15E-26
CPNE8	-1.520694679	1.71634373	0.002639418	0.0071136
HRNR	-1.520806926	3.131655825	0.000128616	0.0005184
NPIPB3	-1.521170428	4.109558226	4.24E-06	2.50E-05
CEP70	-1.521267288	-0.023963494	0.028970999	0.0546135
RAB38	-1.521483126	-1.491191027	0.019299835	0.0387762
HSPA5	-1.522608529	11.10938176	1.99E-22	8.66E-21
AARS	-1.523326644	8.345150727	1.41E-43	1.73E-41
CEP170	-1.523598744	4.777548017	0.000636514	0.0020795
CEP170	-1.523598744	4.777548017	0.000636514	0.0020795
MPDZ	-1.525756427	3.0367764	9.37E-06	5.08E-05
PAN3	-1.527651289	4.302204726	5.97E-06	3.40E-05
GNL2	-1.528179848	4.971908298	7.75E-10	9.66E-09
ANK2	-1.528228248	2.04054908	1.12E-06	7.72E-06
PDGFRB	-1.52842835	3.383667279	4.70E-13	8.87E-12
CCSAP	-1.528840316	3.217947204	2.22E-05	0.0001095
DHX36	-1.529419013	2.824958585	6.11E-05	0.0002689
CENPU	-1.52998048	3.131786132	9.89E-07	6.85E-06
PDLIM5	-1.530837168	6.128508114	1.12E-12	2.04E-11
ECT2	-1.530885517	4.23539205	0.000156431	0.0006129
SEC63	-1.53158769	4.373029002	1.33E-06	8.96E-06
TNFRSF1B	-1.532044122	4.36994896	7.56E-14	1.58E-12
CCL26	-1.532255492	1.700516202	1.66E-09	1.97E-08
RAB3IL1	-1.533568027	4.331224495	5.61E-12	9.55E-11
ADAMTS6	-1.533817455	5.71455432	3.32E-08	3.19E-07
LTN1	-1.534710434	3.102762064	0.013970327	0.0294733
AKT3	-1.535816527	4.660912294	6.68E-08	6.04E-07
SEPTIN7	-1.536738389	4.607871488	0.002226599	0.0061385
MIS18BP1	-1.53712159	2.719734955	0.003028432	0.0079817
CLDN12	-1.537821391	4.223769748	2.35E-06	1.48E-05
PUS10	-1.538141404	1.595745595	1.92E-08	1.90E-07
ZNF420	-1.538485993	0.761984143	7.67E-05	0.0003292
ANAPC4	-1.538618444	1.889572701	1.90E-05	9.49E-05
PPWD1	-1.539140157	2.233970248	7.34E-05	0.0003158
FSBP	-1.540437435	1.731349343	0.002664643	0.0071755
RAD54B	-1.540437435	1.731349343	0.002664643	0.0071755
TAF1D	-1.541280132	4.534971261	0.000882485	0.002751
MSANTD2	-1.541374285	2.829839643	1.31E-10	1.85E-09
POU2F2	-1.54166908	4.303927546	1.11E-17	3.34E-16
SLC5A3	-1.541877619	3.884370949	2.34E-05	0.0001149
DDX21	-1.542076374	7.145158276	1.38E-06	9.23E-06
KIF2A	-1.542146854	3.932965129	0.000251995	0.0009325
LRRC8C	-1.542451371	2.796500968	9.47E-08	8.33E-07

EML2	-1.543573466	5.260175707	2.38E-19	8.26E-18
PPP1R3C	-1.54439867	0.78002023	7.46E-06	4.16E-05
EGFR	-1.545783503	7.437038747	6.73E-19	2.24E-17
SMN2	-1.545831522	4.921636064	1.51E-09	1.80E-08
SMN2	-1.545831522	4.921636064	1.51E-09	1.80E-08
MCM8	-1.546095198	3.258303453	2.46E-05	0.0001201
RASA2	-1.546366982	2.737201053	3.10E-05	0.000148
RPL23A	-1.547802806	8.515959558	4.14E-07	3.14E-06
USO1	-1.548364012	5.382441727	1.01E-07	8.84E-07
MIB1	-1.549008058	4.506390329	0.000306688	0.0011033
TBC1D23	-1.549802612	3.230004338	0.000130575	0.0005251
NUP35	-1.549809503	2.434314038	0.000184899	0.0007105
ZSCAN12	-1.550818257	2.194448301	3.22E-07	2.52E-06
MYCBP2	-1.550835621	3.565361487	2.19E-06	1.39E-05
SLIT2	-1.553547748	4.870191857	2.13E-18	6.92E-17
WDR36	-1.554721023	4.311879567	4.73E-05	0.0002146
KTN1	-1.554925679	5.954162851	0.000398922	0.0013903
GPR68	-1.55508864	2.728522033	3.35E-11	5.17E-10
CAMK1D	-1.555332043	1.030805605	3.31E-08	3.18E-07
ARRDC4	-1.556543347	1.318373173	8.16E-09	8.62E-08
CDK7	-1.557079051	3.967825864	5.62E-07	4.14E-06
MYO1B	-1.557120898	5.14901343	3.07E-07	2.41E-06
FXR1	-1.559246548	4.71297991	0.000128091	0.0005165
SYDE2	-1.559519219	1.936814075	2.79E-06	1.72E-05
AIG1	-1.559958264	5.052882369	4.89E-15	1.17E-13
AIDA	-1.560386498	4.911174463	3.36E-09	3.80E-08
AGAP4	-1.560726909	1.824485081	1.51E-08	1.53E-07
TBCK	-1.560866761	0.21780612	0.000555767	0.0018392
ALDH1A3	-1.561229423	3.059073824	1.86E-08	1.85E-07
HEG1	-1.561335652	7.458247883	8.97E-25	4.60E-23
CCDC66	-1.562011002	0.24357708	0.003230295	0.0084328
DARS	-1.563285945	5.253126397	3.44E-06	2.07E-05
ZNF280C	-1.563899474	1.448400968	9.06E-08	7.98E-07
RIOK1	-1.563998262	3.67767022	2.13E-10	2.92E-09
FANCI	-1.564165007	4.988617418	2.31E-06	1.46E-05
RBMS3	-1.564940653	2.512658542	0.000130428	0.0005247
ABCB6	-1.56497029	2.916354127	1.05E-11	1.73E-10
PAIP1	-1.565166217	4.581731824	2.42E-09	2.80E-08
DOCK9	-1.566121542	4.194946655	3.36E-07	2.61E-06
LTV1	-1.566245472	3.635249338	7.54E-07	5.38E-06
GPN3	-1.566333624	3.219205103	2.51E-06	1.57E-05
AREG	-1.567325517	5.137168252	7.41E-26	4.09E-24
EEPD1	-1.568542583	2.382331252	1.94E-06	1.26E-05
TRMT11	-1.569432704	1.134069394	0.003304392	0.0085986
LMO4	-1.570073541	3.807346037	1.28E-11	2.08E-10
SESN2	-1.570990072	5.16544638	1.44E-13	2.92E-12
GMCL1	-1.571166456	2.752282228	7.22E-07	5.17E-06
GLI3	-1.57139644	3.047009014	4.32E-12	7.40E-11
MAP1LC3A	-1.57275024	0.35453359	2.66E-05	0.0001291
AC241401.2	-1.572958058	3.91157385	2.89E-06	1.78E-05
NPIPB4	-1.572958058	3.91157385	2.89E-06	1.78E-05
CCDC146	-1.574304977	-0.255085015	0.000151986	0.000598

MTBP	-1.574686429	1.744546164	6.14E-05	0.00027
SLFNL1	-1.574888668	1.786471745	2.08E-08	2.05E-07
KIF23	-1.575398829	5.693731982	1.68E-09	1.98E-08
VEPH1	-1.576295527	0.770986272	6.28E-06	3.57E-05
ZNF131	-1.57641864	3.398233081	1.03E-06	7.14E-06
POT1	-1.577206504	1.888035233	0.00129832	0.003834
BCAT1	-1.577909974	5.842647317	5.99E-10	7.59E-09
CTAGE9	-1.579029197	-0.256413212	0.000127471	0.0005144
RAD50	-1.579061221	3.960776719	1.74E-05	8.83E-05
ASGR1	-1.579264743	0.194681538	6.75E-06	3.80E-05
MBTD1	-1.582371057	1.767483852	2.46E-07	1.97E-06
SLC19A2	-1.582706889	2.803419971	3.21E-06	1.94E-05
AC007405.3	-1.582812497	1.177228796	8.03E-08	7.15E-07
ERICH2	-1.582812497	1.177228796	8.03E-08	7.15E-07
TFPI2	-1.583931147	4.476367485	1.82E-15	4.46E-14
NMD3	-1.585319637	3.003913457	0.00333901	0.0086796
FUCA1	-1.585456205	3.687329536	4.51E-13	8.56E-12
DIAPH3	-1.585531207	5.732738811	1.17E-09	1.42E-08
GAS2L3	-1.586190301	3.363272822	1.13E-05	6.01E-05
PXK	-1.586306422	3.743400756	5.11E-08	4.71E-07
CEBPZ	-1.586614312	3.789629621	0.000531872	0.0017683
SLC25A33	-1.587301053	3.691704885	2.22E-11	3.49E-10
FOXN2	-1.587961539	2.350789408	0.007261441	0.0168095
ITGA2	-1.591049568	5.259146551	5.52E-07	4.07E-06
FBXO43	-1.591676551	0.670993625	9.96E-06	5.37E-05
IPO7	-1.592976701	6.506647891	4.02E-05	0.000186
XPO1	-1.593163805	6.243739336	1.06E-05	5.67E-05
FAM76B	-1.593344433	2.079110905	2.56E-05	0.0001244
DLG4	-1.593467081	2.771140451	4.21E-12	7.22E-11
NCAPG2	-1.593620503	5.50397052	3.31E-11	5.11E-10
EIF4E	-1.595438507	3.472802401	0.0056413	0.0135658
CYB5R2	-1.595944842	0.854801482	3.81E-06	2.27E-05
MORF4L1	-1.597021663	6.26150957	0.000121011	0.0004901
GOLGA8A	-1.597928706	2.121870328	5.29E-07	3.91E-06
TOGARAM1	-1.597932421	1.853297387	1.35E-05	7.02E-05
LRPPRC	-1.598264992	5.367155823	7.96E-07	5.64E-06
SPDL1	-1.598814563	3.586007226	2.79E-05	0.0001348
RGMB-AS1	-1.598843684	-1.209614281	0.002423203	0.006612
ESCO1	-1.599106228	2.450048202	0.000478534	0.0016196
TMEM67	-1.599931055	-0.161756927	0.000280244	0.0010228
CLIP2	-1.600259279	4.927798901	8.46E-14	1.75E-12
CNTNAP1	-1.600287733	3.998938623	2.53E-16	6.69E-15
SYNPO	-1.601986076	4.812142135	3.66E-19	1.25E-17
PYY	-1.604247683	-0.532089436	0.000281053	0.0010252
DDX50	-1.604472751	4.012622739	1.80E-05	9.06E-05
TCEA1	-1.605407772	4.90802513	7.82E-05	0.0003347
ZNF569	-1.606474799	0.993523711	0.004603597	0.0114078
KIAA1109	-1.608168503	3.750237537	0.00019024	0.0007283
MRC2	-1.608801118	4.871153679	5.74E-14	1.22E-12
FCGBP	-1.609292113	3.348255245	1.27E-12	2.30E-11
DENND11	-1.610875366	4.190242089	2.17E-12	3.84E-11
CDC123	-1.612230143	5.161665735	2.81E-17	8.12E-16

FOSB	-1.612278158	2.206017648	0.000113645	0.0004637
LCORL	-1.612856673	2.061473362	2.33E-05	0.0001146
NBPF9	-1.614177828	5.815079243	3.15E-19	1.09E-17
EN2	-1.614648492	1.583237305	1.44E-05	7.43E-05
ALPK2	-1.617557206	4.445596413	6.07E-11	9.05E-10
RBPJ	-1.618288082	4.847372604	4.99E-06	2.88E-05
CD55	-1.618888935	5.429395577	3.22E-17	9.22E-16
DPM1	-1.61939034	3.335733811	0.000531679	0.0017683
PXYLP1	-1.621072003	0.11297025	2.31E-05	0.0001134
SYNE2	-1.62176858	4.017500141	5.84E-07	4.28E-06
ALDH7A1	-1.625303451	4.846681586	1.41E-11	2.29E-10
RECQL	-1.627379793	2.823152248	0.000478524	0.0016196
ADCY7	-1.627667944	5.407340875	5.28E-23	2.40E-21
EPRS	-1.628892817	6.402416837	7.12E-08	6.40E-07
TGFBR1	-1.629131171	4.694373625	1.53E-05	7.87E-05
KLHL15	-1.629139018	3.049092773	0.000188331	0.0007217
LRP3	-1.629367665	3.187378117	3.55E-05	0.0001668
POLE2	-1.629453962	2.444856364	6.32E-06	3.58E-05
CNTRL	-1.630655714	2.029094017	4.18E-06	2.47E-05
ARHGAP42	-1.634248773	2.968821929	0.001230282	0.0036671
USP24	-1.634615095	4.963161242	1.55E-07	1.30E-06
DNA2	-1.635632857	2.523544989	7.66E-05	0.0003288
NR4A2	-1.635674087	0.814199321	3.18E-07	2.49E-06
IFI16	-1.636669166	4.390390642	1.42E-05	7.36E-05
CASP8AP2	-1.637066194	1.620475925	0.00043409	0.0014946
CHD1	-1.637621814	3.527526442	0.000243831	0.000905
EIF2S2	-1.638445847	6.377255602	1.57E-08	1.58E-07
FOXP4-AS1	-1.639102493	0.516054125	4.43E-06	2.59E-05
CEP120	-1.639332041	2.958090781	5.68E-06	3.25E-05
EXOSC9	-1.641276418	2.890515416	1.02E-05	5.50E-05
PDP1	-1.641300801	5.072925991	1.79E-08	1.79E-07
PIGA	-1.641414215	2.444649644	0.003607879	0.0092461
CMTR2	-1.641857255	2.454716073	0.000758359	0.0024157
RBAK	-1.642287149	2.584764261	1.11E-05	5.91E-05
CEP85L	-1.643890943	0.508830638	0.000206691	0.0007825
WDR3	-1.643960669	4.882766051	7.27E-08	6.52E-07
ODC1	-1.644684589	7.259754314	1.65E-36	1.62E-34
TEX30	-1.645224922	1.713763208	0.009676823	0.0215253
BUB1B	-1.646751833	4.863589419	2.33E-11	3.63E-10
ATAD2	-1.648026793	5.072384657	9.75E-07	6.76E-06
CEP57	-1.648343784	3.845369027	4.34E-05	0.0001991
RB1CC1	-1.649044341	3.561504631	0.00065456	0.0021334
ZNF439	-1.649549016	0.654689105	2.37E-07	1.91E-06
DUS4L	-1.649615656	1.441435268	5.60E-06	3.21E-05
GPR89A	-1.650945601	3.142185697	2.44E-06	1.53E-05
PRKCI	-1.651155579	3.959677606	2.65E-06	1.65E-05
KRT33B	-1.651798787	0.398787603	1.13E-05	5.97E-05
NOL11	-1.652091739	3.834679499	0.000968584	0.0029771
KNTC1	-1.652804796	2.936826978	5.53E-05	0.0002462
LYAR	-1.653673074	4.74426649	3.55E-16	9.25E-15
PLCXD2	-1.653731753	-0.322515583	0.006451184	0.0151895
WNT2B	-1.653800058	-1.183826154	0.003895099	0.009867

POLQ	-1.655228228	1.611070688	4.17E-05	0.0001921
BRIP1	-1.65813721	2.770893286	0.000389301	0.0013591
ZCCHC7	-1.659423047	3.035404341	7.35E-11	1.08E-09
CEP112	-1.661046116	0.765617984	0.000169773	0.0006586
NAA15	-1.66117896	4.958328488	4.97E-05	0.000224
HSPD1	-1.663862555	8.153640801	7.59E-12	1.28E-10
TOP2A	-1.664070209	6.271972647	7.28E-06	4.07E-05
SLC40A1	-1.664073424	-0.820189437	0.000402194	0.001399
NAA25	-1.664426622	4.360360961	5.52E-06	3.16E-05
MAP3K2	-1.664745062	4.326056656	9.24E-05	0.0003872
DOC2A	-1.665964412	0.594327329	1.18E-06	8.09E-06
NUF2	-1.667637139	3.134502362	0.001716697	0.0048978
INSYN2B	-1.668788089	3.999481429	1.79E-08	1.79E-07
NPIPB11	-1.67092852	2.338804983	0.000109399	0.000448
NCAPG	-1.67142217	4.054260219	1.29E-05	6.77E-05
ZNF507	-1.672392853	3.101189203	3.15E-05	0.0001499
EIF2A	-1.67279073	4.024786738	2.80E-05	0.000135
ZFP69B	-1.673238853	0.89327024	7.09E-06	3.98E-05
NPAT	-1.67459602	3.116167645	0.000137162	0.0005477
GCNT4	-1.674644651	-1.195647511	0.045108898	0.079549
WDR35	-1.674730723	3.067252612	0.000136794	0.0005464
NRROS	-1.675004333	3.484998845	6.43E-11	9.54E-10
PNPT1	-1.675293537	3.454759206	8.70E-07	6.13E-06
PTPRU	-1.677632603	5.238464628	4.10E-19	1.39E-17
MYLK	-1.677722218	5.532649985	5.14E-20	1.88E-18
ZNF322	-1.678606922	2.661837802	5.09E-05	0.0002287
FERMT2	-1.679032867	5.205950248	1.01E-09	1.24E-08
KIF20B	-1.679361317	3.234669449	0.00499754	0.0122387
PIK3R1	-1.679449384	1.459893253	9.93E-07	6.88E-06
HS3ST3B1	-1.679620613	5.700111331	1.89E-34	1.67E-32
PHACTR2	-1.680144305	2.874668709	5.03E-05	0.0002262
HS3ST3A1	-1.680684278	2.242343133	1.25E-08	1.28E-07
SH3YL1	-1.681633882	0.016316558	1.34E-05	6.97E-05
INTS6L	-1.68200445	0.754163801	7.84E-06	4.36E-05
NT5C3A	-1.683367093	3.168092191	3.74E-07	2.86E-06
MAK16	-1.683400067	2.991344976	1.90E-07	1.55E-06
IFRD1	-1.684195157	4.309504389	1.93E-06	1.25E-05
FMNL2	-1.684230044	4.137785192	3.45E-08	3.29E-07
SENP6	-1.684277044	3.736834615	1.88E-07	1.54E-06
ITPR2	-1.684674993	3.291656044	1.87E-05	9.37E-05
RPS26	-1.684839734	5.821236357	1.19E-07	1.02E-06
LRRC34	-1.685396612	-1.082174346	0.002158742	0.005983
STEAP1	-1.686328334	-0.062443269	0.003158029	0.0082745
BIVM	-1.686996207	0.061941641	1.25E-05	6.57E-05
BRIX1	-1.687293661	4.291280304	1.10E-06	7.57E-06
PSIP1	-1.688923916	2.508891258	1.11E-06	7.60E-06
DOCK4	-1.690810362	3.502193748	1.07E-06	7.39E-06
PHF3	-1.691194816	4.770799526	6.95E-05	0.0003012
GAB1	-1.69213821	1.112492378	3.28E-06	1.98E-05
ZC3H6	-1.692195146	0.859696701	1.49E-05	7.64E-05
NACA	-1.692632805	7.044598811	2.06E-13	4.05E-12
THOC6	-1.692755436	4.678405031	5.90E-20	2.14E-18

LIMS4	-1.693511688	0.493574084	0.000143737	0.0005694
PKDCC	-1.694062309	0.61920322	2.56E-06	1.60E-05
ADAM11	-1.694650705	2.435681259	9.63E-10	1.19E-08
THNSL1	-1.695784675	0.991821597	6.47E-06	3.65E-05
KRTAP2-3	-1.696426862	2.744790916	1.65E-11	2.64E-10
PTCD3	-1.698898272	5.038065924	1.59E-14	3.60E-13
MPP7	-1.699110741	-0.885455005	0.001373188	0.0040364
AGPAT5	-1.699423537	3.950447709	1.27E-13	2.59E-12
NARS	-1.699561732	5.506499961	9.67E-08	8.47E-07
P3H3	-1.701681695	3.720903291	1.33E-13	2.71E-12
CDK17	-1.701996268	3.697873903	6.88E-05	0.0002984
PRKD3	-1.702787449	4.191272968	2.37E-06	1.50E-05
THOC2	-1.703489369	3.050652534	0.000208948	0.00079
ZMYM5	-1.704818281	0.863734894	6.09E-05	0.0002681
GPAM	-1.704963891	2.763080358	9.78E-10	1.20E-08
KLHL8	-1.705375428	2.945877746	7.37E-05	0.0003172
DUSP2	-1.706579363	0.801793988	4.37E-06	2.56E-05
RICTOR	-1.708930942	3.355122563	0.002378681	0.0065033
CEP290	-1.70964972	0.991973663	4.64E-07	3.48E-06
NPIPB5	-1.711257614	4.655806056	4.25E-07	3.21E-06
MTIF2	-1.711807093	3.980541704	2.92E-10	3.92E-09
NUP54	-1.712027461	3.587872532	3.05E-08	2.93E-07
ERBIN	-1.713047833	5.003975166	4.35E-05	0.0001994
PTTG1	-1.715304086	6.191999908	7.93E-18	2.43E-16
BUB1	-1.716230599	5.97139012	1.94E-09	2.27E-08
FRA10AC1	-1.716378832	2.424464437	1.59E-05	8.14E-05
NPIPA1	-1.716594389	2.428677347	1.19E-07	1.02E-06
CEP170	-1.716878339	0.691519496	0.000164554	0.0006409
CEP170	-1.716878339	0.691519496	0.000164554	0.0006409
CUL2	-1.717668376	3.670071753	1.41E-05	7.32E-05
ESF1	-1.7178745	2.207795393	0.000161719	0.0006315
AL353753.1	-1.719423233	-1.849871589	0.020917193	0.0415259
PSMC1	-1.721864501	5.664615772	5.10E-06	2.94E-05
SLC43A1	-1.723307318	4.450083631	2.80E-14	6.16E-13
ABLIM1	-1.723401868	5.128880486	1.61E-20	6.00E-19
DPH6	-1.723740699	1.365746136	0.000906978	0.0028119
PPIH	-1.724068253	4.475471891	2.19E-13	4.28E-12
LAIR1	-1.725780438	0.578068803	8.10E-06	4.48E-05
MRPL39	-1.727333935	2.903458163	4.39E-05	0.000201
NPIPA2	-1.727747886	1.058449352	0.002993219	0.0079049
SERPINE1	-1.728025263	10.02817912	2.69E-50	4.06E-48
SLC2A3	-1.72841003	3.533839527	3.87E-10	5.09E-09
CASK	-1.728975014	4.999819157	7.19E-13	1.33E-11
IL37	-1.731313569	-1.167627014	0.009186171	0.020536
ZNF146	-1.732449131	4.792082178	2.19E-06	1.40E-05
TMTC4	-1.73318257	0.781640135	5.04E-08	4.66E-07
SUV39H2	-1.736171068	2.930159888	7.95E-08	7.09E-07
CLDN1	-1.737280095	2.158533088	1.05E-11	1.73E-10
BATF3	-1.73944878	3.401489789	3.24E-16	8.51E-15
PDE8A	-1.739450326	4.114799009	1.02E-17	3.09E-16
ALDH6A1	-1.742699192	3.402925526	1.43E-13	2.89E-12
SCLT1	-1.742996673	1.821138412	1.45E-08	1.47E-07

FUT1	-1.743874649	4.59707757	1.91E-21	7.65E-20
CHORDC1	-1.74572317	3.879046676	0.000130405	0.0005247
GABPA	-1.746914844	2.056617785	9.89E-05	0.0004109
SENP7	-1.747207809	0.8659035	8.27E-06	4.56E-05
FOLR3	-1.747412714	-1.749449678	0.010812029	0.0236099
TUBD1	-1.747952333	0.895014818	3.98E-09	4.45E-08
NEDD1	-1.749037087	2.91139281	1.64E-05	8.36E-05
MND1	-1.749631489	2.524431361	8.29E-05	0.0003519
SLC7A11	-1.749731175	6.192071164	3.90E-09	4.37E-08
RHOT1	-1.750163356	2.872417613	3.50E-07	2.70E-06
CGB5	-1.751320761	-1.500091168	0.002010645	0.0056312
TBC1D3L	-1.751955501	0.993001516	4.42E-09	4.88E-08
TBC1D3L	-1.751955501	0.993001516	4.42E-09	4.88E-08
SMARCAD1	-1.752875641	2.961704794	4.83E-05	0.0002187
HBS1L	-1.753168541	3.298181246	2.95E-05	0.0001418
MYO9A	-1.753383671	3.782076504	2.10E-05	0.0001043
ANAPC1	-1.753475463	5.611534075	2.31E-20	8.51E-19
RAD18	-1.754219159	2.960415776	6.65E-06	3.74E-05
HSP90B1	-1.757277352	8.429626646	6.64E-10	8.35E-09
CENPF	-1.757429265	5.840679751	6.42E-06	3.63E-05
APPL1	-1.757802264	3.491588146	1.72E-06	1.13E-05
PTGES	-1.758313895	2.365181867	7.80E-08	6.96E-07
SUCO	-1.759634005	3.891951765	8.39E-07	5.93E-06
TBC1D12	-1.763482749	3.283054312	6.99E-08	6.29E-07
AOC3	-1.763570415	0.449470966	3.05E-05	0.000146
ARMC12	-1.76375948	0.954279563	1.06E-06	7.31E-06
MAD2L1	-1.764568939	4.491977061	0.002963971	0.0078432
SNX30	-1.767396775	4.104190555	1.07E-10	1.53E-09
GPR162	-1.768052448	0.165623363	0.000426896	0.0014739
TAGLN3	-1.768227565	-0.41735345	6.36E-05	0.0002782
ATF3	-1.772405812	5.072869269	1.49E-27	9.45E-26
SH2D2A	-1.773151875	2.527990475	1.00E-08	1.04E-07
EMC2	-1.773364483	2.205658151	0.000549379	0.0018219
ACVR2B	-1.774519704	3.008028486	2.94E-12	5.15E-11
NIPSNAP2	-1.774802323	2.472110632	3.79E-06	2.26E-05
SNRNP48	-1.777294638	2.656412822	2.94E-06	1.80E-05
YES1	-1.7772976	4.684975988	2.69E-06	1.67E-05
WDFY1	-1.778093421	4.512016964	3.67E-07	2.82E-06
PID1	-1.779357901	2.467894475	5.15E-14	1.10E-12
TBC1D3B	-1.78045062	-1.215539032	0.002882932	0.0076562
TBC1D3B	-1.78045062	-1.215539032	0.002882932	0.0076562
AC244489.2	-1.780915508	1.603617149	4.96E-09	5.42E-08
NPIPA7	-1.780915508	1.603617149	4.96E-09	5.42E-08
GTF2H2	-1.781183554	2.349119403	5.84E-07	4.28E-06
GTF2H2	-1.781183554	2.349119403	5.84E-07	4.28E-06
GTF2H2C	-1.781183554	2.349119403	5.84E-07	4.28E-06
ALOX5AP	-1.781735105	1.791057663	2.53E-10	3.41E-09
ZNF563	-1.78423908	0.305959973	2.14E-06	1.37E-05
IFT74	-1.784732558	0.851481245	4.04E-05	0.0001867
ANTXR2	-1.790064026	4.907249709	3.46E-11	5.31E-10
COBLL1	-1.791772583	1.907670384	4.33E-05	0.0001987
APLN	-1.792438788	3.234112011	6.59E-15	1.55E-13

CTNNAL1	-1.792762314	5.472883479	9.98E-06	5.38E-05
MAPK15	-1.796812458	-0.885229147	0.000203478	0.0007726
SLC37A2	-1.798015253	4.255766721	2.01E-13	3.97E-12
UBR2	-1.798138261	5.08597629	3.39E-10	4.49E-09
PPARA	-1.801017845	4.216295574	6.08E-18	1.89E-16
NSUN6	-1.801529005	1.338357751	4.07E-07	3.09E-06
LARS	-1.802058636	6.081085868	1.48E-10	2.06E-09
B3GNT5	-1.804083368	2.981937588	1.92E-05	9.58E-05
ANKRD36B	-1.806257943	0.956647901	2.84E-05	0.0001368
CDC26	-1.808035636	3.174863771	3.46E-07	2.68E-06
PLRG1	-1.814341661	3.978242435	8.89E-07	6.24E-06
CKAP2L	-1.81463781	4.625710679	1.99E-07	1.62E-06
PHGDH	-1.817385174	2.578566928	6.03E-12	1.02E-10
THAP12	-1.818866773	4.183899904	2.80E-06	1.73E-05
PDIK1L	-1.819819554	1.51767964	4.14E-06	2.45E-05
GPCPD1	-1.82203812	1.535299985	2.96E-08	2.86E-07
TMEM52	-1.822617278	2.310963739	1.23E-09	1.49E-08
RTL3	-1.823167847	-1.017421459	0.000286142	0.0010395
FABP5	-1.824190435	2.844833867	3.44E-07	2.66E-06
ATL2	-1.824950613	4.158841592	2.07E-06	1.33E-05
KIF14	-1.828051052	3.394156517	3.20E-06	1.94E-05
IVNS1ABP	-1.829814283	4.824035068	1.21E-14	2.76E-13
TOP2B	-1.832032899	4.242872119	9.21E-07	6.43E-06
TM4SF1	-1.832833534	7.101097514	4.91E-22	2.08E-20
NEGR1	-1.833625092	-1.563380168	0.003012789	0.0079455
RPS7	-1.833696325	7.451285393	4.62E-06	2.70E-05
JADE1	-1.835731223	4.223505408	7.91E-18	2.43E-16
PSMC6	-1.836652268	4.006463145	0.00560915	0.0135014
NPAS2	-1.837795486	5.437816765	2.47E-28	1.63E-26
CSE1L	-1.837886316	6.61915959	1.66E-06	1.10E-05
RASA1	-1.838212235	3.601873735	1.93E-09	2.26E-08
TSPOAP1	-1.839527612	3.425754552	3.88E-05	0.00018
NPM1	-1.840263536	8.206349839	1.76E-05	8.87E-05
PSMA3	-1.841012865	3.745780413	0.000831798	0.0026131
DEPDC1	-1.841399243	3.736688752	6.74E-07	4.86E-06
GTF2H2	-1.841419982	1.990816347	1.22E-05	6.40E-05
GTF2H2	-1.841419982	1.990816347	1.22E-05	6.40E-05
CDC37L1	-1.841548584	0.64146149	3.87E-08	3.66E-07
RBPMS2	-1.842650902	3.071158512	7.99E-14	1.66E-12
ERO1B	-1.842994457	1.722317835	1.52E-06	1.01E-05
GPBP1	-1.843138563	4.187458001	1.69E-08	1.69E-07
RCOR2	-1.844301104	-0.435142598	0.000138394	0.0005519
EPAS1	-1.845009185	6.564027448	5.30E-45	6.98E-43
RUNX2	-1.845728193	3.521315116	2.05E-13	4.04E-12
CEP128	-1.847601362	2.040070606	6.29E-11	9.36E-10
PAK1IP1	-1.849765109	3.789894932	4.90E-10	6.31E-09
BCL2	-1.850247425	1.716130097	1.28E-10	1.81E-09
ANKRD28	-1.851122508	4.342217551	1.71E-06	1.12E-05
BEND7	-1.852424792	3.968074422	8.64E-22	3.57E-20
NNMT	-1.85275902	7.302443236	1.98E-56	3.59E-54
CYP11A1	-1.856605269	2.95198407	1.13E-15	2.84E-14
ZNF684	-1.857169079	-0.502618053	8.44E-06	4.64E-05

SMURF2	-1.85780012	6.090639596	2.44E-14	5.39E-13
ZNF850	-1.860807011	3.152398228	7.06E-09	7.55E-08
CENPJ	-1.861078469	3.044614193	1.15E-07	9.90E-07
NDC80	-1.86354462	3.401610682	3.64E-06	2.18E-05
ABCE1	-1.866377171	5.132085004	2.37E-06	1.49E-05
ANKRD36C	-1.866872585	0.574376916	0.001175365	0.0035219
CENPE	-1.868504453	2.883967883	0.001218102	0.0036351
CREBRF	-1.868878701	1.593117645	0.00023252	0.0008695
NEK1	-1.869211212	1.580846416	1.61E-05	8.22E-05
ARHGAP11A	-1.869646859	5.05261512	6.55E-19	2.18E-17
OCLN	-1.869883028	2.68764202	2.54E-06	1.59E-05
NGFR	-1.870327083	-1.237099318	0.00057258	0.0018893
MMP10	-1.870801354	-0.572387557	0.000139018	0.0005541
SNRPE	-1.87312696	4.36544955	1.55E-05	7.96E-05
SLC4A5	-1.87554823	1.427847877	2.14E-11	3.37E-10
FANCD2	-1.879034162	2.987584468	7.86E-13	1.45E-11
ARHGAP29	-1.879424695	5.771093606	1.98E-05	9.88E-05
AC104971.1	-1.884571953	-1.130395715	0.000594536	0.0019556
DOP1B	-1.884660442	5.056939848	1.66E-30	1.17E-28
CAMK2D	-1.886611357	2.952077452	6.32E-09	6.78E-08
NUP107	-1.887152321	3.285603623	0.000113217	0.0004624
ZEB1	-1.887524227	2.702277807	7.21E-06	4.04E-05
HTRA1	-1.887692035	3.026132641	2.30E-13	4.47E-12
NADK2	-1.887940324	2.826387278	1.54E-08	1.56E-07
ZNF502	-1.889079957	0.15251821	1.03E-05	5.52E-05
NBPF26	-1.889946659	5.071364317	4.93E-10	6.35E-09
BLM	-1.890716424	2.847386563	3.21E-05	0.0001527
SEMA3F	-1.8940807	2.179380344	1.89E-08	1.88E-07
DBF4	-1.89641466	2.996195933	2.27E-05	0.0001118
NRARP	-1.896428144	1.526766878	6.48E-11	9.60E-10
C9orf72	-1.897255594	1.187700647	0.000703895	0.0022685
DEK	-1.898079458	5.814260822	1.31E-05	6.87E-05
PRMT9	-1.898180437	0.470806367	1.73E-08	1.73E-07
RGPD8	-1.899551274	2.745355899	0.001376988	0.0040438
NEDD4	-1.902140632	4.025223488	5.27E-06	3.04E-05
FRMD4B	-1.90280986	0.056894834	3.04E-07	2.39E-06
TBC1D15	-1.903356838	1.8995936	6.88E-06	3.87E-05
CRADD	-1.904045789	4.264860993	3.89E-24	1.85E-22
COL6A3	-1.904923247	2.567051059	8.69E-14	1.79E-12
ARMC4	-1.906381667	0.174351849	9.44E-08	8.31E-07
ZNF485	-1.913320886	0.454696687	9.76E-08	8.54E-07
CHST6	-1.913574411	2.342265086	5.43E-11	8.16E-10
DNMT3B	-1.913819024	2.984121879	9.80E-16	2.46E-14
TBC1D31	-1.915203662	2.009352339	3.09E-09	3.52E-08
SNRPA1	-1.91619824	4.762221371	6.76E-08	6.10E-07
PLK4	-1.919672194	2.508806209	3.43E-06	2.06E-05
RPL18A	-1.920058942	8.535303319	5.02E-08	4.64E-07
MIA3	-1.921413179	4.543088736	2.05E-11	3.23E-10
MTREX	-1.922081408	2.58221546	0.000299007	0.0010797
RPS3A	-1.925558079	8.305090476	0.000229113	0.0008588
COL15A1	-1.926381345	1.027095629	7.42E-09	7.91E-08
TBC1D3	-1.930873597	0.016633923	3.06E-06	1.87E-05

TBC1D3L	-1.930873597	0.016633923	3.06E-06	1.87E-05
TBC1D3L	-1.930873597	0.016633923	3.06E-06	1.87E-05
RSBN1L	-1.931533437	2.462281629	1.58E-06	1.05E-05
CCNA2	-1.935068349	5.228563322	4.68E-15	1.12E-13
SLC4A7	-1.936128759	3.134413403	3.63E-05	0.00017
OLAH	-1.937359506	-1.330072478	0.012919228	0.0275014
GRAMD2A	-1.937759246	0.976394217	2.20E-11	3.45E-10
GNL3	-1.938839201	4.566887231	1.05E-10	1.50E-09
CMSS1	-1.939078418	3.601507996	1.50E-10	2.09E-09
NR1D2	-1.939296983	3.607842353	2.37E-06	1.49E-05
PHF10	-1.939780387	4.333903608	4.42E-17	1.25E-15
CAPN11	-1.940906319	-1.787739794	0.006087942	0.0144743
FP236241.1	-1.942680173	3.200282071	9.98E-07	6.91E-06
WDHD1	-1.943621303	4.052535256	7.15E-07	5.12E-06
PLXND1	-1.946013003	5.39702183	2.40E-29	1.64E-27
UBA2	-1.947937277	5.390605661	1.65E-07	1.37E-06
HOXB9	-1.952213266	5.729684962	1.09E-44	1.41E-42
MIR100HG	-1.955653636	2.643515157	3.23E-06	1.96E-05
UAP1	-1.959578389	5.543996444	2.20E-08	2.16E-07
RPS6KA5	-1.963031171	0.924325774	1.13E-05	5.97E-05
HACE1	-1.965552467	1.109914789	2.97E-08	2.86E-07
TBC1D3I	-1.966806927	-0.056001675	5.60E-07	4.13E-06
TTK	-1.967280281	2.809480022	0.003704658	0.0094515
ATP11C	-1.968459583	2.896840642	3.72E-05	0.0001731
ZNF354A	-1.969082648	1.674798361	1.55E-05	7.94E-05
TBC1D3G	-1.970734132	-0.078035805	3.75E-07	2.86E-06
CGB8	-1.971369607	-1.736819742	0.002796692	0.00747
CCT8	-1.972290778	6.426434385	3.56E-06	2.13E-05
CU633904.1	-1.974842056	3.097386104	2.59E-06	1.62E-05
SEPSECS	-1.974887715	0.839598778	4.28E-10	5.56E-09
LONRF1	-1.97711825	2.59219654	8.50E-09	8.96E-08
LARP1B	-1.97848511	3.469982326	4.53E-13	8.56E-12
FAM169A	-1.978818626	1.758789943	9.76E-07	6.77E-06
NPIPB6	-1.979165819	0.037115135	7.29E-07	5.22E-06
ANKRD36	-1.979318553	1.136614733	2.02E-08	2.00E-07
RGPD4	-1.979569734	0.932213555	0.02265292	0.0443528
VCAN	-1.97967646	1.285027843	4.81E-05	0.0002175
EPGN	-1.980679284	-1.454582997	0.002726292	0.0073069
SMC3	-1.981245298	4.514131028	5.90E-07	4.31E-06
KIF13A	-1.983514156	4.805481545	2.40E-10	3.26E-09
SACS	-1.987385589	5.396117332	1.39E-05	7.20E-05
FGF5	-1.98989463	1.39297518	3.36E-13	6.44E-12
CNIH3	-1.990215325	2.474047624	1.68E-13	3.36E-12
AHCTF1	-1.991922897	4.50417695	2.01E-06	1.29E-05
DDIAS	-1.993727349	2.532647005	6.38E-08	5.79E-07
USP25	-2.000169296	2.669059456	1.09E-09	1.33E-08
LRIG3	-2.001985619	1.977014325	5.73E-10	7.27E-09
E2F7	-2.002505067	5.229938197	2.06E-16	5.49E-15
PUM3	-2.003508571	4.168953613	1.19E-13	2.43E-12
PDE7B	-2.006779628	1.546980983	1.29E-10	1.82E-09
MALT1	-2.007251357	3.139762548	8.90E-12	1.49E-10
TOX2	-2.011925862	2.791666359	1.21E-15	3.02E-14

UGCG	-2.012227604	6.677108338	5.94E-09	6.41E-08
SCML2	-2.016186326	1.862953811	8.61E-08	7.62E-07
SDAD1	-2.016482948	4.317328143	1.70E-15	4.21E-14
RPL39	-2.016716823	7.712790748	1.13E-09	1.38E-08
CENPC	-2.016927389	0.567401152	0.00018713	0.0007175
TUBE1	-2.018059665	2.071420777	3.08E-07	2.41E-06
AP1AR	-2.021485749	2.062599339	1.89E-05	9.49E-05
OXTR	-2.023100002	-1.467718455	0.000453246	0.0015476
TAF1A	-2.028194401	2.174841763	4.98E-06	2.88E-05
HUS1B	-2.030411207	-2.234062523	0.009777086	0.021702
DLGAP5	-2.041388106	5.331794371	1.55E-07	1.29E-06
DOCK11	-2.045356247	0.511975963	0.001028419	0.0031364
RCN1	-2.053037706	7.383902572	6.04E-20	2.18E-18
SGK1	-2.05552188	5.66234959	6.21E-27	3.78E-25
NEK2	-2.06323043	3.173559209	5.16E-09	5.61E-08
CRABP2	-2.063653018	2.676335439	1.17E-16	3.22E-15
NFYB	-2.072090453	2.064655197	2.71E-07	2.15E-06
CBWD1	-2.07250981	2.739025579	5.26E-10	6.72E-09
LIFR	-2.075806496	3.09691545	4.40E-06	2.57E-05
BRCA2	-2.078654253	3.524218969	5.46E-06	3.13E-05
STAG2	-2.087849549	3.461398088	0.000149169	0.0005887
COL8A1	-2.095004199	7.775660469	1.12E-50	1.75E-48
CCDC3	-2.096384798	0.1621099	5.55E-06	3.18E-05
MPZ	-2.096977389	-1.437800913	0.001144536	0.0034377
PSAT1	-2.099182212	6.965399894	2.56E-30	1.80E-28
ARG2	-2.107446449	3.12893341	1.85E-14	4.14E-13
SELENBP1	-2.109670604	-0.242243984	7.82E-07	5.56E-06
TEC	-2.11282642	0.759191341	2.40E-12	4.23E-11
TCIM	-2.112990818	-2.389450303	0.023625503	0.046041
ANKRD26	-2.116752005	0.433638341	1.14E-07	9.85E-07
MOB3B	-2.117162204	1.584690402	1.88E-11	2.98E-10
VLDLR-AS1	-2.122827018	-0.023787891	3.92E-07	2.99E-06
PLD1	-2.123709534	3.518628541	1.41E-12	2.55E-11
MSS51	-2.127388523	0.250064528	5.36E-10	6.84E-09
FRZB	-2.128147221	-1.361296519	0.001909212	0.0053796
RGPD6	-2.130916561	2.8793468	0.000703262	0.0022679
PRG2	-2.132635435	-0.63074653	3.64E-06	2.18E-05
TGFB2	-2.13296271	6.345233305	1.90E-24	9.42E-23
RPS18	-2.135200537	9.439249802	5.09E-08	4.70E-07
HAUS6	-2.135777909	3.118933368	4.38E-07	3.30E-06
G0S2	-2.139301908	6.890775647	3.14E-27	1.94E-25
CLPSL2	-2.140789715	-0.125759673	3.39E-08	3.25E-07
FAM133B	-2.150353856	0.390898543	1.34E-06	9.03E-06
SLC25A27	-2.153392065	-0.713311681	6.63E-06	3.74E-05
RPS13	-2.15528823	7.581556248	1.72E-12	3.06E-11
HSPA1B	-2.158735613	9.174292122	2.57E-34	2.25E-32
CD82	-2.1592372	6.141359164	9.12E-60	1.84E-57
FOS	-2.163763633	1.775252603	1.47E-14	3.32E-13
RGPD3	-2.164817003	0.99092783	0.000611584	0.0020064
MIA2	-2.165867795	2.849934158	8.41E-15	1.96E-13
ASPM	-2.166754131	3.87043152	2.48E-07	1.98E-06
OPN1LW	-2.170442958	-1.491514399	0.000437523	0.0015039

CADPS2	-2.170599552	0.234028391	5.50E-10	6.99E-09
TGDS	-2.172865349	0.11993039	3.22E-06	1.95E-05
ENPP1	-2.174746895	4.043858611	6.13E-14	1.30E-12
HES7	-2.175901353	1.80896332	2.62E-14	5.78E-13
CATSPER2	-2.178451223	-0.848850702	2.12E-05	0.0001051
TRAF5	-2.178770241	2.477788095	9.55E-16	2.41E-14
ARC	-2.179095816	2.333403469	8.27E-14	1.71E-12
ADAMTSL1	-2.179970121	3.59110042	2.08E-24	1.03E-22
TRIB3	-2.184218234	7.461516917	4.34E-35	3.88E-33
CU634019.1	-2.184301281	3.094603402	2.90E-07	2.29E-06
TFPI	-2.191864606	4.421872901	1.44E-07	1.21E-06
FLRT1	-2.191912691	0.474273266	2.23E-11	3.50E-10
UACA	-2.198248679	4.356000746	5.21E-12	8.88E-11
IPO11	-2.205121873	3.024641177	6.27E-07	4.55E-06
TCEA3	-2.206549666	3.859336	5.73E-26	3.19E-24
PTCH1	-2.207487003	2.842411209	5.88E-08	5.37E-07
FRMD4A	-2.212083352	3.976871269	3.55E-26	2.03E-24
SETDB2	-2.214590892	1.543436339	3.40E-12	5.92E-11
PEAR1	-2.21632237	3.101296939	4.56E-17	1.29E-15
HSPA4L	-2.222209036	2.624554747	4.56E-09	5.03E-08
FAM72A	-2.224328795	4.381476035	4.52E-15	1.08E-13
LIN9	-2.229527263	1.534284351	9.57E-07	6.65E-06
LEPR	-2.232405373	0.693353263	1.45E-08	1.47E-07
CBWD6	-2.233501756	2.24662855	1.03E-13	2.12E-12
SCG5	-2.234017446	1.561296566	3.52E-06	2.11E-05
NFXL1	-2.235076699	2.696171289	1.07E-10	1.53E-09
FAM72B	-2.236404262	4.450132201	3.18E-21	1.24E-19
MPP4	-2.236578095	1.643486254	3.91E-11	5.98E-10
CBWD3	-2.237719643	2.534261946	2.62E-07	2.09E-06
HGFAC	-2.238134862	-2.128172132	0.016039017	0.0331614
CCDC18	-2.245913361	0.757881643	1.66E-09	1.96E-08
LRP4	-2.246309477	3.004281033	1.76E-14	3.98E-13
ZNF501	-2.247075888	-0.045156056	7.80E-09	8.27E-08
XPOT	-2.247535956	6.03421607	3.63E-09	4.08E-08
ZNF449	-2.254209593	-0.0774381	4.34E-08	4.06E-07
TENM2	-2.254668472	7.390453694	1.18E-54	2.06E-52
PRKCE	-2.255903421	5.679071508	1.92E-25	1.02E-23
GEMIN2	-2.255907629	1.647130384	4.27E-07	3.23E-06
TBC1D3F	-2.259206564	-0.978068711	4.58E-05	0.0002083
PALM	-2.270702661	-1.170197945	0.000166754	0.0006481
CBWD2	-2.275018053	2.096365735	1.59E-13	3.17E-12
EXOSC8	-2.282314071	4.04052307	7.74E-09	8.22E-08
FAM72C	-2.282690526	4.42915319	2.25E-22	9.76E-21
BEX2	-2.291044247	2.201128527	1.89E-17	5.54E-16
MIR142	-2.293439442	-2.579245611	0.036099726	0.0658218
IBTK	-2.314086158	3.84764677	7.90E-09	8.36E-08
F2RL2	-2.318540598	-2.580961594	0.032799142	0.0606339
TAFA3	-2.319651411	-0.847729248	6.53E-06	3.68E-05
KNL1	-2.331776273	3.337211506	1.07E-06	7.40E-06
MPZL2	-2.332823342	4.795396564	9.67E-16	2.44E-14
CU633967.1	-2.335487204	3.227336719	6.37E-08	5.79E-07
AZGP1	-2.336076097	-1.149820141	2.43E-05	0.000119

DNER	-2.338352455	0.263873399	6.68E-10	8.38E-09
CBWD5	-2.340481115	2.807405329	8.21E-16	2.09E-14
SASS6	-2.342117096	1.36371942	1.38E-09	1.66E-08
HSPA1A	-2.343297212	8.826313884	5.50E-36	5.30E-34
LIMCH1	-2.348862295	7.137761925	1.35E-33	1.13E-31
FAM135A	-2.35274437	1.790109558	4.70E-07	3.51E-06
ADAT2	-2.363667687	0.892736129	3.91E-09	4.38E-08
ZGRF1	-2.374121787	0.194757871	1.63E-10	2.27E-09
PPFIBP2	-2.376533844	0.568517842	5.50E-13	1.03E-11
FAM72D	-2.377962737	4.126384496	1.15E-25	6.18E-24
HNRNPA1	-2.382458432	7.78230852	3.42E-12	5.95E-11
AL357075.4	-2.385072775	0.393068472	6.27E-08	5.70E-07
HK2	-2.386652603	5.674792973	1.94E-26	1.12E-24
SCN5A	-2.396743064	4.588409156	1.32E-19	4.68E-18
TTC39B	-2.402443416	0.717846137	1.29E-11	2.09E-10
CYTIP	-2.402473593	2.229737662	3.13E-13	6.05E-12
GAS5	-2.405588069	4.918912592	6.20E-07	4.51E-06
KRT86	-2.406924775	0.923188292	1.13E-05	5.97E-05
OPN1MW2	-2.418631262	-0.684461758	3.69E-07	2.83E-06
KIF21B	-2.419784906	2.992786277	5.27E-16	1.36E-14
ILDR2	-2.422483747	0.878566477	2.12E-13	4.15E-12
LRRC73	-2.423732019	-0.595648818	4.00E-07	3.04E-06
CDKN1C	-2.428606118	1.700765068	4.54E-20	1.67E-18
NFIB	-2.428694764	3.141343235	6.12E-11	9.12E-10
AHI1	-2.430992089	0.195953079	6.34E-05	0.0002773
TEX48	-2.434815164	-0.533409107	1.52E-07	1.28E-06
GPR65	-2.438400317	2.982084193	0.000172134	0.0006666
HSPA6	-2.438739732	4.471267731	2.50E-32	1.97E-30
CPNE7	-2.439177119	-1.200163136	7.34E-06	4.10E-05
NAMPT	-2.4407384	7.330195192	1.40E-09	1.68E-08
RPL7	-2.453436758	6.845777753	0.00024949	0.0009235
IL17RE	-2.467690756	1.252051884	2.43E-14	5.39E-13
KIF15	-2.471742058	1.203114691	5.21E-14	1.12E-12
NPIPA3	-2.47936487	0.774050113	5.75E-10	7.29E-09
WNT10B	-2.482405676	1.475586803	2.93E-16	7.71E-15
COLEC10	-2.492277609	-0.25636358	1.50E-07	1.26E-06
DCLK1	-2.494568525	1.351372663	1.62E-12	2.89E-11
MYCL	-2.495582386	1.999272409	7.34E-18	2.26E-16
RGPD5	-2.497185128	2.786175314	4.49E-08	4.20E-07
HECW2	-2.503307463	1.33773351	9.04E-12	1.50E-10
CSF3	-2.503995235	3.533283565	1.56E-17	4.63E-16
INHBE	-2.505026529	-1.423209902	4.20E-05	0.0001932
GOLGA6L10	-2.506836044	-0.354463417	1.79E-08	1.79E-07
SCFD1	-2.514719458	2.289884048	1.48E-09	1.77E-08
SUPT3H	-2.516163285	2.574159321	1.12E-17	3.36E-16
PDE9A	-2.547333293	0.531389998	4.32E-06	2.54E-05
ZNF117	-2.548333238	-1.725164163	0.000684064	0.0022175
DDIT4	-2.557792543	6.548492764	9.07E-27	5.46E-25
RAP1GAP2	-2.561424454	5.406999992	5.13E-32	3.91E-30
EXPH5	-2.562058745	3.139939459	2.10E-13	4.12E-12
LARGE2	-2.570634263	1.354814576	0.000283461	0.0010328
GK	-2.573114566	0.883314917	1.93E-09	2.27E-08

STK17B	-2.587100421	2.895358986	1.80E-15	4.44E-14
SERTAD4	-2.59744387	2.441388863	2.09E-21	8.33E-20
NME7	-2.603964217	2.915001329	1.50E-07	1.26E-06
RAPGEF3	-2.620903793	0.091537799	9.07E-11	1.32E-09
IL1RAPL1	-2.625016263	2.1728313	2.83E-13	5.48E-12
NAE1	-2.642560334	3.306642551	1.93E-07	1.57E-06
NAIP	-2.664075601	0.121960906	3.41E-11	5.25E-10
AIM2	-2.664900978	-1.649831994	0.000193235	0.0007382
AGTPBP1	-2.67508592	2.957109827	6.14E-12	1.04E-10
JDP2	-2.680698121	3.448895799	7.05E-26	3.91E-24
CSPG4	-2.702625638	2.619578716	3.70E-17	1.06E-15
PRTN3	-2.70459251	-1.662875136	4.14E-05	0.0001909
EDNRB	-2.70551363	-0.24432056	2.08E-09	2.42E-08
CES3	-2.720660939	0.447901319	2.67E-14	5.89E-13
RNF43	-2.722634452	3.481415997	2.26E-14	5.04E-13
TXNIP	-2.730402619	7.084328514	2.46E-65	5.91E-63
TATDN1	-2.732455296	0.031946414	0.000460321	0.0015677
DDIT3	-2.741760064	4.697836427	5.42E-26	3.04E-24
CCDC144A	-2.748177124	-0.68207208	2.99E-06	1.83E-05
CLMN	-2.75430713	4.363135833	3.72E-43	4.48E-41
RPL21	-2.76897658	6.506380488	6.45E-09	6.91E-08
LAMP3	-2.772516194	4.274141947	2.00E-39	2.14E-37
PLPP4	-2.776803348	1.967110135	6.76E-18	2.10E-16
RPS21	-2.787216258	6.356576614	3.30E-13	6.33E-12
DEPDC7	-2.788607482	1.65563054	7.77E-21	2.98E-19
RPS27	-2.788715165	6.54137724	5.56E-06	3.19E-05
KDM7A	-2.793787063	2.630183263	1.16E-16	3.20E-15
CRLF2	-2.804429763	-0.722606599	2.36E-08	2.31E-07
KRT83	-2.807666236	-1.430807624	0.000140332	0.0005583
BMPER	-2.811590045	2.467211507	5.39E-14	1.15E-12
FAT3	-2.818050413	2.600265025	1.41E-26	8.33E-25
SERPINB2	-2.81848307	5.857205931	3.17E-28	2.06E-26
GOLGA6L4	-2.837613004	-0.519415712	7.44E-08	6.65E-07
RRAD	-2.848883654	4.010723158	3.10E-39	3.26E-37
SAA4	-2.866089581	-2.329216098	0.002260634	0.0062145
RPL9	-2.876901147	7.063754305	8.65E-13	1.60E-11
RPS28	-2.879292022	5.826164177	2.93E-08	2.83E-07
CNKSR3	-2.879528494	2.745575134	2.48E-28	1.63E-26
GRAMD1B	-2.890834948	5.665366203	1.80E-52	2.93E-50
CPEB2	-2.904951416	2.117689113	1.21E-18	3.95E-17
SLC7A2	-2.919646337	3.752019134	9.38E-22	3.86E-20
GCNA	-2.921711669	0.07201882	1.06E-10	1.51E-09
LTF	-3.011495763	-0.942979134	4.20E-06	2.48E-05
OPN1MW	-3.022461113	-1.242043858	8.61E-07	6.07E-06
ATP6V0D2	-3.023326416	0.70320968	4.92E-18	1.54E-16
DMBT1	-3.024095313	3.149415717	1.74E-10	2.40E-09
TIE1	-3.054172944	-1.609792429	0.00017411	0.0006734
NRP2	-3.078989937	3.69575344	1.69E-37	1.75E-35
EPB41L4B	-3.117416822	2.662509987	1.03E-27	6.61E-26
C7orf57	-3.12382283	-0.44040792	3.37E-11	5.19E-10
NUPR1	-3.124703068	0.6038786	1.25E-18	4.07E-17
TBL1X	-3.162635071	6.120363908	1.61E-96	9.14E-94

PCCA	-3.180907339	-0.283534273	2.82E-12	4.95E-11
FHL1	-3.182161226	2.463173608	5.20E-23	2.37E-21
ASNS	-3.205429595	7.096444479	1.47E-81	5.11E-79
AASS	-3.227909537	3.139300128	4.27E-21	1.66E-19
CXCL5	-3.265360066	-1.517547749	2.10E-06	1.35E-05
MMP3	-3.293130416	4.419364212	6.27E-19	2.10E-17
NIBAN1	-3.303463445	5.283826535	1.04E-48	1.47E-46
MT1G	-3.350898012	-2.506666634	0.011092474	0.0241339
LCN2	-3.361518309	6.253163201	9.01E-85	3.53E-82
MKX	-3.382729395	0.083793582	3.29E-12	5.74E-11
AKAP12	-3.415334846	4.681676187	2.95E-67	7.53E-65
TNFSF15	-3.455158325	1.680575506	4.48E-26	2.55E-24
GABRE	-3.461327364	0.837195722	6.72E-11	9.95E-10
FBXL13	-3.474173121	0.249397804	1.84E-13	3.65E-12
KCTD4	-3.492898685	-2.443716355	0.003340747	0.0086798
CRIP1	-3.495144013	-2.442979725	0.003500742	0.0090214
PDE4D	-3.510598369	2.468928135	3.26E-18	1.04E-16
GEM	-3.527551211	2.755807817	5.65E-34	4.85E-32
KRT81	-3.539796467	4.51356485	4.44E-41	5.01E-39
TMCC3	-3.539860389	2.083562049	2.42E-19	8.36E-18
RGS2	-3.541676789	-0.462500361	1.05E-08	1.08E-07
ADCY10P1	-3.571804412	-1.925316103	1.78E-05	8.98E-05
SMIM11B	-3.57226138	-0.192405713	3.30E-15	7.97E-14
SMIM11B	-3.57226138	-0.192405713	3.30E-15	7.97E-14
MMP1	-3.607305361	6.689012516	9.09E-25	4.62E-23
IL11	-3.660492029	7.442551144	2.82E-179	7.07E-176
TLE2	-3.710561892	-1.184511478	5.30E-09	5.76E-08
CST7	-3.721022715	2.740544183	5.78E-40	6.35E-38
SARNP	-3.777159567	1.661217271	2.58E-21	1.02E-19
MCTP1	-3.800862863	0.773757479	1.47E-16	3.96E-15
BLID	-3.841615027	-1.761152815	2.48E-06	1.56E-05
TMEM71	-3.866516814	-0.586282147	2.23E-13	4.36E-12
CNN1	-3.888276665	-0.330219074	1.14E-09	1.39E-08
PAPPA	-4.044408891	2.101976829	1.19E-32	9.41E-31
SPNS3	-4.224749418	-0.778316766	2.62E-12	4.61E-11
PAGE2B	-4.361349794	-2.867299496	0.019934079	0.0398459
MTRNR2L6	-4.471293173	-1.879222409	7.74E-06	4.30E-05
SAA2-SAA4	-4.532783409	-1.837752973	4.28E-06	2.52E-05
IL24	-4.573019794	5.765791211	3.95E-154	7.07E-151
MIR125B1	-4.578203506	-2.786886716	0.013961718	0.0294651
MTRNR2L12	-4.594657096	0.275889775	5.75E-14	1.22E-12
GDF15	-4.635046849	1.301689685	1.70E-24	8.46E-23
KLF15	-4.668644924	0.963846603	5.36E-22	2.25E-20
MTRNR2L10	-4.756767703	-1.679893557	1.23E-06	8.35E-06
MTRNR2L8	-4.813830442	0.54727934	2.44E-11	3.80E-10
GOLGA6L3	-5.289893914	-1.28212111	2.43E-10	3.29E-09
HAS2	-5.409092726	3.184903549	2.33E-42	2.78E-40
PPIAL4F	-5.542351996	-2.321925701	0.000117506	0.0004768
FAM9B	-6.432783882	-0.308844509	1.78E-20	6.57E-19

Supplemental Table 4. Sequences of primers used for RT-qPCR.

Gene	Forward (5'-3')	Reverse (5'-3')
HPRT1	CGTCTTGCTCGAGATGTGATG	GAGCACACAGAGGGCTACAATG
ERβ	GATAAAAACCGGCGCAAGAG	TCACCATTCCCACTTCGTAACA
ALOX5AP	TGTAGATGCGTACCCCACTT	AGTATGATGCGTTTCCCAAA
CXCL2	TTGCCACCACCTATTAGCCA	TGTGTGGCAAGGACCTCTAG
IL1B	CCACTACAGCAAGGGCTTCAG	GAACCAGCATCTTCCTCAGCTT
IL6	CACAGACAGCCACTCACCTCTT	GAGATGCCGTCGAGGATGTAC
IL8	GACAAGAGCCAGGAAGAAACCA	GGCCAGCTTGGAAGTCATGT
IL11	ATCCCCCCGCCATTATCTC	CCACCCCTGCTCCTGAAATA

Supplemental Table 5. Antibodies used for western blotting (western blotting), immunofluorescence (IF), DuoLink™ (DL), co-immunoprecipitation (IP), and/or ChIP, company of purchase, catalog number, and dilution of primary antibody.

Protein	Vendor and Number	Use(s)	Dilution(s)
ERβ (#1)	Bio-Rad PPG5/10	IHC	1:150
ERβ (#2)	R&D Systems PPZ0506	WB, IP	1:500, 10µl/IP
β-actin	Sigma A2228	WB	1:10000
Vinculin	Abcam ab129002	WB	1:2000
Phospho-NFκB	Cell Signaling 3033	WB	1:1000
Total NFκB (p65/RELA) (#1)	Cell Signaling 8242	WB, IP, DL, ChIP	1:1000, 10µl/IP, 1:400, 2µg/ChIP
Phospho-IκBα	Cell Signaling 9246	WB	1:1000
Total IκBα	Cell Signaling 4812	WB	1:1000
Total-NFκB (p65/RELA) (#2)	Santa Cruz sc8008	IF, DL	1:100, 1:100
RELB	Cell Signaling 4954	WB	1:1000
ERβ (#3)	MC10	ChIP	Dilute 1:4 use 1µl/IP
EZH2 (#1)	Cell Signaling 5246	WB, DL	1:1000, 1:100
EZH2 (#2)	Abcam ab191250	ChIP	2.5µg/ChIP
EED	Cell Signaling 51673	WB	1:1000
SUZ12	Cell Signaling 3737	WB	1:1000
FLAG	Sigma F3165	DL	1:1000
H3K27me3	Abcam ab6002	ChIP	2.5µg/ChIP
RNA Pol II phospho Ser2	Bethyl, A300-654A	ChIP	2.5µg/ChIP

Supplemental Table 6. Sequences of primers used for ChIP-PCR.

Site	Forward (5'-3')	Reverse (5'-3')
ALOX5AP	TGGCCCTCTGACATTGCACTGCC	GGAATCAGGCCATGCAGAGCTGC
CXCL2	CAACTGTGGGATGTTCTCTTTCTGC	AATGCTTTCCAGAGAAGTAACTCCC
IL11	GGCTCTGAGGCTCTGTGCAGAC	AGAGCTCTTACCTGAGGCGATGAG

UNCROPPED AND UNPROCESSED BLOTS

Figure 1b

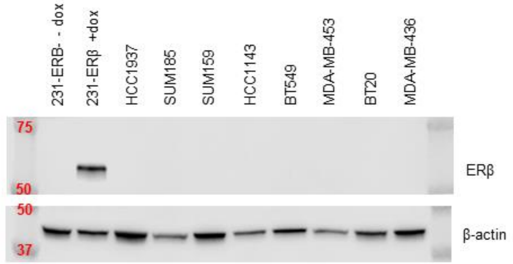

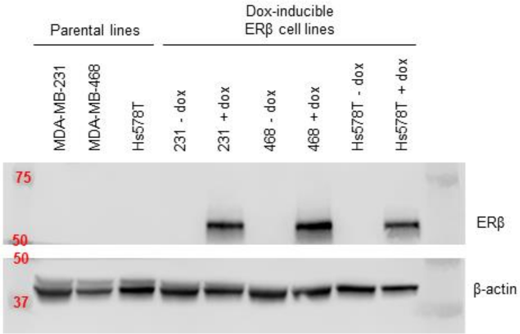

Figure 2e

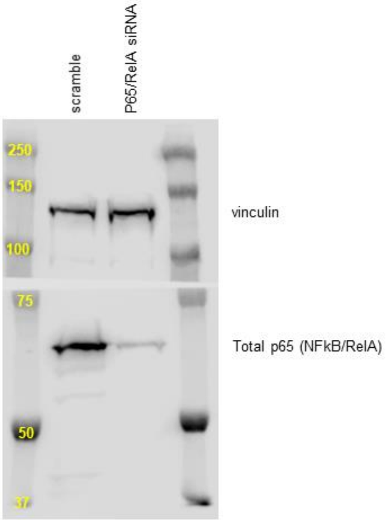

Figure 2g

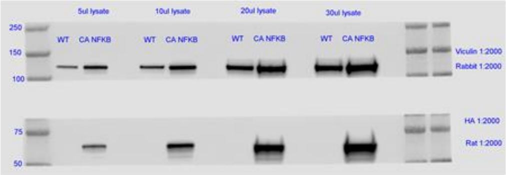

Figure 3g

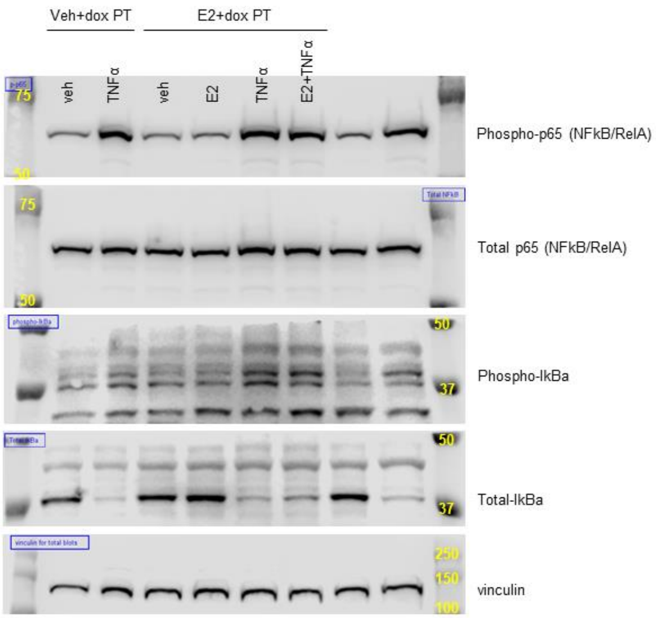

Figure 4g

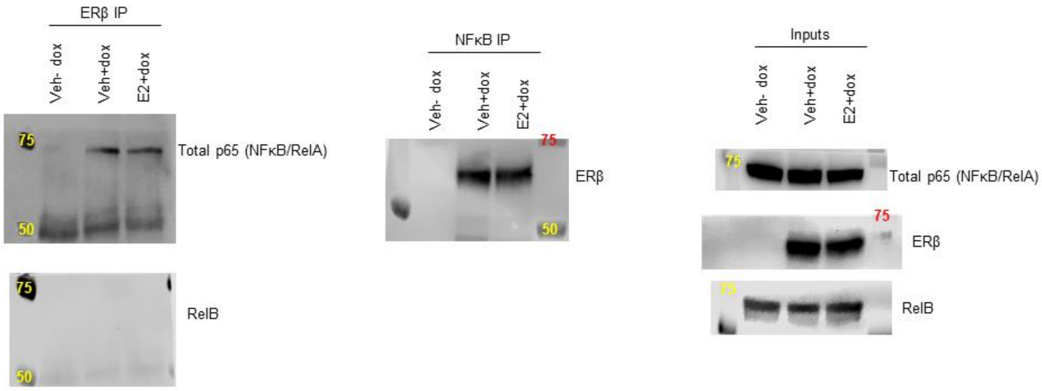

Figure 5c

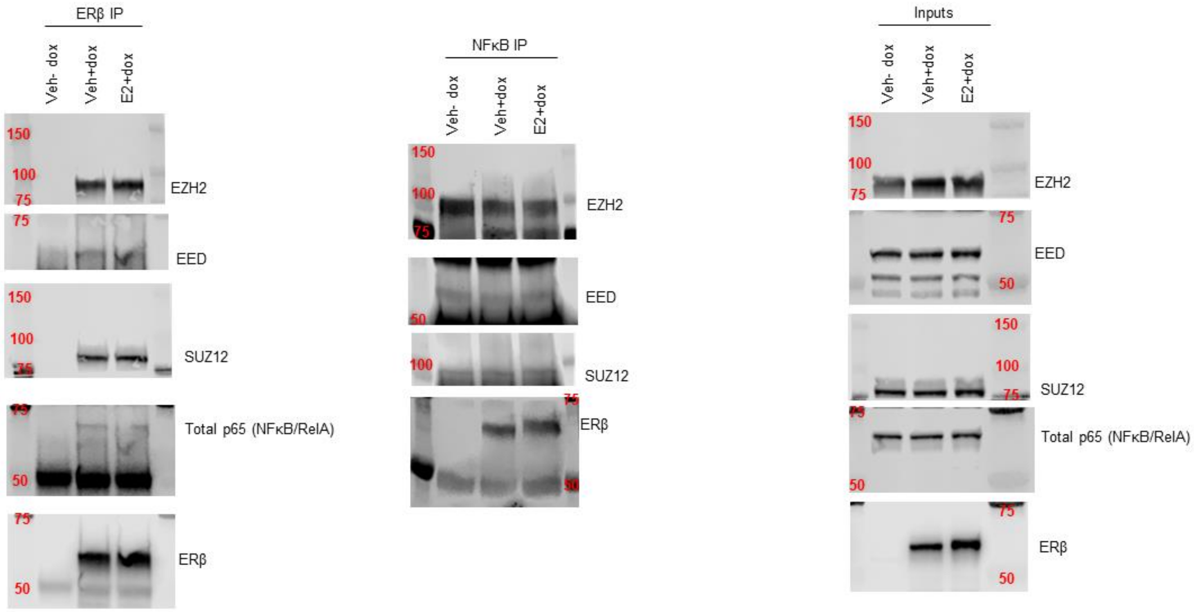
ERβ IP
Veh- dox
Veh+dox
E2+dox
EZH2
EED
SUZ12
Total p65 (NFκB/RelA)
ERβ
NFκB IP
Veh- dox
Veh+dox
E2+dox
EZH2
EED
SUZ12
ERβ
Inputs
Veh- dox
Veh+dox
E2+dox
EZH2
EED
SUZ12
Total p65 (NFκB/RelA)
ERβ
